# Ag(I)-Catalyzed Oxidative Cyclization of 1,4-Diynamide-3-ols with N-Oxide for Divergent Synthesis of 2-Substituted Furan-4-carboxamide Derivatives

**DOI:** 10.1021/acs.joc.4c02096

**Published:** 2024-10-22

**Authors:** Akshay Subhash Narode, Debashis Barik, Ping-Hsun Kuo, Mu-Jeng Cheng, Rai-Shung Liu

**Affiliations:** † Department of Chemistry, National Tsing-Hua University, Hsinchu, Taiwan 30013, ROC; ‡ Department of Chemistry, National Cheng-Kung University, Tainan 701, Taiwan, ROC

## Abstract

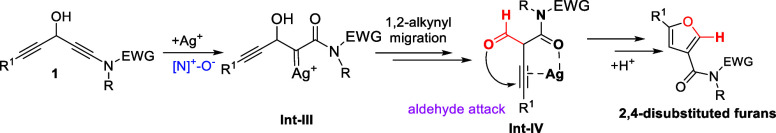

This work reports Ag(I)-catalyzed oxidative cyclizations of 1,4-diynamide-3-ols with 8-methylquinoline oxide to form 2-substituted furan-4-carboxamides. The reaction chemoselectivity is distinct from that reported in previous work by Hashmi. We performed density functional theory calculations to elucidate our proposed mechanism after evaluation of the energy profiles of two possible pathways. In this Ag(I) catalysis, the calculations suggest that the amide and alkyne groups of the 3,3-dicarbonyl-2-alkyne intermediates tend to chelate with the Ag(I) catalyst, further inducing a formyl attack at the Ag(I)-π-alkyne moiety to deliver the observed products.

## Introduction

**Scheme 1:**
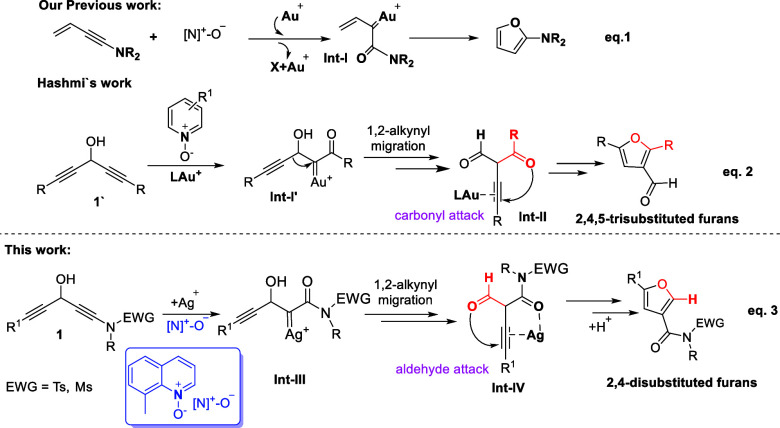
Oxidative Cyclizations by *N*-Oxides

Furan derivatives serve as versatile building blocks in synthetic chemistry. The furan cores are prevalent in numerous natural products and pharmaceuticals,^1^ showing a wide range of biological properties. Numerous efforts have been made to develop new synthetic methods for highly functionalized furans.^2^ In gold catalysis,^3,4^ initial studies of furan synthesis rely heavily on catalytic cycloisomerization of oxygen-containing alkynes^5,6^ such as 1-allenyl *n*-ketones (*n* = 4 and 5),^ ^5a,5b^−5c^ 1-butyn-4-ones,^5d^ pent-4-yn-1-ol,^5e,5f^ 1,4-diyn-3-ols,^5g^ alk-1-ynyl oxiranes,^6a,6b^ 2-(alk-2-yn-1-ylidine)-1,3-diones,^6c^ 2-(1-alkynyl)-2-alken-1-ones,^6d^ 1-(1-alkynyl)cyclopropyl ketone,^6e^ β-alkynylallylic alcohols,^6f^ propargyl vinyl ether,^6g^ and enyne 1,6-diols.^6h^ Furan synthesis using oxygen-free alkynes is highly desirable to expand the scope of synthetic utility. We have recently developed a new furan synthesis from catalytic oxidations of enynamides using pyridine-based N-oxides^7^ (eq 1, Scheme 1); the generation of α-oxo gold carbenes **Int-I** in the mechanism is particularly noteworthy.^8^ Hashmi and co-workers^9a^ employed these alkyne oxidations on 1,4-diyn-3-ols 1̀ to access highly substituted furan derivatives (eq 2). The mechanism of these reactions involves gold carbenes **Int-I′**, followed by a 1,2-alkyne migration. Furthermore, the key 1,3-dicarbonyl-2-alkyne intermediates **Int-II** preferentially undergo a subsequent cyclization to afford 2,4,5-trisubstituted furan derivatives.^9^ Herein, the substituted ketone group (RCO, R = alkyl or aryl) participates in the construction of a furan ring, whereas the aldehyde group of species **Int-II** remains intact. This regioselectivity can be rationalized by the fact that a ketone group (RCO) is a better nucleophile than an aldehyde. To continue our interest in gold-catalyzed reactions of 1,4-diyn-3-ols,^10^ we reveal herein how the introduction of an ynamide provides distinct new chemoselectivity in Hashmi’s 1,3-diynol system using a Ag (I) catalyst. In this approach, furan construction involves the formed aldehyde to yield the 2,4-disubstituted furans, rather than the amide derived from the ynamide. We postulate that the Ag(I) ion chelates with an amide and an alkyne ligand, as depicted by intermediate **Int-IV**, further inducing an aldehyde attack at this Ag-π-alkyne functionality (eq 3). This proposed mechanism is further supported by density functional theory (DFT) calculations in comparison with the energy profiles of Hashmi reactions (eq 2).

## Results and Discussion

We tested the reactions of *N*-(3-hydroxy-5-phenylpenta-1,4-diyn-1-yl)-*N*,4-dimethylbenzenesulfonamide **1a** with 8-methylquinoline *N*-oxide **2a** using various gold and silver catalysts; the results are summarized in Table 1.

**Table 1:**
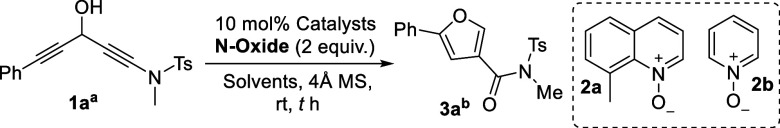
Optimization of Reaction Conditions

entry	catalyst	solvent	time (h)	yield (%)^b^
1	LAuCl/NaBARF	toluene	8	37
2	LAuCl/AgNTf_2_	DCE	8	58
3	IPrAuCl/AgNTf_2_	DCE	9	52
4	(PhO)_3_PAuCl/AgNTf_2_	DCE	8	29
5	PPh_3_AuCl/AgNTf_2_	DCE	7	48
6	LAuCl/AgOTf	DCE	12	73
7	LAuCl/AgSbF_6_	DCE	12	55
8	AuCl_3_	DCE	4	23
9	AgOTf	DCE	6	81
10	AgOTf	DCM	6	83
11	AgSbF_6_	DCM	8	74
12	AgNTf_2_	DCM	7	78
13	AgBF_4_	DCM	6	75
14	CF_3_COOAg	DCM	6	61
15	AgOTf	THF	6	65
16	AgOTf	Toluene	12	63
17	AgOTf	CH_3_CN	7	54
18	AgOTf	dioxane	8	76
19	HOTf	DCM	24	
20	Zn(OTf)_2_	DCM	24	
21	AgOTf	DCM	6	45

a Reaction conditions: **1a** = 0.1 M.

b Product yields are reported after separation from a silica column. L = P(*t*-Bu)_2_(o-biphenyl), IPr = 1,3-bis(2,6-diisopropylphenyl)imidazol-2-ylidine, BARF = tetrakis(3,5-bis(trifluoromethyl)phenyl)borate, DCM = dichloromethane, DCE = 1,2-dichloroethane, THF = tetrahydrofuran, and dioxane = 1,4-dioxane.

Our initial test with P(*t*-Bu)_2_(*o*-biphenyl)AuCl/NaBARF (10 mol %) in toluene (25 °C, 8 h) led to the formation of *N*-methyl-5-phenyl-*N*-tosylfuran-3-carboxamide **3a** in a 37% yield (entry 1). NaBARF was replaced with AgNTf_2_ using P(*t*-Bu)_2_(*o*-biphenyl)AuCl (10 mol %) in DCE (25 °C, 8 h); the yield of desired product **3a** was increased to 58% (entry 2). With IPrAuCl/AgNTf_2_ (10 mol %), the yield of compound **3a** decreased to 52% (entry 3). Other gold phosphine catalysts, including L’AuCl/AgOTf (L’=(PhO)_3_P, PPh_3_), delivered substituted furan **3a** in 29% and 48% yields, respectively (entries 4–5). For P(*t*-Bu)_2_(*o*-biphenyl)AuCl, its AgX (X = OTf and SbF_6_) salts furnished compound **3a** in 73% and 55% yields, respectively (entries 6 and 7). AuCl_3_ (10 mol %) gave the yield of compound **3a** in 23% (entry 8). A test with AgOTf alone gave the best yield, surprisingly up to 83% (entry 9–10). We have tested several silver catalysts including AgSbF_6_, AgNTf_2_, and AgBF_4_, affording our target **3a** in 74%, 78%, and 75% yields, respectively (entries 11–13). CF_3_COOAg was also compatible with this reaction, giving product **3a** in a 61% yield (entry 14). For AgOTf, the yields of compound **3a** in different solvents were as follows (entries 15–18): THF (65%), toluene (63%), CH_3_CN (54%), and 1,4-dioxane (76%). Further, we have checked the Bronsted (HOTf) and Lewis acid (Zn(OTf)_2_) activation for the reaction and consequently failed to catalyze this reaction (entries 19–20). In addition, we examined the reaction with other N-oxides such as pyridine N-oxide (**2b**) instead of **2a** under standard conditions (entry 10) resulting in the desired product **3a** with only a 45% yield (entry 21). The molecular structure of compound **3a** was inferred from X-ray diffraction.^11^

Next, we have prepared the same substrate 1,4-diyn-3-ol **5** as prepared by Hashmi et al.^9a^ to examine the reaction chemoselectivity under our standard condition and 80 °C in DCE over a protracted period. In both of these cases, the reactions only led to recovery of the starting materials (eq 4). Accordingly, formation of **3a** from reactants **1a** and **2a** is not due to the use of a AgOTf catalyst. Diynamides (**1a**) can form key intermediate **H** (vide infra, Scheme 3), in which silver is chelated with one amide and one alkyne group to afford 2,4-disubstituted furan derivatives (**3**), whereas diyne (**5**) fails to form such a chelate complex.
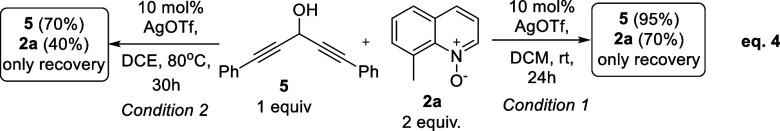
4

Under the optimized condition, a wide range of 1,4-diynamide-3-ols **1** and 8-methylquinoline *N*-oxide **2a** were examined using the AgOTf catalyst (10 mol %) in DCM; the results are summarized in Table 2. We tested the reaction with several 1,4-diynamide-3-ols (**1b–1e)** bearing various alkyl groups at sulfonamides (R = *n-*butyl, isopropyl, cyclopropyl, and cyclohexyl), further rendering the desired furans-3-carboxamides (**3b–3e)** in 69–81% yields. 1,4-Diynamide-3-ols **1f–1h** bearing various aryl groups 4-XC_6_H_4_ (X = H, CH_3_, and Br) at sulfonamides afforded the expected products (**3f–3h)** in 73–79% yields. For the sulfonamide bearing a MeSO_2_ group **1i**, its silver-catalyzed reaction delivered the desired furan **3i** in a 59% yield. 1,4-Diynoxazolidin-2-one-3-ol **1j** worked well to furnish the expected product **3j** in a 45% yield. Our next task is to alter the alkynyl substituents as in 1,4-diynamide-3-ols (**1k–1n)** bearing R^1^ = 4-XC_6_H_4_ (X = Me, Cl, and OMe), and R^1^ = 2-MeC_6_H_4_ effectively providing furan derivatives **3k**–**3n** in 56–83% yields. Alkyl variations at the alkynyl substituents as in 1,4-diynamide-3-ols (**1o–1p**, R^1^ = *n-Bu* and cyclopropyl) were also feasible with this oxidative cyclization to deliver the furan products **3o** and **3p** in 78% and 81% yields, respectively. The presence of a heteroaryl group at the alkyne position (**1q**, R^1^ = thienyl) was also compatible with this catalysis, yielding the desired product **3q** in an 82% yield. We also prepared 1-methylvinyl-containing substrates **1r**, yielding a 2,4-disubstituted furan **3r** in a 61% yield.

**Table 2:**
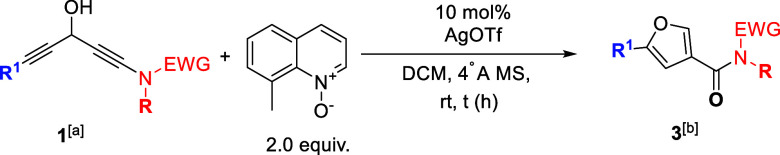

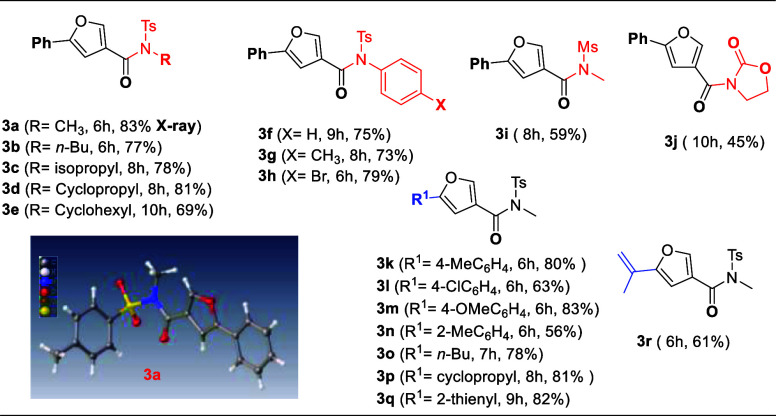
Substrate Scope for the Synthesis of 2,4-Disubstituted Furans

a 1 = 0.1 M.

b Product yields are obtained after purification from a silica column, DCM = dichloromethane.

**Scheme 2:**
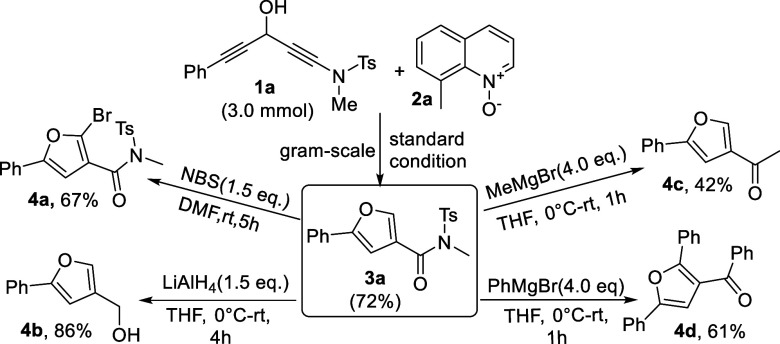
Chemical Functionalizations of **3a**

Chemical functionalizations of one representative compound **3a** are provided in Scheme 2; a gram-scale reaction of **1a** (3.0 mmol) was carried out under standard conditions, delivering the desired product **3a** in a 72% yield. Treatment of furan **3a** with *N*-bromosuccinimide led to formation of 2-bromo-*N*-methyl-5-phenyl-*N*-tosylfuran-3-carboxamide **4a** in a 67% yield. LiAlH_4_ reduction of furan **3a** afforded (5-phenylfuran-3-yl)methanol **4b** in an 86% yield. Treatment of furan **3a** with MeMgBr furnished one single addition product, 1-(5-phenylfuran-3-yl)ethan-1-one **4c**, in a 42% yield. Notably, the use of PhMgBr on furan **3a** enabled 1,3-double addition reactions, affording (2,5-diphenylfuran-3-yl)(phenyl)methanone **4d** in a 61% yield. In this case, we postulate that the first addition occurs at the amide to form a phenylketone intermediate such as species **4c**; the second addition occurs at the furan C(5)-carbon in a Michael-type enone addition before aerobic oxidation.

**Scheme 3:**
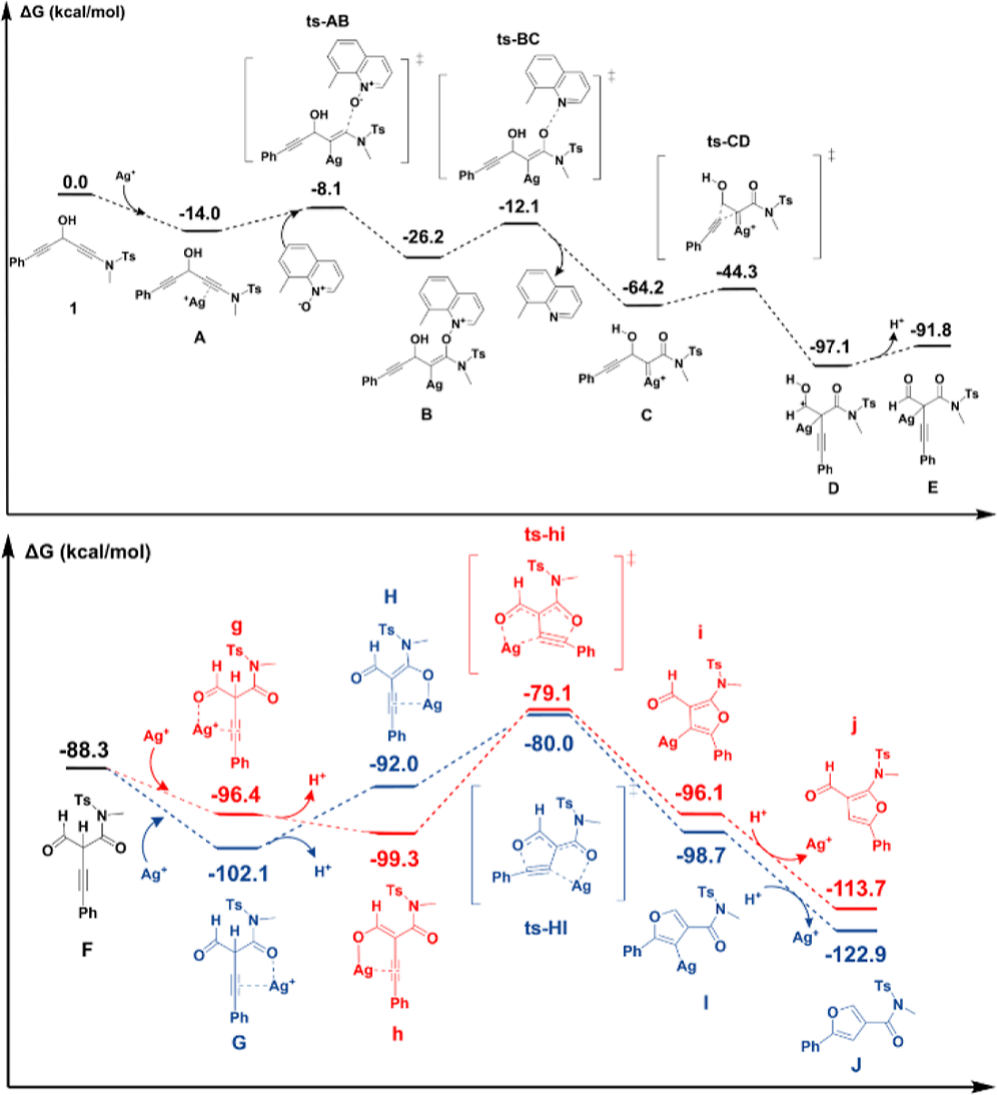
Gibbs Free Energy Profiles for Two Independent Routes

We utilized DFT calculations to elucidate the reaction mechanism of this silver-catalyzed reaction. Scheme 3 presents the Gibbs free energy diagram for the conversion of 1,4-diynamide-3-ol (**1a**) into substituted furans-3-carboxamides (**3a**) using a AgOTf catalyst in DCM. Two independent cyclization pathways were computed for the intramolecular cyclization of key intermediate **F**. Detailed calculation procedures are provided in the Supporting Information. As depicted in Scheme 3, the formation of silver-π alkyne complex **A** from **1a** is exothermic with a Δ*G* of −14.0 kcal/mol. A nucleophilic attack by the *N*-oxide species on the silver-π alkyne generates vinyl silver intermediate **B** with a small activation barrier (Δ*G*^⧧^ = 5.9 kcal/mol). The formation of α-oxo silver carbenes **C** from intermediate **B** proceeds with an activation barrier of Δ*G*^⧧^ = 14.1 kcal/mol. Subsequently, a 1,2-alkyne migration occurs on **C** to form **D**,^9^ with this process presenting a relatively high yet accessible barrier (Δ*G*^⧧^ = 19.9 kcal/mol). Deprotonation of **D** forms **E** with a Δ*G* of −5.3 kcal/mol. Finally, a protodemetalation of species **E** yields the free 1,3-dicarbonyl-2-alkyne species **F**.

There are two possible cyclization routes for intermediate **F**, indicated by the blue and red pathways. **F** can coordinate with Ag^+^ to form either **G** or **g**. Our DFT calculations show that **G** is more stable than **g** by Δ*G* = 5.7 kcal/mol. Subsequently, deprotonation occurs, generating **H** and **h** from **G** and **g**, respectively. The conversion of **G** to **H** is uphill with Δ*G* = 10.1 kcal/mol, whereas the conversion of **g** to **h** is slightly downhill with Δ*G* = −2.9 kcal/mol. Cyclization then occurs, producing **I** and **i** from **H** and **h**, respectively. Finally, demetalation and protonation lead to the formation of **J** and **j**. Our DFT calculations indicate that the blue route is kinetically more favorable (ΔΔG^⧧^ = −0.9 kcal/mol) and thermodynamically more favorable than the red route (ΔΔG = −9.2 kcal/mol), suggesting that **J** (**3a**) is the major product of the reaction, consistent with our experimental results. The change in chemoselectivity observed with our sulfonamide-containing 1,4-diyn-3-ols (**1**) is likely due to the electron-rich effect of sulfonamide amides, which enhances their ability to chelate a Ag(I) ion. As shown in Table 1, the use of Au(I) catalysts can also produce such 2,4-disubstituted furans, although with a lower efficiency.

## Conclusions

In conclusion, we have developed efficient and mild silver-catalyzed oxidative cyclizations of 1,4-diynamide-3-ols (**1**) with 8-methylquinoline *N*-oxide (**2a**), affording 2-substituted furans-4-carboxamides. This silver catalysis serves as a complementary tool to access substituted furans, distinct from those obtained by Hashmi and co-workers. DFT calculations support a reaction mechanism that the amide and alkyne groups of key 1,3-dicarbonyl-2-alkyne intermediates enable to chelate with the Ag(I) catalyst, further inducing an aldehyde attack at the Ag(I)-π-alkyne moiety.

## Experimental Section

### General Information

Unless otherwise noted, all of the reactions for the preparation of the substrates were performed in oven-dried glassware under a nitrogen atmosphere with freshly distilled solvents. The catalytic reactions were performed under a nitrogen atmosphere. DCM, diethyl ether, and toluene were distilled from CaH_2_ under nitrogen. THF was distilled from the Na metal under nitrogen. All other commercial reagents were used without further purification, unless otherwise indicated. 1H NMR and 13C NMR spectra were recorded on Varian 700 and Bruker 400 MHz spectrometers using chloroform-*d* (CDCl_3_) as the internal standard. High-resolution mass spectroscopy (HRMS) data were measured on a JMST100LP4G (JEOL) mass spectrometer or a TOF mass analyzer equipped with an ESI source, JEOL model JMS-T200GC AccuTOF GCx equipped with a field desorption source and a magnetic sector mass analyzer (MStation) equipped with an EI source. Single-crystal X-ray diffraction intensity data were collected on a Bruker X8 APEX diffractometer equipped with a CCD area detector and Mo Kα radiation (λ = 0.71073 Å) at 100 K; all data calculations were performed by using the PC version of the APEX2 program package.

### Standard Procedures for Catalytic Operations

#### Typical Procedure for the Synthesis of *N*-Methyl-5-phenyl-N-tosylfuran-3-carboxamide (**3a**)

To a stirred suspension of AgOTf (3.78 mg, 0.0147 mmol) in DCM (0.5 mL) was fitted a N_2_ balloon. To this suspension was added a DCM (1.0 mL) solution of *N*-(3-hydroxy-5-phenylpenta-1,4-diyn-1-yl)-*N*,4-dimethylbenzenesulfonamide **1a** (50 mg, 0.1473 mmol) and 8-methylquinoline *N*-oxide **2a** (46.90 mg, 0.2946 mmol) at room temperature. The resulting mixture was stirred at room temperature for 6 h. The solution was filtered over a short Celite bed and evaporated under reduced pressure. The residue was purified on a silica gel column using ethyl acetate/hexane (15:75) as the eluent to give compound **3a** as a white solid (43.50 mg, 0.1238 mmol, 83%).

##### *N*-Methyl-5-phenyl-*N*-tosylfuran-3-carboxamide (**3a**)

Purified on silica gel column using ethyl acetate/hexane (15: 85) as the eluent; white solid (43.5 mg, 0.12 mmol, 83%); mp: 115–118 °C; ^1^H NMR (700 MHz, CDCl_3_): δ 7.90 (s, 1H), 7.82 (d, *J* = 8.4 Hz, 2H), 7.63 (d, *J* = 7.7 Hz, 2H), 7.39 (t, *J* = 7.7 Hz, 2H), 7.31 ∼ 7.29 (m, 3H), 6.83 (s, 1H), 3.41 (s, 3H), 2.41 (s, 3H); ^13^C{^1^H} NMR (175 MHz, CDCl_3_): δ 164.6, 154.7, 146.3, 144.9, 135.2, 129.6, 129.4, 128.8, 128.4, 128.3, 124.1, 123.0, 104.9, 35.0, 21.7; HRMS (ESI-TOF) *m*/*z*: [M + Na]^+^ calcd C_19_H_17_NO_4_SNa, 378.0776; found, 378.0778.

##### *N*-Butyl-5-phenyl-*N*-tosylfuran-3-carboxamide (**3b**)

Purified on silica gel column using ethyl acetate/hexane (15: 85) as the eluent; white solid (40 mg, 0.10 mmol, 77%); mp: 75–78 °C; ^1^H NMR (700 MHz, CDCl_3_): δ 7.87 (s, 1H), 7.76 (d, *J* = 8.4 Hz, 2H), 7.61 (d, *J* = 7.7 Hz, 2H), 7.38 (t, *J* = 7.7 Hz, 2H), 7.31–7.27 (m, 3H), 6.78 (s, 1H), 3.84 (t, *J* = 7.7 Hz, 2H), 2.39 (s, 3H), 1.73 (quint, *J* = 7.7 Hz, 2H), 1.37–1.32 (m, 2H), 0.91 (t, *J* = 7.7 Hz, 3H); ^13^C{^1^H} NMR (175 MHz, CDCl_3_): δ 164.7, 154.7, 145.8, 144.6, 136.1, 129.5, 129.4, 128.8, 128.4, 128.3, 124.1, 123.6, 104.9, 47.4, 32.0, 21.6, 19.9, 13.6; HRMS (ESI-TOF) *m*/*z*: [M + Na]^+^ calcd C_22_H_23_NO_4_SNa, 420.1245; found, 420.1245.

##### *N*-Isopropyl-5-phenyl-*N*-tosylfuran-3-carboxamide (**3c**)

Purified on silica gel column using ethyl acetate/hexane (15: 85) as the eluent; white solid (41 mg, 0.11 mmol, 78%); mp: 80–82 °C; ^1^H NMR (700 MHz, CDCl_3_): δ 7.80 (s, 1H), 7.76 (d, *J* = 7.7 Hz, 2H), 7.60 (t, *J* = 7.0 Hz, 2H), 7.38 (t, *J* = 7.7 Hz, 2H), 7.30–7.26 (m, 3H), 6.71 (s, 1H), 4.49–4.45 (m, 1H), 2.39 (s, 3H), 1.50 (d, *J* = 7.0 Hz, 6H); ^13^C{^1^H} NMR (175 MHz, CDCl_3_): δ 165.3, 154.7, 145.9, 144.5, 137.1, 129.51, 129.50, 128.8, 128.34, 128.32, 125.2, 124.1, 104.5, 53.6, 30.3, 21.7, 21.6; HRMS (ESI-TOF) *m*/*z*: [M + Na]^+^ calcd C_21_H_21_NO_4_SNa, 406.1089; found, 406.1090.

##### *N*-Cyclopropyl-5-phenyl-*N*-tosylfuran-3-carboxamide (**3d**)

Purified on silica gel column using ethyl acetate/hexane (15: 85) as the eluent; white solid (42 mg, 0.11 mmol, 81%); mp: 120–125 °C; ^1^H NMR (700 MHz, CDCl_3_): δ 8.00 (s, 1H), 7.91 (d, *J* = 8.4 Hz, 2H), 7.64 (d, *J* = 7.7 Hz, 2H), 7.39 (t, *J* = 7.7 Hz, 2H), 7.32 ∼ 7.23 (m, 3H), 6.93 (s, 1H), 2.88 ∼ 2.85 (m, 1H), 2.41(s, 3H), 0.97 (q, *J* = 7.0 Hz, 2H), 0.83 (q, *J* = 4.2 Hz, 2H); ^13^C{^1^H} NMR (175 MHz, CDCl_3_): δ 165.4, 154.8, 147.3, 144.8, 135.9, 129.53, 129.50, 128.8, 128.7, 128.4, 124.5, 124.1, 104.7, 29.3, 21.6, 10.1; HRMS (ESI-TOF) *m*/*z*: [M + Na]^+^ calcd C_21_H_19_NO_4_SNa, 404.0932; found, 404.0938.

##### *N*-Cyclohexyl-5-phenyl-*N*-tosylfuran-3-carboxamide (**3e**)

Purified on silica gel column using ethyl acetate/hexane (15: 85) as the eluent; yellow oil (36 mg, 0.08 mmol, 69%); ^1^H NMR (700 MHz, CDCl_3_): δ 7.83 (s, 1H), 7.78 (d, *J* = 7.7 Hz, 2H), 7.61 (d, *J* = 7.7 Hz, 2H), 7.38 (t, *J* = 7.7 Hz, 2H), 7.31–7.24 (m, 3H), 6.73 (s, 1H), 3.99–3.96 (m, 1H), 2.39 (s, 3H), 2.12 (q, *J* = 12.6 Hz, 2H), 1.88–1.79 (m, 4H), 1.60–1.57 (m, 1H), 1.23 (q, *J* = 13.3 Hz, 2H), 1.17–1.11 (m, 1H); ^13^C{^1^H} NMR (175 MHz, CDCl_3_): δ 165.6, 154.9, 146.4, 144.3, 137.2, 129.5, 129.4, 128.8, 128.4, 128.3, 125.5, 124.1, 104.5, 61.9, 31.9, 26.6, 25.0, 21.6; HRMS (ESI-TOF) *m*/*z*: [M + Na]^+^ calcd C_24_H_25_NO_4_SNa, 446.1402; found, 446.1401.

##### *N*,5-Diphenyl-*N*-tosylfuran-3-carboxamide (**3f**)

Purified on silica gel column using ethyl acetate/hexane (15: 85) as the eluent; white solid (39 mg, 0.09 mmol, 75%); mp: 122–124 °C; ^1^H NMR (700 MHz, CDCl_3_): δ 7.94 (d, *J* = 7.7 Hz, 2H), 7.55 (t, *J* = 7.7 Hz, 1H), 7.50 (t, *J* = 8.4 Hz, 2H), 7.43–7.42 (m, 2H), 7.37–7.34 (m, 4H), 7.30 (t, *J* = 7.7 Hz, 2H), 7.24–7.22 (m, 1H), 6.56 (s, 1H), 6.46 (s, 1H), 2.44 (s, 3H); ^13^C{^1^H} NMR (175 MHz, CDCl_3_): δ 162.3, 154.0, 146.3, 144.9, 136.4, 135.9, 130.8, 130.4, 129.7, 129.4, 129.3, 129.2, 128.7, 128.2, 123.9, 122.9, 105.5, 21.7; HRMS (ESI-TOF) *m*/*z*: [M + Na]^+^ calcd C_24_H_19_NO_4_SNa, 440.0932; found, 440.0930.

##### 5-Phenyl-*N*-(*p*-tolyl)-*N*-tosylfuran-3-carboxamide (**3g**)

Purified on silica gel column using ethyl acetate/hexane: (15: 85) as the eluent; white solid (38 mg, 0.09 mmol, 73%); mp: 131–135 °C; ^1^H NMR (700 MHz, CDCl_3_): δ 7.94 (d, *J* = 7.7 Hz, 2H), 7.44 (d, *J* = 7.7 Hz, 2H), 7.34 ∼ 7.29 (m, 6H), 7.24–7.23 (m, 3H), 6.56 (s, 1H), 6.50 (s, 1H), 2.44 (s, 6H); ^13^C{^1^H} NMR (175 MHz, CDCl_3_): δ 162.4, 153.9, 146.1, 144.9, 140.9, 136.0, 133.7, 130.5, 130.4, 129.4, 129.3, 128.7, 128.2, 123.9, 122.9, 105.8, 21.7, 21.4; one carbon merge with other peak; HRMS (ESI-TOF) *m*/*z*: [M + Na]^+^ calcd C_25_H_21_NO_4_SNa, 454.1089; found, 454.1084.

##### *N*-(4-Bromophenyl)-5-phenyl-*N*-tosylfuran-3-carboxamide (**3h**)

Purified on silica gel column using ethyl acetate/hexane (15: 85) as the eluent; brown solid (41 mg, 0.08 mmol, 79%); mp: 160–163 °C; ^1^H NMR (700 MHz, CDCl_3_): δ 7.89 (d, *J* = 7.0 Hz, 2H), 7.61 (d, *J* = 7.0 Hz, 2H), 7.44 (d, *J* = 7.0 Hz, 2H), 7.34–7.30 (m, 4H), 7.25 (t, *J* = 7.7 Hz, 1H), 7.20 (d, *J* = 7.0 Hz, 2H), 6.72 (s, 1H), 6.49 (s, 1H), 2.43 (s, 3H); ^13^C{^1^H} NMR (175 MHz, CDCl_3_): δ 162.0, 154.4, 146.2, 145.2, 135.53, 135.50, 133.0, 132.2, 129.44, 129.40, 129.1, 128.8, 128.4, 124.7, 123.9, 122.8, 105.3, 21.7; HRMS (ESI-TOF) *m*/*z*: [M + Na]^+^ calcd C_24_H_18_BrNO_4_SNa, 518.0037; found, 518.0038.

##### *N*-Methyl-*N*-(methylsulfonyl)-5-phenylfuran-3-carboxamide (**3i**)

Purified on silica gel column using ethyl acetate/hexane (15: 85) as the eluent; yellow oil (31 mg, 0.11 mmol, 59%); ^1^H NMR (700 MHz, CDCl_3_): δ 7.95 (s, 1H), 7.66 (d, *J* = 7.7 Hz, 2H), 7.40 (t, *J* = 7.7 Hz, 2H), 7.33 (t, *J* = 7.7 Hz, 1H), 6.90 (s, 1H), 3.46 (s, 3H), 3.35 (s, 3H); ^13^C{^1^H} NMR (175 MHz, CDCl_3_): δ 165.2, 155.2, 145.9, 129.2, 128.9, 128.6, 124.2, 122.5, 104.6, 41.4, 34.8; HRMS (ESI-TOF) *m*/*z*: [M + Na]^+^ calcd C_13_H_13_NO_4_SNa, 302.0463; found, 302.0460.

##### 3-(5-Phenylfuran-3-carbonyl)oxazolidin-2-one (**3j**)

Purified on silica gel column using ethyl acetate/hexane (15: 85) as the eluent; yellow solid (24 mg, 0.09 mmol, 45%); mp: 142–145 °C; ^1^H NMR (700 MHz, CDCl_3_): δ 8.32 (s, 1H), 7.66 (d, *J* = 7.7 Hz, 2H), 7.38 (t, *J* = 7.0 Hz, 2H), 7.29 (t, *J* = 7.0 Hz, 1H), 7.07 (s, 1H), 4.47 (t, *J* = 7.7 Hz, 2H), 4.16 (t, *J* = 7.7 Hz, 2H); ^13^C{^1^H} NMR (175 MHz, CDCl_3_): δ 162.1, 153.9, 153.0, 148.5, 129.7, 128.7, 128.1, 124.1, 121.0, 105.9, 62.4, 43.9; HRMS (ESI-TOF) *m*/*z*: [M + Na]^+^ calcd C_14_H_11_NO_4_Na, 280.0586; found, 280.0582.

##### *N*-Methyl-5-(*p*-tolyl)-*N*-tosylfuran-3-carboxamide (**3k**)

Purified on silica gel column using ethyl acetate/hexane (15: 85) as the eluent; white solid (42 mg, 0.11 mmol, 80%); mp: 117–120 °C; ^1^H NMR (700 MHz, CDCl_3_): δ 7.87 (s, 1H), 7.82 (d, *J* = 7.0 Hz, 2H), 7.51 (d, *J* = 7.0 Hz, 2H), 7.30 (d, *J* = 7.7 Hz, 2H), 7.18 (d, *J* = 7.0 Hz, 2H), 6.76 (s, 1H), 3.40 (s, 3H), 2.41 (s, 3H), 2.35 (s, 3H); ^13^C{^1^H} NMR (175 MHz, CDCl_3_): δ 164.7, 154.9, 145.9, 144.8, 138.4, 135.3, 129.6, 129.5, 128.6, 129.5, 128.3, 126.7, 124.1, 123.0, 104.2, 35.0, 21.6, 21.3; HRMS (ESI-TOF) *m*/*z*: [M – H]^−^ calcd C_20_H_18_NO_4_S, 368.0956; found, 368.0947.

##### 5-(4-Chlorophenyl)-*N*-methyl-*N*-tosylfuran-3-carboxamide (**3l**)

Purified on silica gel column using ethyl acetate/hexane (15: 85) as the eluent; white solid (33 mg, 0.08 mmol, 63%); mp: 135–137 °C; ^1^H NMR (700 MHz, CDCl_3_): δ 7.89 (s, 1H), 7.80 (d, *J* = 4.9 Hz, 2H), 7.55 (d, *J* = 5.6 Hz, 2H), 7.36–7.31 (m, 4H), 6.83 (s, 1H), 3.38 (s, 3H), 2.41 (s, 3H); ^13^C{^1^H} NMR (175 MHz, CDCl_3_): δ 164.5, 153.6, 146.4, 144.9, 135.2, 134.2, 129.6, 129.1, 128.3, 127.9, 125.3, 123.2, 105.4, 34.9, 21.6; HRMS (ESI-TOF) *m*/*z*: [M + Na]^+^ calcd C_19_H_16_ClNO_4_SNa, 412.0386; found, 412.0383.

##### 5-(4-Methoxyphenyl)-*N*-methyl-*N*-tosylfuran-3-carboxamide (**3m**)

Purified on silica gel column using ethyl acetate/hexane (20: 80) as the eluent; white solid (43.2 mg, 0.11 mmol, 83%); mp: 125–128 °C; ^1^H NMR (400 MHz, CDCl_3_): δ 7.84–7.81 (m, 3H), 7.55–7.52 (m, 2H), 7.30 (d, *J* = 8.0 Hz, 2H), 6.91–6.89 (m, 2H), 6.68 (s, 1H), 3.81 (s, 3H), 3.39 (s, 3H), 2.40 (s, 3H); ^13^C{^1^H} NMR (100 MHz, CDCl_3_): δ 164.7, 159.7, 154.8, 145.6, 144.8, 135.3, 129.5, 128.3, 125.6, 122.9, 122.3, 114.2, 103.2, 55.3, 34.9, 21.5; HRMS-ESI^+^*m*/*z*: [M + Na]^+^ calcd C_20_H_19_NO_5_SNa, 408.0882; found, 408.0883.

##### *N*-Methyl-5-(*o*-tolyl)-*N*-tosylfuran-3-carboxamide (**3n**)

Purified on silica gel column using ethyl acetate/hexane (15: 85) as the eluent; yellow oil (29.5 mg, 0.08 mmol, 56%); ^1^H NMR (700 MHz, CDCl_3_): δ 7.92 (s, 1H), 7.82 (d, *J* = 8.4 Hz, 2H), 7.62–7.61 (m, 1H), 7.30 (d, *J* = 8.4 Hz, 2H), 7.24–7.22 (m, 3H), 6.71 (s, 1H), 3.41 (s, 3H), 2.43 (s, 3H), 2.40 (s, 3H); ^13^C{^1^H} NMR (175 MHz, CDCl_3_): δ 164.7, 154.2, 145.8, 144.8, 135.3, 135.0, 131.2, 129.6, 128.7, 128.4, 128.3, 127.2, 126.1, 122.7, 108.5, 34.9, 21.67, 21.62; HRMS-ESI^+^*m*/*z*: [M + Na]^+^ calcd C_20_H_19_NO_4_SNa, 392.0932; found, 392.0934.

##### 5-Butyl-*N*-methyl-*N*-tosylfuran-3-carboxamide (**3o**)

Purified on silica gel column using ethyl acetate/hexane (15: 85) as the eluent; yellow oil (41 mg, 0.12 mmol, 78%); ^1^H NMR (700 MHz, CDCl_3_): δ 7.81 (d, *J* = 7.7 Hz, 2H), 7.73 (s, 1H), 7.29 (d, *J* = 7.0 Hz, 2H), 6.20 (s, 1H), 3.36 (s, 3H), 2.57 (t, *J* = 7.0 Hz, 2H), 2.40 (s, 3H), 1.58–1.56 (m, 2H), 1.34–1.31 (m, 2H), 0.89 (t, *J* = 7.0 Hz, 3H); ^13^C{^1^H} NMR (175 MHz, CDCl_3_): δ 164.9, 157.6, 145.6, 144.7, 135.5, 129.5, 128.6, 145.6, 144.7, 135.5, 135.5, 129.5, 128.3, 121.7, 105.3, 34.9, 29.7, 27.3, 22.1, 21.6, 13.7; HRMS (ESI-TOF) *m*/*z*: [M + Na]^+^ calcd C_17_H_21_NO_4_SNa, 358.1089; found, 358.1089.

##### 5-Cyclopropyl-*N*-methyl-*N*-tosylfuran-3-carboxamide (**3p**)

Purified on silica gel column using ethyl acetate/hexane (15: 85) as the eluent; yellow oil (43 mg, 0.13 mmol, 81%); ^1^H NMR (700 MHz, CDCl_3_): δ 7.80 (d, *J* = 7.0 Hz, 2H), 7.67 (s, 1H), 7.28 (d, *J* = 7.7 Hz, 2H), 6.16 (s, 1H), 3.35 (s, 3H), 2.40 (s, 3H), 1.83 (br s, 1H), 0.88–0.87 (m, 2H), 0.74 (s, 2H); ^13^C{^1^H} NMR (175 MHz, CDCl_3_): δ 164.8, 158.5, 145.2, 144.7, 135.4, 129.5, 128.3, 121.8, 103.9, 34.9, 21.6, 8.4, 6.8; HRMS (ESI-TOF) *m*/*z*: [M + Na]^+^ calcd C_16_H_17_NO_4_SNa, 342.0776; found, 342.0774.

##### *N*-Methyl-5-(thiophen-2-yl)-*N*-tosylfuran-3-carboxamide (**3q**)

Purified on silica gel column using ethyl acetate/hexane (15: 85) as the eluent; yellow oil (43 mg, 0.12 mmol, 82%); ^1^H NMR (700 MHz, CDCl_3_): δ 7.81 (t, *J* = 8.4 Hz, 3H), 7.31–7.27 (m, 4H), 7.03 (t, *J* = 4.2 Hz, 1H), 6.66 (s, 1H), 3.38 (s, 3H), 2.41 (s, 3H); ^13^C{^1^H} NMR (175 MHz, CDCl_3_): δ 164.4, 150.2, 145.7, 144.9, 135.3, 131.9, 129.6, 128.3, 127.8, 125.5, 124.1, 123.1, 104.7, 34.9, 21.6; HRMS (ESI-TOF) *m*/*z*: [M + Na]^+^ calcd C_17_H_15_NO_4_S_2_Na, 384.0340; found, 384.0347.

##### *N*-Methyl-5-(prop-1-en-2-yl)-*N*-tosylfuran-3-carboxamide (**3r**)

Purified on silica gel column using ethyl acetate/hexane (15: 85) as the eluent; yellow oil (32 mg, 0.10 mmol, 61%); ^1^H NMR (700 MHz, CDCl_3_): δ 7.80 (t, *J* = 7.7 Hz, 3H), 7.29 (d, *J* = 7.7 Hz, 2H), 6.46 (s, 1H), 5.51 (s, 1H), 5.03 (s, 1H), 3.36 (s, 3H), 2.41 (s, 3H), 1.97 (s, 3H); ^13^C{^1^H} NMR (175 MHz, CDCl_3_): δ 164.6, 155.7, 146.3, 144.8, 135.3, 131.8, 129.6, 128.3, 122.6, 112.1, 106.0, 34.9, 21.6, 19.1; HRMS (ESI-TOF) *m*/*z*: [M + Na]^+^ calcd C_16_H_17_NO_4_SNa, 342.0776; found, 342.0776.

## Chemical Functionalizations of **3a**

### Gram-Scale Reaction

To a stirred suspension of AgOTf (77 mg, 0.3 mmol) in DCM (10 mL) was fitted a N_2_ balloon. To this suspension was added a DCM (15 mL) solution of *N*-(3-hydroxy-5-phenylpenta-1,4-diyn-1-yl)-*N*,4-dimethylbenzenesulfonamide **1a** (1.02 g, 3 mmol) and 8-methylquinoline *N*-oxide **2a** (955 mg, 6 mmol) at room temperature. The resulting mixture was stirred at room temperature for 8 h. The solution was filtered over a short Celite bed and evaporated under reduced pressure. The residue was purified on a silica gel column using ethyl acetate/hexane (15:75) as the eluent to give compound **3a** as a white solid (760 mg, 2.2 mmol, 72%).

### Typical Procedure for the Synthesis of 2-Bromo-*N*-methyl-5-phenyl-*N*-tosylfuran-3-carboxamide (**4a**)

To a stirred solution of *N*-methyl-5-phenyl-*N*-tosylfuran-3-carboxamide **3a** (50.0 mg, 0.14 mmol) in DMF (2.0 mL) was added NBS (37.56 mg, 0.21 mmol) at room temperature. The resulting mixture was stirred for 5 h at room temperature. The reaction mixture was quenched with water (3.0 mL), and the solution was then extracted with ethyl acetate (5.0 mL) three times. The organic phase was washed with brine, dried with MgSO_4_, and concentrated under reduced pressure. The residue was purified on a silica column using ethyl acetate/hexane (5:95) as the eluent to give compound **4a** as a yellow oil (41 mg, 0.09 mmol, 67%).

#### 2-Bromo-*N*-methyl-5-phenyl-*N*-tosylfuran-3-carboxamide (**4a**)

Purified on silica gel column using ethyl acetate/hexane (10: 90) as the eluent; yellow oil (41 mg, 0.09 mmol, 67%); ^1^H NMR (700 MHz, CDCl_3_): δ 7.81 (d, *J* = 8.4 Hz, 2H), 7.59 (d, *J* = 7.7 Hz, 2H), 7.38 (t, *J* = 7.7 Hz, 2H), 7.31 (d, *J* = 7.7 Hz, 3H), 6.77 (s, 1H), 3.33 (s, 3H), 2.42 (s, 3H); ^13^C{^1^H} NMR (175 MHz, CDCl_3_): δ 164.1, 155.7, 145.1, 134.9, 129.7, 128.9, 128.7, 128.7, 128.6, 128.3, 125.3, 123.8, 122.2, 107.2, 34.5, 21.6; HRMS (ESI-TOF) *m*/*z*: [M + Na]^+^ calcd C_19_H_16_BrNO_4_SNa, 455.9881; found, 455.9884.

### Typical Procedure for the Synthesis of (5-Phenylfuran-3-yl)methanol (**4b**)

To a stirred solution of *N*-methyl-5-phenyl-*N*-tosylfuran-3-carboxamide **3a** (50.0 mg, 0.14 mmol) in dry THF (2.0 mL) was added LAH (1 M in THF, 0.21 mL, 0.21 mmol) at 0 °C. The resulting mixture was stirred for 4 h at room temperature. The reaction mixture was quenched with a saturated solution of ammonium chloride (3.0 mL), and the solution was then extracted with ethyl acetate (5.0 mL) three times. The organic phase was washed with brine, dried with MgSO_4_, and concentrated under reduced pressure. The residue was purified on a silica column using ethyl acetate/hexane (30:70) as the eluent to give compound **4b** as a yellow oil (21 mg, 0.12 mmol, 86%).

#### (5-Phenylfuran-3-yl)methanol (**4b**)

Purified on silica gel column using ethyl acetate/hexane (30: 70) as the eluent; yellow oil (21 mg, 0.12 mmol, 86%); ^1^H NMR (700 MHz, CDCl_3_): δ 7.63 (d, *J* = 7.7 Hz, 2H), 7.43 (s, 1H), 7.36 (t, *J* = 7.7 Hz, 2H), 7.25 (t, *J* = 7.7 Hz, 1H), 6.68 (s, 1H), 4.57 (s, 2H); ^13^C{^1^H} NMR (175 MHz, CDCl_3_): δ 154.8, 139.3, 130.6, 128.7, 127.5, 127.2, 123.8, 105.0, 56.8; HRFD^+^ calcd for C_11_H_10_O2 [M]^+^, 174.0681; found, 174.0680.

### Typical Procedure for the Synthesis of 1-(5-Phenylfuran-3-yl)ethan-1-one (**4c**)

To a stirred solution of *N*-methyl-5-phenyl-*N*-tosylfuran-3-carboxamide **3a** (50.0 mg, 0.14 mmol) in THF (2.0 mL) was added MeMgBr (3 M in diethyl ether, 0.19 mL, 0.56 mmol) at 0 °C. The resulting mixture was stirred for 1 h at room temperature. The solution was quenched with a saturated solution of ammonium chloride (3.0 mL) at 0 °C, and the solution was then extracted with ethyl acetate (5.0 mL) three times. The organic phase was washed with brine, dried with MgSO_4_, and concentrated under reduced pressure. The residue was purified on a silica column using ethyl acetate/hexane (03:97) as the eluent to give compound **4c** as a yellow oil (11 mg, 0.059 mmol, 42%).

#### 1-(5-Phenylfuran-3-yl)ethan-1-one (**4c**)

Purified on silica gel column using ethyl acetate/hexane (5:95) as the eluent; yellow oil (11 mg, 0.06 mmol, 42%); ^1^H NMR (700 MHz, CDCl_3_): δ 8.01 (s, 1H), 7.66 (d, *J* = 7.7 Hz, 2H), 7.39 (t, *J* = 7.7 Hz, 2H), 7.30 (t, *J* = 7.7 Hz, 1H), 6.97 (s, 1H), 2.45 (s, 3H); ^13^C{^1^H} NMR (175 MHz, CDCl_3_): δ 192.5, 155.8, 146.7, 129.8, 129.7, 128.8, 128.3, 124.1, 103.1, 27.7; HRMS (ESI-TOF) *m*/*z*: [M + Na]^+^ calcd C_12_H_10_O_2_Na, 209.0578; found, 209.0584.

### Typical Procedure for the Synthesis of (2,5-Diphenylfuran-3-yl)(phenyl)methanone (**4d**)

To a stirred solution of *N*-methyl-5-phenyl-*N*-tosylfuran-3-carboxamide **3a** (50.0 mg, 0.14 mmol) in THF (2.0 mL) was added PhMgBr (1 M in THF, 0.56 mL, 0.56 mmol) at 0 °C. The resulting mixture was stirred for 1 h at room temperature. The solution was quenched with a saturated solution of ammonium chloride (3.0 mL) at 0 °C, and the solution was then extracted with ethyl acetate (5.0 mL) three times. The organic phase was washed with brine, dried with MgSO_4_, and concentrated under reduced pressure. The residue was purified on a silica column using ethyl acetate/hexane (03:97) as the eluent to give compound **4d** as a yellow oil (28 mg, 0.086 mmol, 61%).

#### (2,5-Diphenylfuran-3-yl)(phenyl)methanone (**4d**)

Purified on silica gel column using ethyl acetate/hexane (3: 97) as the eluent; yellow oil (28 mg, 0.09 mmol, 61%); ^1^H NMR (700 MHz, CDCl_3_): δ 7.87 (d, *J* = 7.7 Hz, 2H), 7.77–7.74 (m, 4H), 7.51 (t, *J* = 7.7 Hz, 1H), 7.43–7.38 (m, 4H), 7.32–7.29 (m, 4H), 6.92 (s, 1H); ^13^C{^1^H} NMR (175 MHz, CDCl_3_): δ 191.8, 154.9, 152.5, 137.9, 132.9, 129.76, 129.74, 129.70, 129.0, 128.8, 128.37, 128.35, 128.2, 127.4, 124.1, 122.8, 108.7; HRMS (ESI-TOF) *m*/*z*: [M + Na]^+^ calcd C_23_H_16_O_2_Na, 347.1048; found, 347.1050.

## Data Availability

The data underlying this study are available in the published article and its online Supporting Information.

## Supporting Information Available

Scheme of substrate preparation, single-crystal X-ray data, and ^1^H NMR and ^13^C NMR spectra for representative compounds (PDF)
