# Supplementary material for: Ag(I)-Catalyzed Oxidative Cyclization of 1,4-Diynamide-3-ols with N-Oxide for Divergent Synthesis of 2-Substituted Furan-4-carboxamide Derivatives
Source: J Org Chem. 2024 Oct 22;89(21):15924–30. doi: 10.1021/acs.joc.4c02096 (PMC11536374; doi:10.1021/acs.joc.4c02096)

Supporting Information

Ag(I)-Catalyzed Oxidative Cyclization of 1,4-Diynamide-3-ols with *N*-oxide for Divergent Synthesis of 2-Substituted Furan-*4*-Carboxamide Derivatives.

Akshay Subhash Narode,[a] Debashis Barik,[a] Ping-Hsun Kuo[b], Mu-Jeng Cheng[b]* and Rai-Shung Liu[a]*

[a]Department of Chemistry, National Tsing-Hua University, Hsinchu Taiwan, ROC--------------------e-mail: rsliu@mx.nthu.edu.tw

[b]Department of Chemistry, National Cheng Kung University, Tainan, Taiwan, ROC---------------------e-mail: mjcheng@mail.ncku.edu.tw

Contents:

1. Representative Synthetic Procedures: --S2

2. Standard procedures for catalytic operations: --S4

3. Chemical functionalization (3a) ---S4

4. Spectral data for key compounds: ---S6

5. Computational Details ---S14

6. Figure S1: Gibbs free energy profiles ---S16

7. X-ray crystallographic data of 3a--S16

8.^{1}H and ^{13}C spectra of key compounds: --S23

1. Representative Synthetic Procedures:

(a) General procedure:

Unless otherwise noted, all the reactions for the preparation of the substrates were performed in oven-dried glassware under nitrogen atmosphere with freshly distilled solvents. The catalytic reactions were performed under nitrogen atmosphere. DCM, diethyl ether and toluene were distilled from CaH_2 under nitrogen. THF was distilled from Na metal under nitrogen. All other commercial reagents were used without further purification, unless otherwise indicated. 1H NMR and 13C NMR spectra were recorded on a Varian 700 and 500 MHz, Bruker 400 MHz spectrometers using chloroform-*d* ($CDCl_3$) as the internal standard. High-resolution mass spectral analysis (HRMS) data were measured on JMST100LP4G (JEOL) mass spectrometer or a TOF mass analyzer equipped with the ESI source, JEOL Model: JMS-T200GC AccuTOF GCx equipped with FD (field desorption) source and Magnetic Sector Mass Analyzer (MStation) equipped with the EI source. Single-crystal X-ray diffraction intensity data were collected on a Bruker X8 APEX diffractometer equipped with a CCD area detector and Mo Kα radiation (λ = 0.71073 Å) at 100 K; all data calculations were performed by using the PC version of the APEX2 program package.

(b) General synthetic process for *N*-(3-hydroxy-5-phenylpenta-1,4-diyn-1-yl)-*N*,4-dimethylbenzenesulfonamide synthesis (1a):

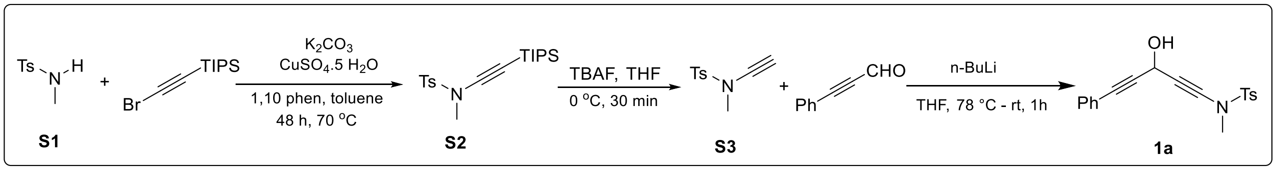

To a mixture of **S1** (3.0 g, 16.20 mmol), K_2CO_3 (4.48 g, 32.39 mmol), $CuSO_4.5H_2O$ (404 mg, 1.62 mmol), and 1,10-phenanthroline (584 mg, 3.24 mmol) in the reaction vial was added a solution of a respective (bromoethynyl)triisopropylsilane (6.35 g, 24.29 mmol) in 30 mL toluene. The reaction mixture was capped and heated in an oil bath at 70°C for 48 h while being monitored with TLC analysis. Upon completion, the reaction mixture was cooled to room temperature and diluted with EA and filtered through celite, and the filtrate was concentrated under vacuum. The crude products were purified by flash column chromatography using silica gel (EA/Hexane= 2:98, v/v) to afford the desired product **S2** as a white solid (4.70 g, 12.86 mmol, 79 %).

To a solution of *N*,*4*-dimethyl-*N*-((triisopropylsilyl)ethynyl)benzenesulfonamide **S2** (2.0 g, 5.47 mmol) in THF (20 mL) was added *n*-tetrabutyl ammonium fluoride (1.0 M in THF, 8.21 mL, 8.21 mmol) at 0 °C, and the resulting mixture was stirred at 0 °C for 10 mins. Then reaction mixture was quenched with water (20 ml) and extracted with ethyl acetate (3x 30 mL), Organic layer was dried over $MgSO_4$ and concentrated under reduced pressure. The crude product was purified by flash column chromatography using silica gel (ethyl acetate: hexane = 3:97, v/v) to afford the desired product **S3** as a white solid (1.00 g, 4.78 mmol, 87 %).

To a solution of **S3** (500 mg, 2.39 mmol) in anhydrous THF (10 ml) cooled to -78 ℃ was added n-BuLi (1.6M in Hexane, 1.79 ml, 2.87mmol). The resulting mixture was stirred at same temperature for 30 min period before the addition of 3-phenylpropiolaldehyde (373mg, 2.87mmol). The reaction mixture was warm up to room temperature and stirred for 30 min. Reaction mixture was quenched with water (20 ml) and extracted with ethyl acetate (3x 20 mL), Organic layer was dried over $MgSO_4$ and concentrated under reduced pressure. The crude product was purified by flash column chromatography using silica gel (ethyl acetate: hexane = 30:70, v/v) to afford the desired product **1a** as a yellow oil (690 mg, 2.03 mmol, 85 %).

All other 1,4-diynamide-3-ols **1b-1p** were synthesized following the same procedure as of **1a.**

(c) General synthetic procedure for Preparation of 8-methylquinoline *N*-oxide (2a):

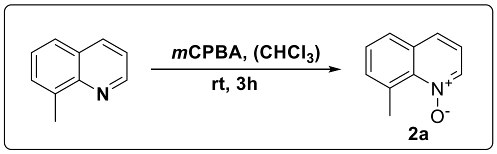

To a stirred solution of 8-methylquinoline (1.00 g, 6.98 mmol) in 20ml of chloroform was treated with *meta*-chloroperbenzoic acid (2.67 g, 7.57 mmol). The mixture was stirred for 3h at room temperature. Subsequently saturated $NaHCO_3$ (25 mL) and 2M NaOH (25mL) were added and the mixture was extracted with DCM. The combined organic layers were dried over $MgSO_4$ and concentrated under reduced pressure yielding the desired *N*-oxide **2a** (767 mg, 4.81 mmol, 69%) as a yellow solid which was used in the following step without further purification. The analytical data agreements with the reported literature.[s1]

References:

s1) L. Bering, A. P. Antonchick, *Org. Lett.* **2015**, *17*, 3134−3137.

2. Standard procedures for catalytic operations.

Typical procedure for the synthesis of N-methyl-5-phenyl-N-tosylfuran-3-carboxamide (3a):

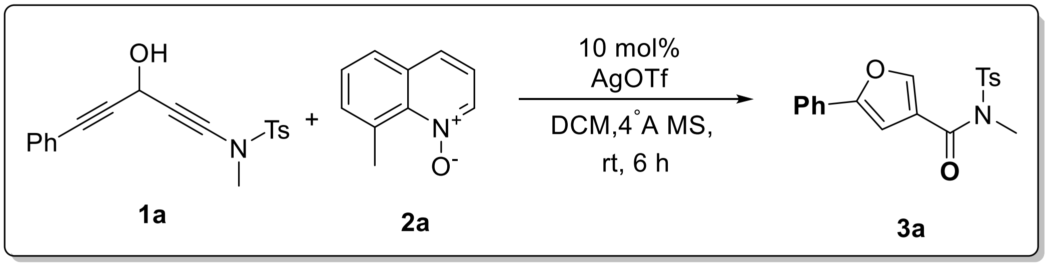

To a stirred suspension of AgOTf (3.78 mg, 0.0147 mmol) in DCM (0.5 mL) was fitted with a N_2 balloon. To this suspension was added a DCM (1.0 mL) solution of *N*-(3-hydroxy-5-phenylpenta-1,4-diyn-1-yl)-*N*,4-dimethylbenzenesulfonamide **1a** (50 mg, 0.1473 mmol) and 8-methylquinoline *N*-oxide **2a** (46.90 mg, 0.2946 mmol) at room temperature. The resulting mixture was stirred at room temperature for 6 h. The solution was filtered over a short celite bed and evaporated under reduced pressure. The residue was purified on a silica gel column using ethyl acetate/hexane (15:85) as the eluent to give compound **3a** as white solid (43.50 mg, 0.1238 mmol, 83%).

(3) Chemical functionalizations of 3a:

(a) Typical procedure for the synthesis of 2-bromo-*N*-methyl-5-phenyl-*N*-tosylfuran-3-carboxamide (**4a**):

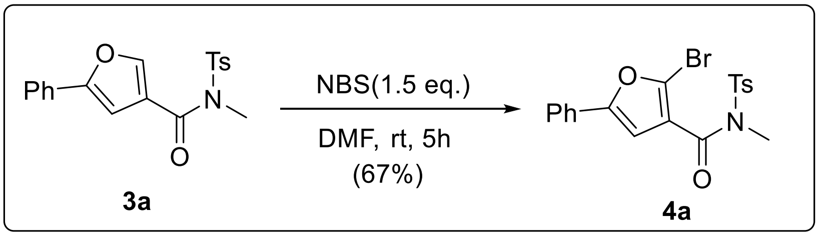

To a stirred solution of *N*-methyl-5-phenyl-*N*-tosylfuran-3-carboxamide **3a** (50 mg, 0.14mmol) in DMF (2.0 mL) was added NBS (37.56 mg, 0.21 mmol) at room temperature. The resulting mixture was stirred for 5h at room temperature. The reaction mixture was quenched with water (3.0 mL) and the solution was then extracted with ethyl acetate (5.0 mL) three times. Organic phase was washed with brine, dried with $MgSO_4$ and concentrated under reduced pressure. The residue was

purified on a silica column using ethyl acetate/hexane (5:95) as the eluent to give compound **4a** as Yellow oil (41 mg, 0.09 mmol, 67%).

(b) Typical procedure for the synthesis of (5-phenylfuran-3-yl)methanol (**4b**):

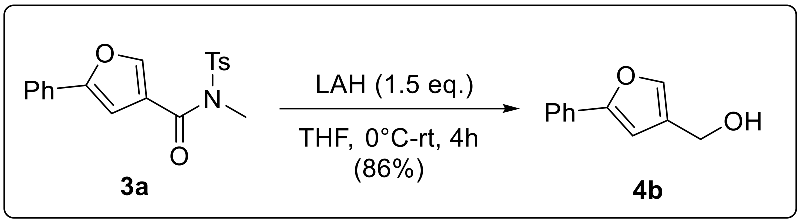

To a stirred solution of *N*-methyl-5-phenyl-*N*-tosylfuran-3-carboxamide **3a** (50 mg, 0.14mmol) in Dry THF (2.0 mL) was added LAH (1M in THF, 0.21 ml, 0.21 mmol) at 0 °C. The resulting mixture was stirred for 4h at room temperature. The reaction mixture was quenched with saturated solution of ammonium chloride (3.0 mL) and the solution was then extracted with ethyl acetate (5.0 mL) three times. Organic phase was washed with brine, dried with $MgSO_4$ and concentrated under reduced pressure. The residue was purified on a silica column using ethyl acetate/hexane (30:70) as the eluent to give compound **4b** as Yellow oil (21 mg, 0.12 mmol, 86%).

(c) Typical procedure for the synthesis of 1-(5-phenylfuran-3-yl)ethan-1-one (**4c**):

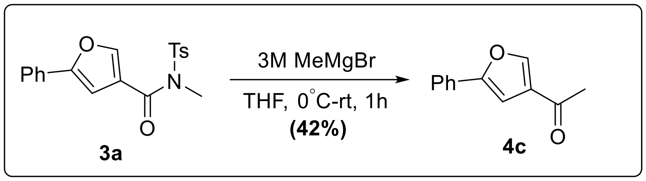

To a stirred solution of *N*-methyl-5-phenyl-*N*-tosylfuran-3-carboxamide **3a** (50 mg, 0.14 mmol) in THF (2.0 mL) was added MeMgBr (3M in diethyl ether, 0.19 mL, 0.56 mmol) at 0 °C. The resulting mixture was stirred for 1h at room temperature. The solution was quenched with a saturated solution of ammonium chloride (3.0 mL) at 0 °C; and the solution was then extracted with ethyl acetate (5.0 mL) three times. Organic phase was washed with brine, dried with $MgSO_4$ and concentrated under reduced pressure. The residue was purified on a silica column using ethyl acetate/hexane (03:97) as the eluent to give compound **4c** as white solid (11 mg, 0.059 mmol, 42%).

(d) Typical procedure for the synthesis of (2,5-diphenylfuran-3-yl)(phenyl)methanone (**4d**):

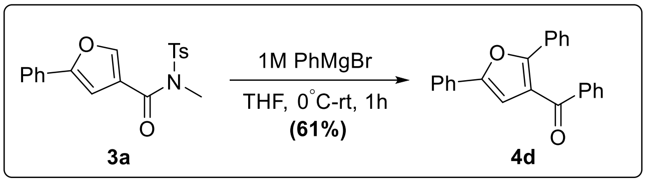

To a stirred solution of *N*-methyl-5-phenyl-*N*-tosylfuran-3-carboxamide **3a** (50mg, 0.14mmol) in THF (2.0 mL) was added PhMgBr (1M in THF, 0.56 mL, 0.56 mmol) at 0 °C. The resulting mixture was stirred for 1h at room temperature. The solution was quenched with a saturated solution of ammonium chloride (3.0 mL) at 0 °C; and the solution was then extracted with ethyl acetate (5.0 mL) three times. Organic phase was washed with brine, dried with $MgSO_4$ and concentrated under reduced pressure. The residue was purified on a silica column using ethyl acetate/hexane (03:97) as the eluent to give compound **4d** as yellow oil (28 mg, 0.086 mmol, 61%).

(4) Spectral data of key compounds:

Spectral data of *N*-(3-hydroxy-5-phenylpenta-1,4-diyn-1-yl)-*N*,4-dimethylbenzenesulfonamide (1a):

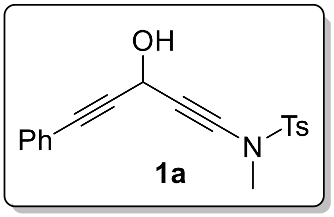

Compound **1a** was purified on silica gel column using ethyl acetate/hexane: (30: 70) as the eluent; Yellow oil (625 mg, 1.84 mmol, 77 %); ^{1}H NMR (400 MHz, $CDCl_3$): δ 7.81 ~ 7.79 (m, 2H), 7.46 ~ 7.43 (m, 2H), 7.33 ~ 7.27 (m, 5H), 5.42 (d, *J* = 6.4 Hz, 1H), 3.07 (s, 3H), 2.58 (d, *J* = 6.8 Hz, 1H), 2.39 (s, 3H); ^{13}C{^{1}H} NMR (175 MHz, $CDCl_3$): δ 144.9, 133.0, 131.8, 129.8, 128.8, 128.3, 127.8, 121.9, 86.0, 84.2, 79.8, 67.2, 52.8, 38.8, 21.6; HRMS (ESI-TOF) m/z: $[M+Na]^+$ calcd. $C_{19}H_{17}NO_3SNa$: 362.0827, found: 362.0827.

Spectral data *N*-butyl-*N*-(3-hydroxy-5-phenylpenta-1,4-diyn-1-yl)-4-methylbenzenesulfonamide (1b):

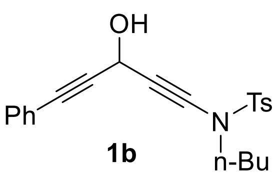

1b

Compound **1b** was purified on silica gel column using ethyl acetate/hexane: (30: 70) as the eluent; Yellow oil (510 mg, 1.34 mmol, 67%); ^{1}H NMR (700 MHz, $CDCl_3$): δ 7.80 (d, *J* = 8.4 Hz, 2H), 7.45 ~ 7.44 (m, 2H), 7.34 ~ 7.30 (m, 3H), 7.26 (d, *J* = 8.2 Hz, 2H), 5.43 (d, *J* = 7.3 Hz, 1H), 3.37 ~ 3.34 (m, 1H), 3.30 ~ 3.26 (m, 1H), 2.48 (d, *J* = 7.5 Hz, 1H), 2.38 (s, 3H), 1.63 ~ 1.60 (m, 2H), 1.33 (q, *J* = 7.2 Hz, 2H), 0.89 (t, *J* = 7.4 Hz, 3H); ^{13}C{^{1}H} NMR (175 MHz, $CDCl_3$): δ 144.7, 134.4, 131.8, 129.8, 128.8, 128.3, 127.7, 122.0, 86.1, 84.2, 78.5, 68.9, 52.9, 51.0, 29.9, 21.6, 19.4, 13.5; HRMS (ESI-TOF) m/z: $[M+Na]^+$ calcd. $C_{22}H_{23}NO_3SNa$: 404.1296, found: 404.1296.

Spectral data of *N*-(3-hydroxy-5-phenylpenta-1,4-diyn-1-yl)-*N*-isopropyl-4-methylbenzenesulfonamide (1c):

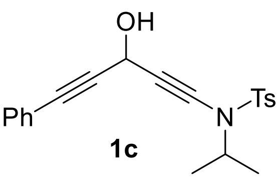

1c

Compound **1c** was purified on silica gel column using ethyl acetate/hexane: (30: 70) as the eluent; Yellow oil (540 mg, 1.47 mmol, 70%); ^{1}H NMR (700 MHz, $CDCl_3$): δ 7.80 (d, *J* = 7.0 Hz, 2H), 7.44 (d, *J* = 6.3 Hz, 2H), 7.36 ~ 7.31 (m, 3H), 7.255 ~ 7.24 (m, 2H), 5.46 (d, *J* = 7.0 Hz, 1H), 4.17 ~ 4.14 (m, 1H), 2.49 (d, *J* = 7.0 Hz, 1H), 2.38 (s, 3H), 1.14 (d, *J* = 6.3 Hz, 3H), 1.09 (d, *J* = 5.6 Hz, 3H); ^{13}C{^{1}H} NMR (175 MHz, $CDCl_3$): δ 144.6, 135.7, 131.8, 129.8, 128.8, 128.3, 127.5, 122.1, 86.3, 84.1, 75.7, 71.2, 53.0, 52.6, 21.6, 21.0, 20.6; HRMS (ESI-TOF) m/z: $[M+Na]^+$ calcd. $C_{21}H_{21}NO_3SNa$: 390.1140, found: 390.1144.

Spectral data of *N*-cyclopropyl-*N*-(3-hydroxy-5-phenylpenta-1,4-diyn-1-yl)-4-methylbenzenesulfonamide (1d):

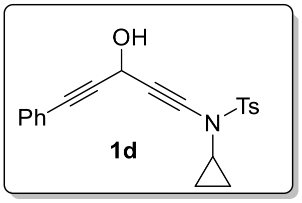

Compound **1d** was purified on silica gel column using ethyl acetate/hexane: (30: 70) as the eluent; Yellow oil (520 mg, 1.42 mmol, 67%); ^{1}H NMR (700 MHz, $CDCl_3$): δ 7.83 (d, *J* = 8.2 Hz, 2H), 7.45 ~ 7.44 (m, 2H), 7.33 ~ 7.31 (m, 3H), 7.28 (d, *J* = 8.2 Hz, 2H), 5.42 (d, *J* = 7.5 Hz, 1H), 2.76 ~ 2.74 (m, 1H), 2.46 (d, *J* = 7.6 Hz, 1H), 2.39 (s, 3H), 0.89 ~ 0.86 (m, 1H), 0.83 ~ 0.73 (m, 3H); ^{13}C{^{1}H} NMR (175 MHz, $CDCl_3$): δ 144.9, 133.7, 131.8, 129.7, 128.8, 128.3, 128.0, 122.0, 86.1, 84.2, 78.0, 68.8, 52.9, 32.6, 21.6, 6.8, 6.4; HRMS (ESI-TOF) m/z: $[M+Na]^+$ calcd. $C_{21}H_{19}NO_3SNa$: 388.0983, found: 388.0985.

Spectral data of *N*-cyclohexyl-*N*-(3-hydroxy-5-phenylpenta-1,4-diyn-1-yl)-4-methylbenzenesulfonamide (1e):

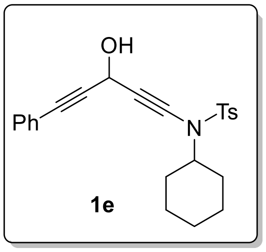

Compound **1e** was purified on silica gel column using ethyl acetate/hexane: (30: 70) as the eluent; Yellow oil (550 mg, 1.35 mmol, 75%); ^{1}H NMR (700 MHz, $CDCl_3$): δ 7.80 (d, *J* = 7.7 Hz, 2H), 7.44 (d, *J* = 6.3 Hz, 2H), 7.32 ~ 7.31 (m, 3H), 7.23 (d, *J* = 5.6 Hz, 2H), 5.45 (s, 1H), 3.77 ~ 3.74 (m, 1H), 2.52 (bs, 1H), 2.37 (s, 3H), 1.73 ~ 1.66 (m, 4H), 1.60 ~ 1.42 (m, 3H), 1.28 ~ 1.24 (m, 2H), 1.02 (q, *J* = 12.8 Hz, 1H); ^{13}C{^{1}H} NMR (175 MHz, $CDCl_3$): δ 144.5, 135.9, 131.8, 129.7, 128.8, 128.3, 127.5, 122.1, 86.3, 84.1, 76.5, 70.8, 59.5, 53.0, 31.3, 30.9, 25.4, 25.3, 24.8, 21.6; HRMS (ESI-TOF) m/z: $[M+Na]^+$ calcd. $C_{24}H_{25}NO_3SNa$: 430.1453, found: 430.1453.

Spectral data of *N*-(3-hydroxy-5-phenylpenta-1,4-diyn-1-yl)-4-methyl-*N*-phenylbenzenesulfonamide (1f):

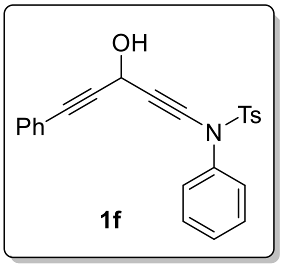

Compound **1f** was purified on silica gel column using ethyl acetate/hexane: (30: 70) as the eluent; Yellow oil (660 mg, 1.64 mmol, 89%); ^{1}H NMR (700 MHz, $CDCl_3$): δ 7.59 (d, *J* = 8.4 Hz, 2H), 7.45 (dd, *J* = 7.7, 1.4 Hz, 2H), 7.34 ~ 7.31 (m, 6H), 7.24 ~ 7.22 (m, 2H), 7.20 (d, *J* = 7.7 Hz, 2H), 5.46 (d, *J* = 6.3 Hz, 1H), 2.43 (d, *J* = 5.6 Hz, 1H), 2.38 (s, 3H); ^{13}C{^{1}H} NMR (175 MHz, $CDCl_3$): δ 145.1, 138.3, 132.8, 131.8, 129.6, 129.1, 128.9, 128.5, 128.35, 128.32, 126.3, 121.9, 85.9, 84.4, 78.9, 68.5, 53.0, 21.7; HRMS (ESI-TOF) m/z: $[M+Na]^+$ calcd. $C_{24}H_{19}NO_3SNa$: 424.0983, found: 424.0987.

Spectral data of *N*-(3-hydroxy-5-phenylpenta-1,4-diyn-1-yl)-4-methyl-*N*-(p-tolyl)benzenesulfonamide (1g):

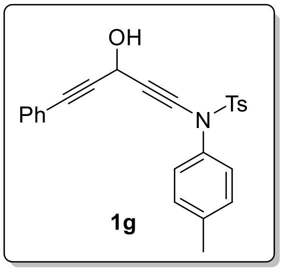

Compound **1g** was purified on silica gel column using ethyl acetate/hexane: (30: 70) as the eluent; Yellow oil (620 mg, 1.49 mmol, 85%); ^{1}H NMR (700 MHz, $CDCl_3$): δ 7.60 (d, *J* = 7.0 Hz, 2H), 7.44 (d, *J* = 7.0 Hz, 2H), 7.32 ~ 7.31 (m, 3H), 7.20 (d, *J* = 7.0 Hz, 2H), 7.11 ~ 7.08 (m, 4H), 5.44 (s, 1H), 2.47 (s, 1H), 2.37 (s, 3H), 2.32 (s, 3H); ^{13}C{^{1}H} NMR (175 MHz, $CDCl_3$): δ 145.0, 138.7, 135.6, 132.8, 131.8, 129.8, 129.5, 128.8, 128.33, 128.32, 126.3, 121.9, 86.0, 84.3, 79.1, 68.2, 52.9, 21.6, 21.1; HRMS (ESI-TOF) m/z: $[M+Na]^+$ calcd. $C_{25}H_{21}NO_3SNa$: 438.1140, found: 438.1148.

Spectral data of *N*-(4-bromophenyl)-*N*-(3-hydroxy-5-phenylpenta-1,4-diyn-1-yl)-4-methylbenzenesulfonamide (1h):

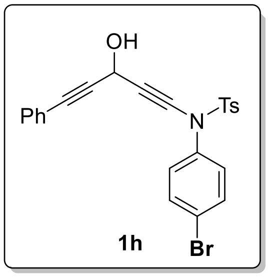

Compound **1h** was purified on silica gel column using ethyl acetate/hexane: (30: 70) as the eluent; Yellow oil (420 mg, 0.87 mmol, 61%); ^{1}H NMR (700 MHz, $CDCl_3$): δ 7.59 (d, *J* = 7.7 Hz, 2H), 7.45 ~ 7.42 (m, 4H), 7.34 ~ 7.30 (m, 3H), 7.21 (d, *J* = 7.7 Hz, 2H), 7.11 (d, *J* = 7.7 Hz, 2H), 5.45 (d, *J* = 7.0 Hz, 1H), 2.63 (d, *J* = 6.3 Hz, 1H), 2.37 (s, 3H); $^{13}C\{^{1}H\}$ NMR (175 MHz, $CDCl_3$): δ 145.4, 137.3, 132.4, 132.3, 131.8, 129.7, 128.9, 128.4, 128.3, 127.7, 122.3, 121.9, 85.8, 84.5, 78.2, 69.0, 52.9, 21.7; HRMS (ESI-TOF) m/z: $[M+Na]^+$ calcd. $C_{24}H_{18}BrNO_3SNa$: 502.0088, found: 502.0089.

Spectral data of *N*-(3-hydroxy-5-phenylpenta-1,4-diyn-1-yl)-*N*-methylmethanesulfonamide (1i):

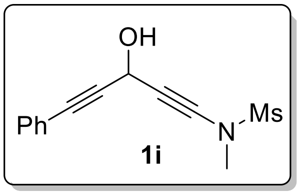

Compound **1i** was purified on silica gel column using ethyl acetate/hexane: (30: 70) as the eluent; Yellow oil (470 mg, 1.78 mmol, 48%); ^{1}H NMR (700 MHz, $CDCl_3$): δ 7.43 (dd, *J* = 7.7, 0.7 Hz, 2H), 7.32 ~ 7.28 (m, 3H), 5.48 (d, *J* = 6.3 Hz, 1H), 3.22 (s, 3H), 3.08 (s, 3H); 2.58 (d, *J* = 6.3 Hz, 1H); $^{13}C\{^{1}H\}$ NMR (175 MHz, $CDCl_3$): δ 131.8, 128.9, 128.3, 121.8, 85.7, 84.4, 78.9, 67.7, 52.8, 38.8, 37.2; HRMS (ESI-TOF) m/z: $[M+Na]^+$ calcd. $C_{13}H_{13}NO_3SNa$: 286.0514, found: 286.0513.

Spectral data of 3-(3-hydroxy-5-phenylpenta-1,4-diyn-1-yl)oxazolidin-2-one (1j):

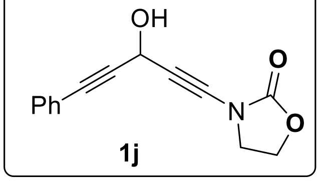

Compound **1j** was purified on silica gel column using ethyl acetate/hexane: (30: 70) as the eluent; Yellow oil (730 mg, 3.03 mmol, 67%); ^{1}H NMR (700 MHz, $CDCl_3$): δ 7.43 (dd, *J* = 7.0 Hz, 2H), 7.31 ~ 7.27 (m, 3H), 5.51 (s, 1H), 4.41 (t, *J* = 8.4 Hz, 2H), 3.91 (t, *J* = 8.4 Hz, 2H), 3.27 (bs, 1H); ^{13}C{^{1}H} NMR (175 MHz, $CDCl_3$): δ 156.1, 131.8, 128.8, 128.3, 121.9, 85.8, 84.3, 74.6, 69.4, 63.3, 52.6, 46.5; HRMS (ESI-TOF) m/z: $[M+Na]^+$ calcd. $C_{14}H_{11}NO_3Na$: 264.0637, found: 264.0638.

Spectral data of *N*-(3-hydroxy-5-(*p*-tolyl)penta-1,4-diyn-1-yl)-*N*,4-dimethylbenzenesulfonamide (1k):

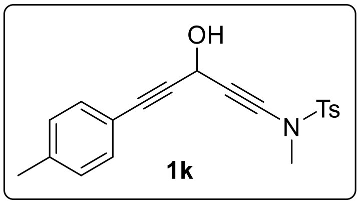

Compound **1k** was purified on silica gel column using ethyl acetate/hexane: (30: 70) as the eluent; Yellow oil (720 mg, 2.04 mmol, 85%); ^{1}H NMR (700 MHz, $CDCl_3$): δ 7.78 (d, *J* = 7.0 Hz, 2H), 7.32 (d, *J* = 7.7 Hz, 2H), 7.27 ~ 7.26 (m, 2H), 7.09 (d, *J* = 7.7 Hz, 2H), 5.41 (d, *J* = 5.6 Hz, 1H), 3.04 (s, 3H), 2.79 (bs, 1H), 2.37 (s, 3H), 2.32 (s, 3H); ^{13}C{^{1}H} NMR (175 MHz, $CDCl_3$): δ 144.9, 138.9, 132.9, 131.6, 129.8, 129.0, 127.8, 118.8, 85.4, 84.3, 79.6, 67.3, 52.7, 38.8, 21.5, 21.4; HRMS (ESI-TOF) m/z: $[M+Na]^+$ calcd. $C_{20}H_{19}NO_3SNa$: 376.0983, found: 376.0981.

Spectral data of *N*-(5-(4-chlorophenyl)-3-hydroxypenta-1,4-diyn-1-yl)-*N*,4-dimethylbenzenesulfonamide (1l):

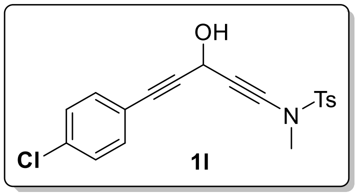

Compound **1l** was purified on silica gel column using ethyl acetate/hexane: (30: 70) as the eluent; Yellow oil (650 mg, 1.74 mmol, 73%); ^{1}H NMR (700 MHz, $CDCl_3$): δ 7.79 (d, *J* = 8.4 Hz, 2H), 7.37 (d, *J* = 8.4 Hz, 2H), 7.30 ~ 7.28 (m, 4H), 5.41 (d, *J* = 6.3 Hz, 1H), 3.07 (s, 3H), 2.45 (d, *J* = 7.0 Hz, 1H), 2.41 (s, 3H); ^{13}C{^{1}H} NMR (175 MHz, $CDCl_3$): δ 145.0, 135.0, 133.1, 133.0, 129.9,

128.7, 127.8, 120.4, 86.9, 83.1, 80.0, 67.0, 52.8, 38.8, 21.6; HRMS (ESI-TOF) m/z: $[M+Na]^+$ calcd. $C_{19}H_{16}ClNO_3SNa$: 396.0437, found: 396.0434.

Spectral data of N-(3-hydroxy-5-(4-methoxyphenyl)penta-1,4-diyn-1-yl)-N,4-dimethylbenzenesulfonamide (1m):

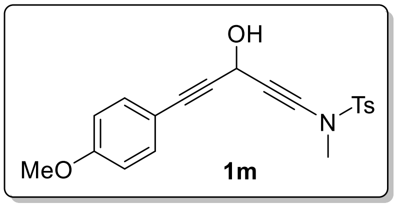

Compound **1m** was purified on silica gel column using ethyl acetate/hexane: (40: 60) as the eluent; Yellow oil (620 mg, 1.67 mmol, 70%); ^{1}H NMR (400 MHz, $CDCl_3$): δ 7.80 (d, J = 8.4 Hz, 2H), 7.38 ~ 7.35 (m, 2H), 7.28 (d, J = 8.4 Hz, 2H), 6.83 ~ 6.80 (m, 2H), 5.41 (d, J = 6.0 Hz, 1H), 3.78 (s, 3H), 3.04 (s, 3H), 2.75 (s, 1H), 2.38 (s, 3H); ^{13}C{^{1}H} NMR (100 MHz, $CDCl_3$): δ 159.4, 144.9, 133.2, 132.9, 129.8, 127.8, 113.9, 84.7, 84.2, 79.5, 67.4, 55.2, 52.8, 38.8, 21.5; HRMS-ESI$^+$ m/z: $[M+Na]^+$ calcd. $C_{20}H_{19}NO_4SNa$: 392.0932, found: 392.0931.

Spectral data of N-(3-hydroxy-5-(o-tolyl)penta-1,4-diyn-1-yl)-N,4-dimethylbenzenesulfonamide (1n):

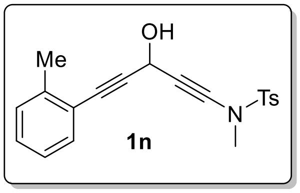

Compound **1n** was purified on silica gel column using ethyl acetate/hexane: (30:70) as the eluent; Yellow oil (450 mg, 1.27 mmol, 53%); ^{1}H NMR (400 MHz, $CDCl_3$): δ 7.80 (d, J = 8.4 Hz, 2H), 7.41 (d, J = 7.6 Hz, 1H), 7.28 (d, J = 8.0 Hz, 2H), 7.22 ~ 7.17 (m, 2H), 7.12 (m, 1H) 5.46 (d, J = 6.8 Hz, 1H), 3.06 (s, 3H), 2.61 (d, J = 7.2 Hz, 1H), 2.41 (s, 3H), 2.38 (s, 3H); ^{13}C{^{1}H} NMR (100 MHz, $CDCl_3$): δ 144.9, 140.5, 133.1, 132.1, 129.8, 129.4, 128.8, 127.8, 125.5, 121.7, 89.9, 83.2, 79.7, 67.4, 52.9, 38.8, 21.5, 20.5; HRMS-ESI$^+$ m/z: $[M+Na]^+$ calcd. $C_{20}H_{19}NO_3SNa$: 376.0983, found: 376.0983.

Spectral data of *N*-(3-hydroxynona-1,4-diyn-1-yl)-*N*,4-dimethylbenzenesulfonamide (1o):

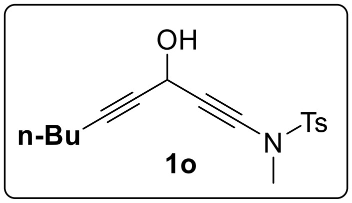

Compound **1o** was purified on silica gel column using ethyl acetate/hexane: (30: 70) as the eluent; Yellow oil (590 mg, 1.85 mmol, 77%); ^{1}H NMR (700 MHz, $CDCl_3$): δ 7.77 (d, *J* = 7.7 Hz, 2H), 7.33 (d, *J* = 7.7 Hz, 2H), 5.18 (d, *J* = 5.6 Hz, 1H), 3.04 (s, 3H), 2.43 (s, 3H), 2.28 (d, *J* = 7.0 Hz, 1H), 2.22 (t, *J* = 6.3 Hz, 2H), 1.50 ~ 1.47 (m, 2H), 1.40 (q, *J* = 7.0 Hz, 2H), 0.89 (t, *J* = 7.0 Hz, 3H); ^{13}C{^{1}H} NMR (175 MHz, $CDCl_3$): δ 144.9, 133.1, 129.8, 127.8, 85.5, 79.1, 76.8, 67.7, 52.5, 38.8, 30.4, 21.9, 21.6, 18.4, 13.6; HRMS (ESI-TOF) m/z: $[M+Na]^+$ calcd. $C_{17}H_{21}NO_3SNa$: 342.1140, found: 342.1135.

Spectral data of *N*-(5-cyclopropyl-3-hydroxypenta-1,4-diyn-1-yl)-*N*,4-dimethylbenzenesulfonamide (1p):

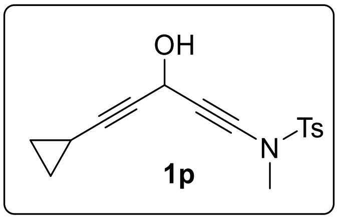

Compound **1p** was purified on silica gel column using ethyl acetate/hexane: (30: 70) as the eluent; Yellow oil (530 mg, 1.75 mmol, 73%); ^{1}H NMR (400 MHz, $CDCl_3$): δ 7.77 (d, *J* = 8.4 Hz, 2H), 7.34 (d, *J* = 8.4 Hz, 2H), 5.14 (d, *J* = 6.8 Hz, 1H), 3.03 (s, 3H), 2.44 (s, 3H), 2.28 (d, *J* = 6.8 Hz, 1H), 1.29 ~ 1.24 (m, 1H), 0.81 ~ 0.69 (m, 4H); ^{13}C{^{1}H} NMR (175 MHz, $CDCl_3$): δ 144.9, 133.1, 129.8, 129.7, 127.9, 127.8, 88.5, 79.2, 72.5, 67.6, 52.5, 38.8, 27.3, 21.7; HRMS (ESI-TOF) m/z: $[M+Na]^+$ calcd. $C_{16}H_{17}NO_3SNa$: 326.0827, found: 326.0824.

Spectral data of *N*-(3-hydroxy-5-(thiophen-2-yl)penta-1,4-diyn-1-yl)-*N*,4-dimethylbenzenesulfonamide (1q):

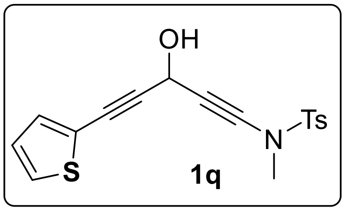

Compound **1q** was purified on silica gel column using ethyl acetate/hexane: (30: 70) as the eluent; Yellow oil (610 mg, 1.77 mmol, 74%); ^{1}H NMR (700 MHz, $CDCl_3$): δ 7.79 (d, *J* = 7.7 Hz, 2H), 7.30 (d, *J* = 7.7 Hz, 2H), 7.27 (d, *J* = 4.2 Hz, 1H), 7.24 (s, 1H), 6.97 (s, 1H), 5.42 (d, *J* = 6.3 Hz, 1H), 3.06 (s, 3H), 2.55 (d, *J* = 6.3 Hz, 1H), 2.40 (s, 3H); ^{13}C{^{1}H} NMR (175 MHz, $CDCl_3$): δ 145.0, 133.0, 132.9, 129.9, 127.8, 127.0, 121.8, 89.7, 80.0, 77.7, 66.9, 52.9, 38.8, 21.6; one carbon merge with other peak; HRMS (ESI-TOF) m/z: $[M+Na]^+$ calcd. $C_{17}H_{15}NO_3S_2Na$: 368.0391, found: 368.0392.

Spectral data of *N*-(3-hydroxy-6-methylhepta-6-en-1,4-diyn-1-yl)-*N*,4-dimethylbenzenesulfonamide (1r):

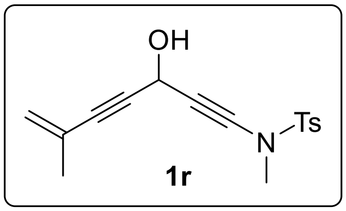

Compound **1r** was purified on silica gel column using ethyl acetate/hexane: (30: 70) as the eluent; Yellow oil (420 mg, 1.38 mmol, 58%); ^{1}H NMR (700 MHz, $CDCl_3$): δ 7.78 (d, *J* = 7.0 Hz, 2H), 7.33 (d, *J* = 7.7 Hz, 2H), 5.34 (s, 1H), 5.31 (d, *J* = 6.3 Hz, 1H), 5.28 (d, *J* = 1.4 Hz, 1H), 3.05 (s, 3H), 2.43 (s, 3H), 2.36 (d, *J* = 6.3 Hz, 1H), 1.89 (s, 3H) ; ^{13}C{^{1}H} NMR (175 MHz, $CDCl_3$): δ 144.9, 133.1, 129.8, 127.9, 125.8, 123.2, 85.4, 84.9, 79.7, 67.2, 52.7, 38.8, 23.1, 21.6; HRMS (ESI-TOF) m/z: $[M+Na]^+$ calcd. $C_{16}H_{17}NO_3SNa$: 326.0827, found: 326.0828.

5. Computational Details:

Geometries were optimized using the B3LYP-D3 functional combined with the def2-SVP basis. Hessians at these geometries provided the zero-point energy and vibrational enthalpy and entropy. Solvation free energies (G_{solv}) were computed using the CPCM continuum model. The electronic

energy E_{elec} was obtained with the same functional and the def2-TZVP basis. The Gibbs free energy of each species was computed according to the following equation:

$$G = E_{elec} + G_{solv} + ZPE + H_{vib} + 4kT - TS_{vib}$$

Table S1. The energy components to form the Gibbs free energy for each stationary point along the reaction pathway.

Species	E_{elec}	G_{solv}	$ZPE + H_{vib} + 4kT - TS_{vib}$	G_{total}(hartree)
LAu	-1410.136904	-0.0606892	0.44085	-1409.756743
Ag^{+}	-146.7374663	-0.141548272	-0.016605	-146.8956195
8-methylquinoline N-oxide	-516.6425993	0.011019024	0.13396	-516.4976203
8-methylquinoline	-441.4482327	0.007996996	0.130327	-441.3099087
1a	-1413.221566	-0.01027368	0.259568	-1412.972272
A	-1560.073571	-0.07123766	0.254607	-1559.890202
B	-2076.751877	-0.07129073	0.415851	-2076.407317
C	-1635.344037	-0.07423643	0.260423	-1635.157851
D	-1635.392006	-0.08226038	0.263865	-1635.210401
E	-1635.010507	-0.03170008	0.252182	-1634.790025
F	-1488.549772	-0.01415995	0.26316	-1488.300772
G	-1635.39481	-0.08120848	0.257674	-1635.218344
H	-1635.018977	-0.02402478	0.252687	-1634.790315
I	-1635.042561	-0.01452279	0.256133	-1634.800951
J (3a)	-1488.613379	-0.01173219	0.269323	-1488.355788
g	-1635.4043	-0.07001089	0.259504	-1635.214806
h	-1635.403844	-0.07183088	0.265123	-1635.210552
i	-1635.022762	-0.02771809	0.254789	-1634.795691
j (3a')	-1488.59939	-0.011960	0.269341	-1488.3420
ts-AB	-2076.720707	-0.06924247	0.411566	-2076.378384
ts-BC	-2076.727492	-0.06840272	0.411131	-2076.384764
ts-CD	-1635.311281	-0.07686878	0.261891	-1635.126259
ts-HI	-1634.999766	-0.02400829	0.252619	-1634.771155
ts-hi	-1634.99788	-0.025608	0.253798	-1634.7697

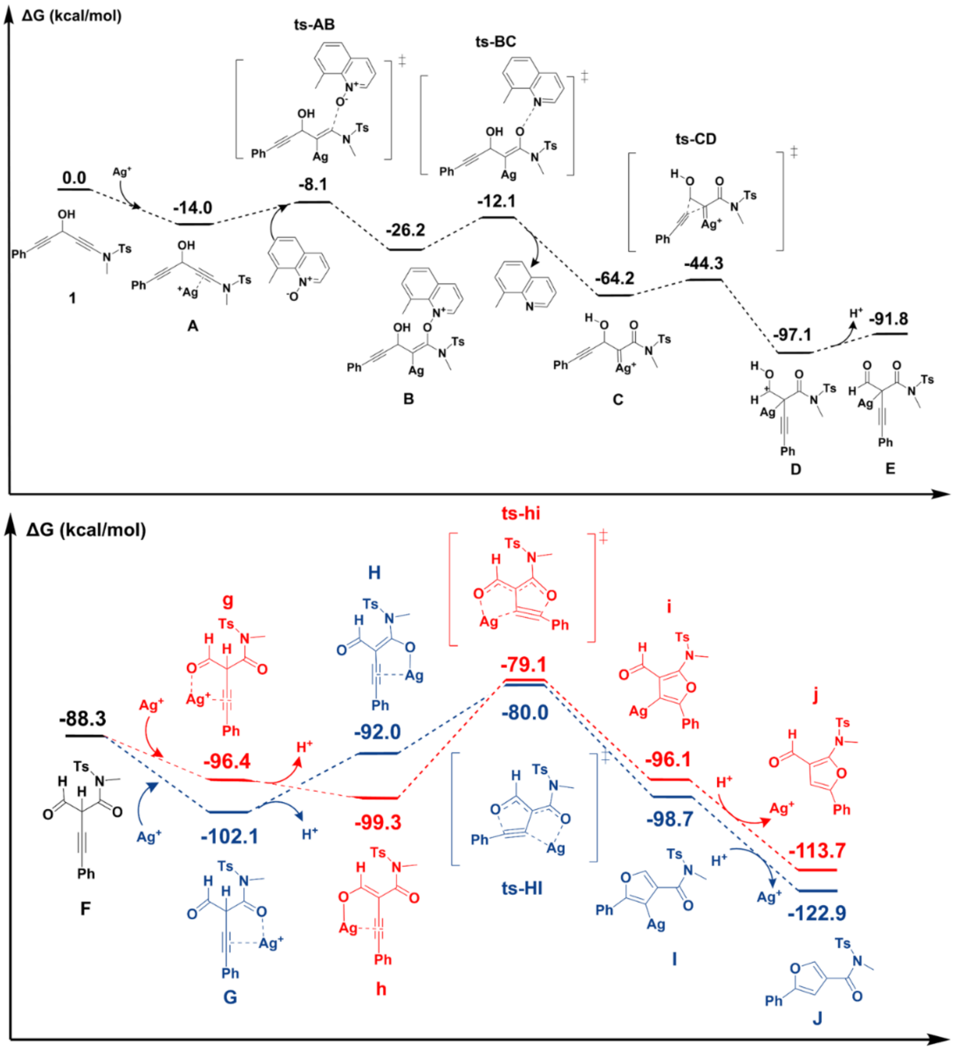

6. Figure s1: Gibbs Free Energy Profiles for two independent routes

7. X-ray crystallographic structure and data for compound 3s:

X-ray crystallographic data of compound (3a).

Ellipsoid contour % probability level = 50%

Experimental: The sample was dissolved in appropriate amount of Ethyl Acetate followed by the addition of pentane to furnish a saturated solution. Afterwards, the mixture was allowed to stand at room temperature to form the crystals.

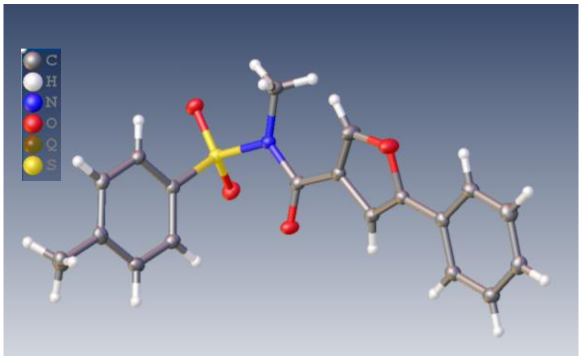

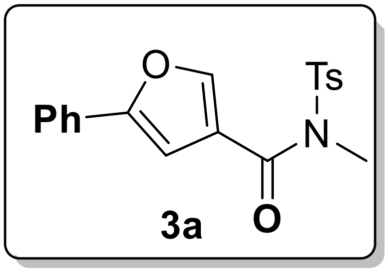

CCDC-2357513

ORTEP diagram of compounds **3a**

231232LT_auto

Table s2 Crystal data and structure refinement for 231232LT_auto.

Identification code	231232LT_auto
Empirical formula	$C_{19}H_{17}NO_4S$
Formula weight	355.39
Temperature/K	100.00(10)
Crystal system	monoclinic
Space group	$P2_1/c$
a/Å	19.0633(2)
b/Å	5.70844(5)
c/Å	16.03289(17)
α/°	90
β/°	108.5380(12)
γ/°	90
Volume/Å^3	1654.20(3)
Z	4
ρ_{calc}g/cm^3	1.427
μ/mm^{-1}	1.953
F(000)	744.0
Crystal size/mm^3	0.17 × 0.16 × 0.12
Radiation	Cu Kα (λ = 1.54184)
2Θ range for data collection/°	4.89 to 149.148
Index ranges	$-23 \leq h \leq 21, -7 \leq k \leq 6, -14 \leq l \leq 20$
Reflections collected	13685
Independent reflections	3172 [R_{int} = 0.0180, R_{sigma} = 0.0148]
Data/restraints/parameters	3172/0/228
Goodness-of-fit on F^2	1.028

Final R indexes [I>=2σ (I)]	R_1 = 0.0323, wR_2 = 0.0854
Final R indexes [all data]	R_1 = 0.0334, wR_2 = 0.0864
Largest diff. peak/hole / e $Å^{-3}$	0.29/-0.45

Table s3 Fractional Atomic Coordinates (×10^4) and Equivalent Isotropic Displacement Parameters ($Å^2$×10^3) for 231232LT_auto. U_{eq} is defined as 1/3 of the trace of the orthogonalised U_{IJ} tensor.

Atom	*x*	*y*	*z*	U(eq)
S7	1707.2(2)	3276.7(6)	1305.1(2)	15.53(11)
O10	2955.9(5)	1485.8(17)	2670.5(6)	21.1(2)
O12	1317.7(5)	4687.2(18)	563.5(6)	20.5(2)
O13	1721.7(5)	791.5(18)	1202.1(6)	20.3(2)
O22	4828.7(6)	6691.4(19)	3260.4(7)	25.9(3)
N8	2585.1(6)	4334(2)	1627.7(7)	16.3(2)
C1	1370.7(7)	3947(2)	2179.0(9)	15.4(3)
C2	1426.5(7)	2309(2)	2841.2(9)	17.4(3)
C3	1132.1(7)	2857(3)	3505.4(9)	19.0(3)
C4	783.1(7)	5003(3)	3514.3(9)	18.5(3)
C5	741.4(7)	6615(2)	2846.0(9)	18.6(3)
C6	1033.3(7)	6110(2)	2176.0(9)	17.7(3)
C9	3103.1(7)	3285(2)	2348.2(9)	16.3(3)
C11	2728.0(8)	6523(3)	1225.1(10)	22.4(3)
C14	447.4(8)	5563(3)	4223.8(9)	25.3(3)
C15	5706.8(7)	4421(2)	4393.4(9)	16.7(3)
C16	6243.5(8)	6163(3)	4495.4(9)	20.1(3)
C17	6925.9(8)	5957(3)	5148.5(10)	22.7(3)
C18	7079.4(8)	4034(3)	5704.9(9)	21.2(3)
C19	6550.4(8)	2279(3)	5605.9(9)	20.7(3)
C20	5869.8(8)	2476(3)	4954.0(9)	19.5(3)
C21	4984.7(7)	4605(2)	3715.7(9)	17.1(3)
C23	4137.4(8)	6502(3)	2676.7(10)	24.1(3)
C24	3842.2(7)	4361(2)	2741.0(9)	16.4(3)
C25	4399.4(8)	3152(2)	3421.6(9)	17.5(3)

Table s4 Anisotropic Displacement Parameters (Å^2×10^3) for 231232LT_auto. The Anisotropic displacement factor exponent takes the form: $-2\pi^2[h^2a^{*2}U_{11}+2hka^*b^*U_{12}+\ldots]$.

Atom	U_{11}	U_{22}	U_{33}	U_{23}	U_{13}	U_{12}
S7	16.09(17)	15.83(19)	13.28(18)	-0.38(12)	2.69(13)	1.17(11)
O10	20.5(5)	18.7(5)	20.7(5)	5.6(4)	1.8(4)	-2.3(4)
O12	20.8(5)	23.8(5)	14.4(5)	2.0(4)	1.9(4)	3.3(4)
O13	21.9(5)	16.5(5)	20.1(5)	-3.6(4)	3.5(4)	0.7(4)
O22	22.2(5)	23.2(6)	27.2(6)	8.2(4)	0.6(4)	-5.7(4)
N8	16.6(5)	16.1(6)	16.0(5)	2.7(4)	5.1(4)	0.6(4)
C1	14.0(6)	16.0(6)	14.8(6)	-1.2(5)	2.6(5)	-2.1(5)
C2	15.7(6)	16.2(7)	17.8(6)	0.6(5)	2.0(5)	-1.0(5)
C3	17.5(6)	21.9(7)	15.8(6)	2.7(5)	2.7(5)	-3.2(5)
C4	13.5(6)	23.8(7)	16.4(6)	-3.7(6)	2.1(5)	-3.9(5)
C5	15.6(6)	17.2(7)	21.2(7)	-2.9(5)	3.4(5)	-0.1(5)
C6	16.9(6)	16.1(7)	19.0(6)	1.5(5)	4.0(5)	-0.6(5)
C9	17.8(6)	15.6(7)	15.6(6)	-0.1(5)	5.7(5)	2.1(5)
C11	19.8(7)	21.1(7)	26.0(7)	9.6(6)	6.7(6)	1.8(6)
C14	21.3(7)	35.1(9)	20.4(7)	-2.8(6)	7.7(6)	-0.1(6)
C15	17.4(6)	18.6(7)	15.5(6)	-2.5(5)	7.0(5)	-0.1(5)
C16	22.3(7)	18.5(7)	20.1(7)	0.2(6)	7.7(6)	-2.0(6)
C17	20.9(7)	22.6(7)	23.9(7)	-4.7(6)	6.3(6)	-6.1(6)
C18	18.8(6)	25.4(8)	17.7(7)	-4.3(6)	3.2(5)	0.6(6)
C19	22.5(7)	22.0(7)	18.0(7)	1.3(6)	6.8(6)	1.1(6)
C20	19.4(6)	19.8(7)	20.6(7)	0.0(6)	8.2(6)	-2.4(5)
C21	19.0(6)	17.4(7)	16.6(6)	1.1(5)	8.0(5)	0.6(5)
C23	22.1(7)	22.8(8)	22.6(7)	6.7(6)	0.7(6)	-2.2(6)
C24	17.9(6)	17.5(7)	14.7(6)	0.8(5)	6.6(5)	1.7(5)
C25	18.3(6)	15.9(7)	18.0(7)	1.0(5)	5.6(5)	0.6(5)

Table s5 Bond Lengths for 231232LT_auto.

Atom	Atom	Length/Å	Atom	Atom	Length/Å
S7	O12	1.4325(10)	C4	C14	1.5063(19)
S7	O13	1.4295(10)	C5	C6	1.388(2)
S7	N8	1.6977(11)	C9	C24	1.4814(19)
S7	C1	1.7586(13)	C15	C16	1.3984(19)
O10	C9	1.2216(17)	C15	C20	1.400(2)
O22	C21	1.3790(17)	C15	C21	1.4612(18)
O22	C23	1.3564(18)	C16	C17	1.391(2)
N8	C9	1.3941(17)	C17	C18	1.386(2)
N8	C11	1.4707(18)	C18	C19	1.394(2)

Table s5 Bond Lengths for 231232LT_auto.

Atom	Atom	Length/Å	Atom	Atom	Length/Å
C1	C2	1.3931(19)	C19	C20	1.388(2)
C1	C6	1.3916(19)	C21	C25	1.3494(19)
C2	C3	1.388(2)	C23	C24	1.363(2)
C3	C4	1.396(2)	C24	C25	1.4346(18)
C4	C5	1.395(2)			

Table s6 Bond Angles for 231232LT_auto.

Atom	Atom	Atom	Angle/°	Atom	Atom	Atom	Angle/°
O12	S7	N8	104.69(6)	O10	C9	N8	120.95(12)
O12	S7	C1	108.35(6)	O10	C9	C24	118.96(12)
O13	S7	O12	119.07(6)	N8	C9	C24	120.09(12)
O13	S7	N8	109.56(6)	C16	C15	C20	118.94(13)
O13	S7	C1	109.47(6)	C16	C15	C21	121.14(13)
N8	S7	C1	104.71(6)	C20	C15	C21	119.91(12)
C23	O22	C21	107.17(11)	C17	C16	C15	120.15(13)
C9	N8	S7	118.00(9)	C18	C17	C16	120.44(13)
C9	N8	C11	123.20(11)	C17	C18	C19	119.92(13)
C11	N8	S7	118.07(9)	C20	C19	C18	119.79(14)
C2	C1	S7	120.16(11)	C19	C20	C15	120.75(13)
C6	C1	S7	118.40(10)	O22	C21	C15	116.69(12)
C6	C1	C2	121.42(13)	C25	C21	O22	109.31(12)
C3	C2	C1	118.81(13)	C25	C21	C15	133.99(13)
C2	C3	C4	121.04(13)	O22	C23	C24	110.67(12)
C3	C4	C14	120.80(13)	C23	C24	C9	134.38(13)
C5	C4	C3	118.81(13)	C23	C24	C25	105.37(12)
C5	C4	C14	120.38(13)	C25	C24	C9	119.87(12)
C6	C5	C4	121.20(13)	C21	C25	C24	107.47(12)
C5	C6	C1	118.70(13)				

Table s7 Torsion Angles for 231232LT_auto.

A	B	C	D	Angle/°	A	B	C	D	Angle/°
S7	N8	C9	O10	8.76(18)	C2	C3	C4	C14	178.41(12)
S7	N8	C9	C24	-170.59(10)	C3	C4	C5	C6	0.6(2)
S7	C1	C2	C3	-177.72(10)	C4	C5	C6	C1	0.1(2)
S7	C1	C6	C5	177.59(10)	C6	C1	C2	C3	0.5(2)

Table s7 Torsion Angles for 231232LT_auto.

A	B	C	D	Angle/°	A	B	C	D	Angle/°
O10	C9	C24	C23	-164.60(15)	C9	C24	C25	C21	-174.19(12)
O10	C9	C24	C25	7.1(2)	C11	N8	C9	O10	178.77(12)
O12	S7	N8	C9	-179.85(10)	C11	N8	C9	C24	-0.58(19)
O12	S7	N8	C11	9.62(12)	C14	C4	C5	C6	-178.58(12)
O12	S7	C1	C2	155.27(11)	C15	C16	C17	C18	-0.2(2)
O12	S7	C1	C6	-23.01(12)	C15	C21	C25	C24	179.30(14)
O13	S7	N8	C9	-51.09(11)	C16	C15	C20	C19	0.5(2)
O13	S7	N8	C11	138.39(10)	C16	C15	C21	O22	-7.72(19)
O13	S7	C1	C2	23.95(12)	C16	C15	C21	C25	173.37(15)
O13	S7	C1	C6	-154.33(10)	C16	C17	C18	C19	0.6(2)
O22	C21	C25	C24	0.33(16)	C17	C18	C19	C20	-0.5(2)
O22	C23	C24	C9	172.75(14)	C18	C19	C20	C15	-0.1(2)
O22	C23	C24	C25	0.17(17)	C20	C15	C16	C17	-0.3(2)
N8	S7	C1	C2	-93.43(11)	C20	C15	C21	O22	172.26(12)
N8	S7	C1	C6	88.29(11)	C20	C15	C21	C25	-6.6(2)
N8	C9	C24	C23	14.8(2)	C21	O22	C23	C24	0.02(17)
N8	C9	C24	C25	-173.50(12)	C21	C15	C16	C17	179.65(13)
C1	S7	N8	C9	66.23(11)	C21	C15	C20	C19	-179.50(12)
C1	S7	N8	C11	-104.30(11)	C23	O22	C21	C15	-179.39(12)
C1	C2	C3	C4	0.2(2)	C23	O22	C21	C25	-0.22(16)
C2	C1	C6	C5	-0.7(2)	C23	C24	C25	C21	-0.31(16)
C2	C3	C4	C5	-0.7(2)					

Table s8 Hydrogen Atom Coordinates (Å×10[4]) and Isotropic Displacement Parameters (Å[2]×10[3]) for 231232LT_auto.

Atom	*x*	*y*	*z*	U(eq)
H2	1661.8	843.69	2838.26	21
H3	1168.73	1754.67	3961.16	23
H5	508.95	8085.84	2849.69	22
H6	1003.24	7219.28	1724.16	21
H11A	2326.77	6802.48	674.52	34
H11B	3198.38	6393.89	1102.37	34
H11C	2754.03	7829.8	1630.09	34

Table s8 Hydrogen Atom Coordinates (Å×10^4) and Isotropic Displacement Parameters (Å^2×10^3) for 231232LT_auto.

**Atom**	***x***	***y***	***z***	**U(eq)**
H14A	797.08	6496.96	4684.03	38
H14B	337.31	4102.72	4478.79	38
H14C	-10.75	6453.1	3970.32	38
H16	6142	7489.25	4117.97	24
H17	7288.95	7143.44	5213.4	27
H18	7544.53	3910.9	6153.19	25
H19	6655.39	953.45	5983.5	25
H20	5510.48	1277.39	4887.4	23
H23	3892.63	7697.95	2277.98	29
H25	4362.91	1610.96	3627.6	21

8. ^{1}H and ^{13}C spectra

^{1}H NMR ($CDCl_3$, 400 MHz)

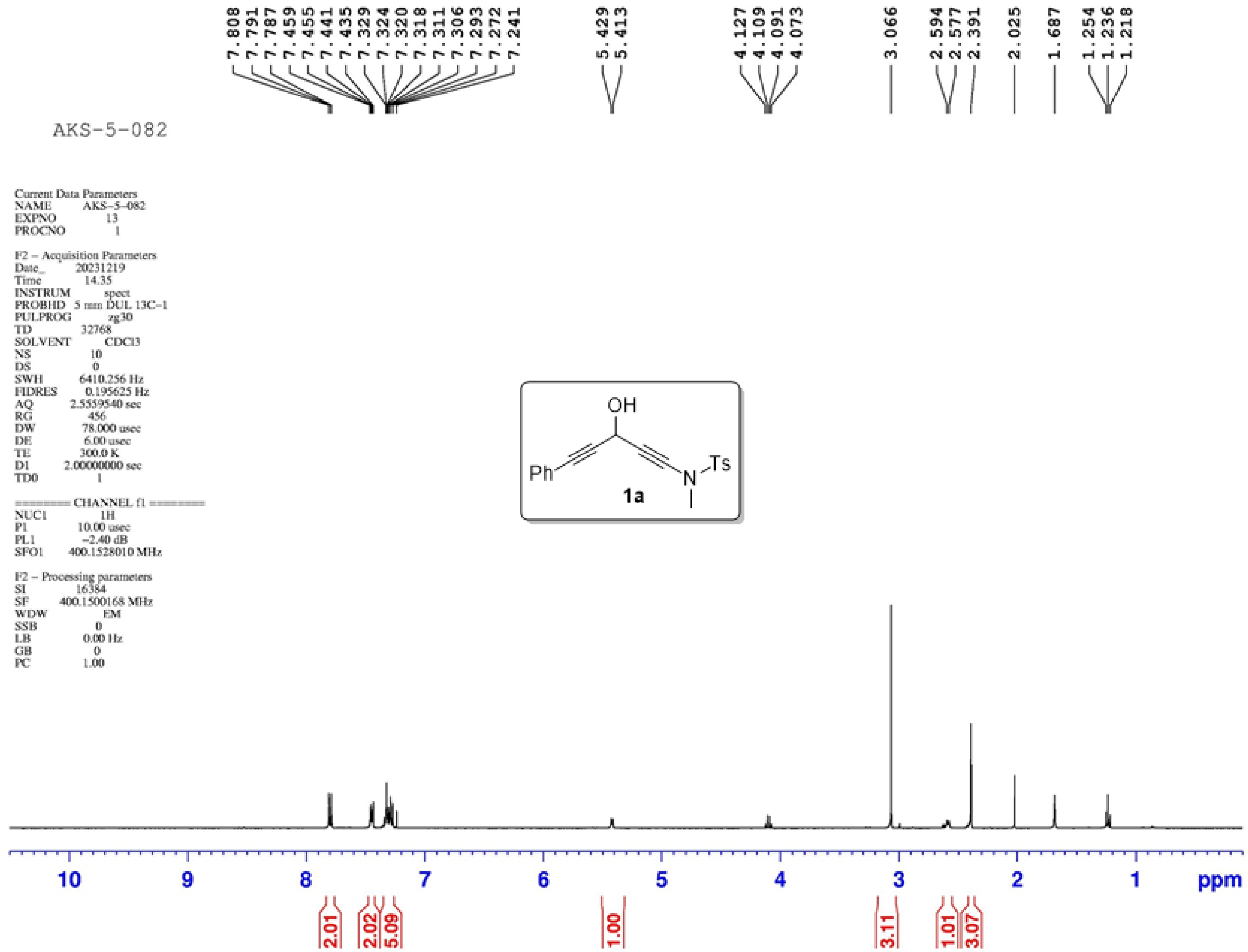

$^{13}C\{1H\}$ and DEPT NMR ($CDCl_3$, 100 MHz)

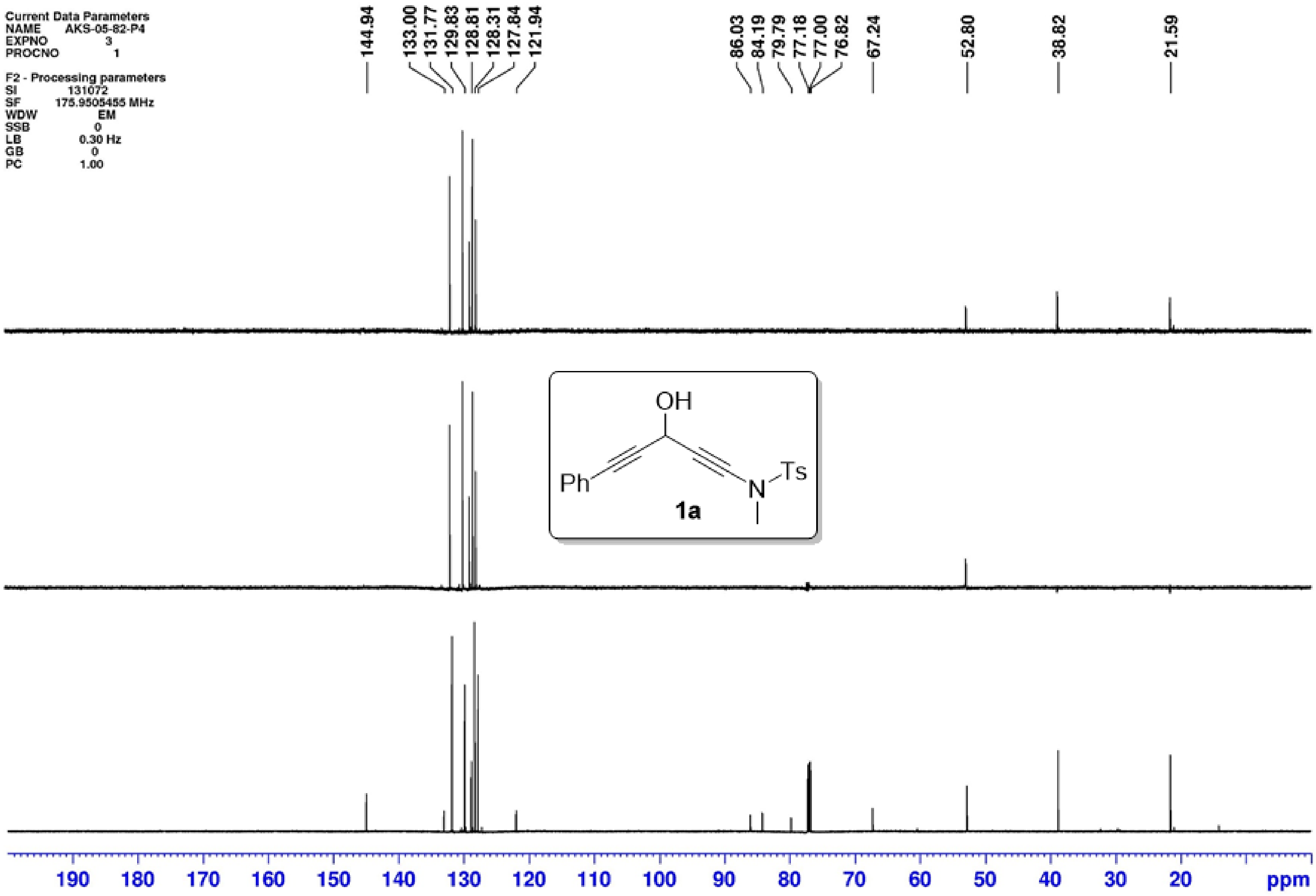

^{1}H NMR ($CDCl_3$, 700 MHz)

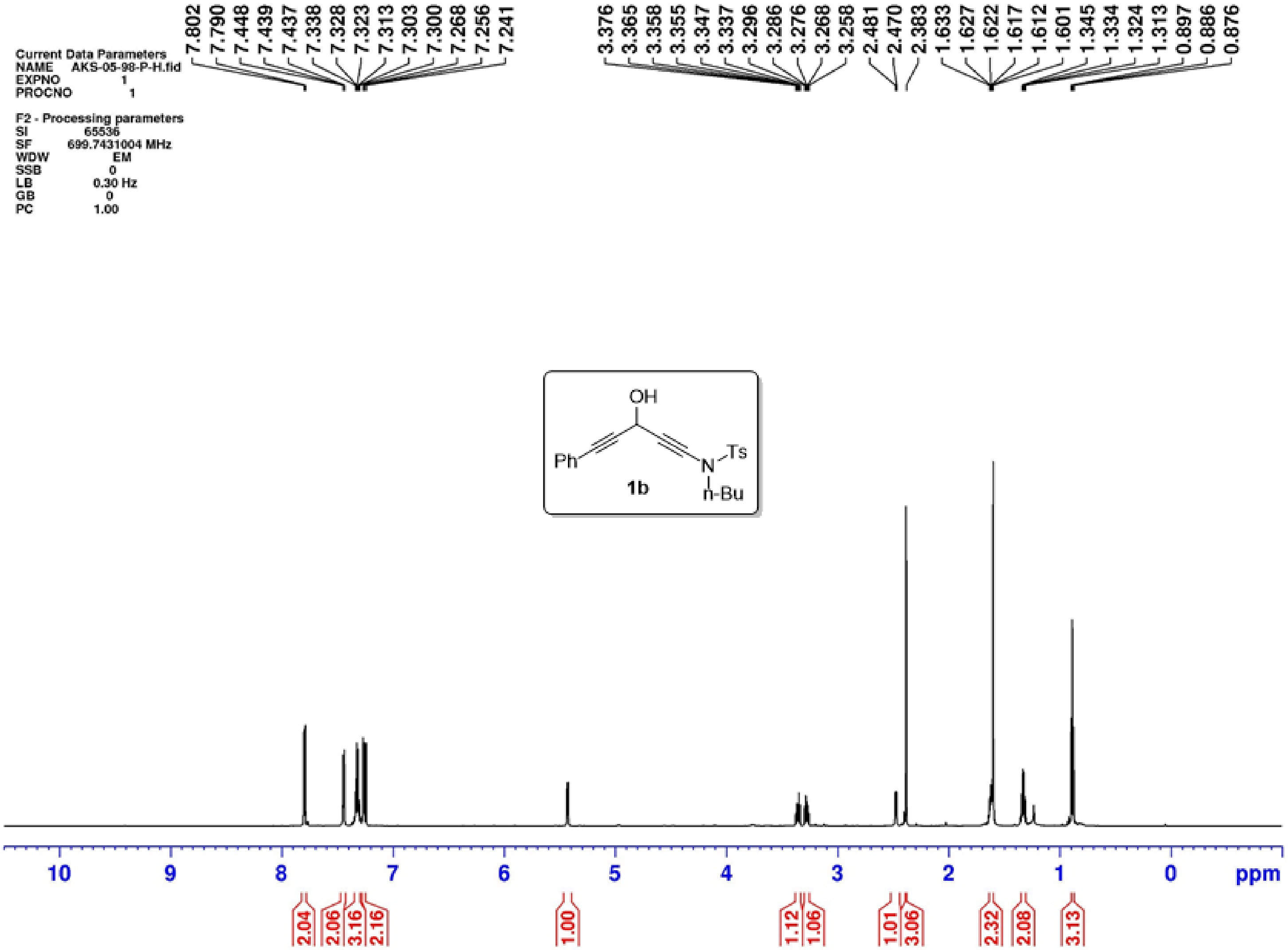

$^{13}C\{1H\}$ and DEPT NMR ($CDCl_3$, 175 MHz)

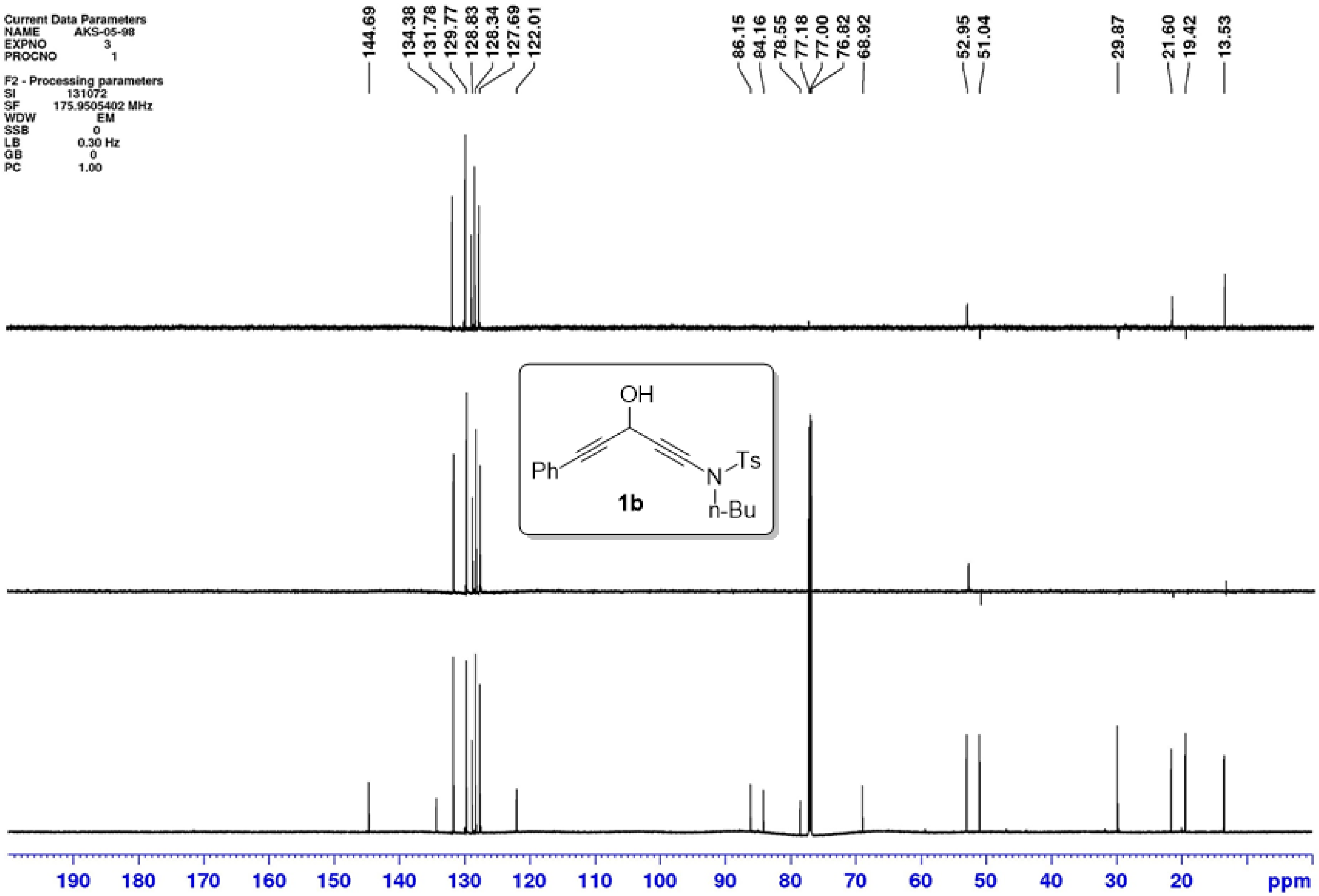

^{1}H NMR ($CDCl_3$, 700 MHz)

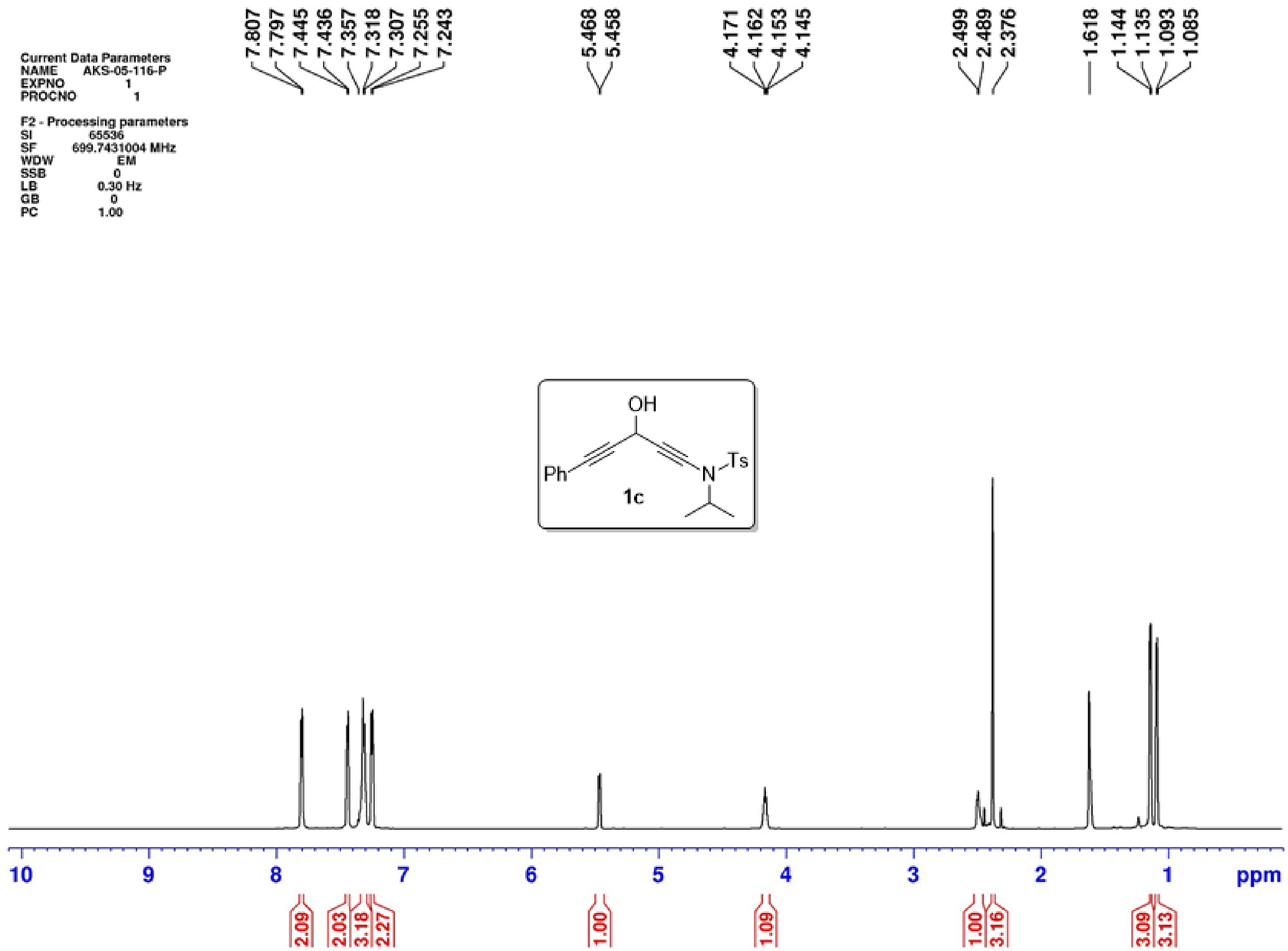

$^{13}C\{1H\}$ and DEPT NMR ($CDCl_3$, 175 MHz)

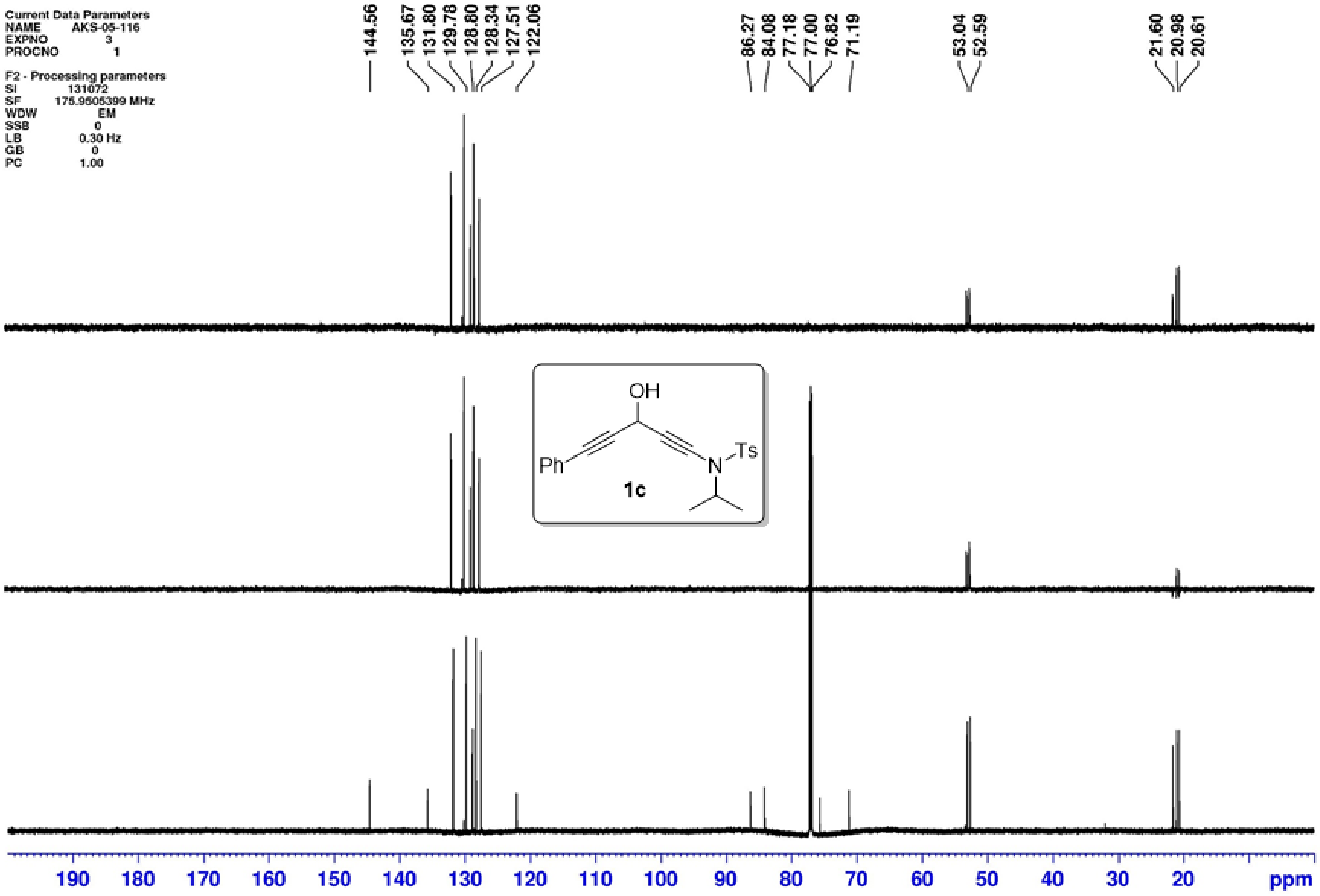

^{1}H NMR ($CDCl_3$, 700 MHz)

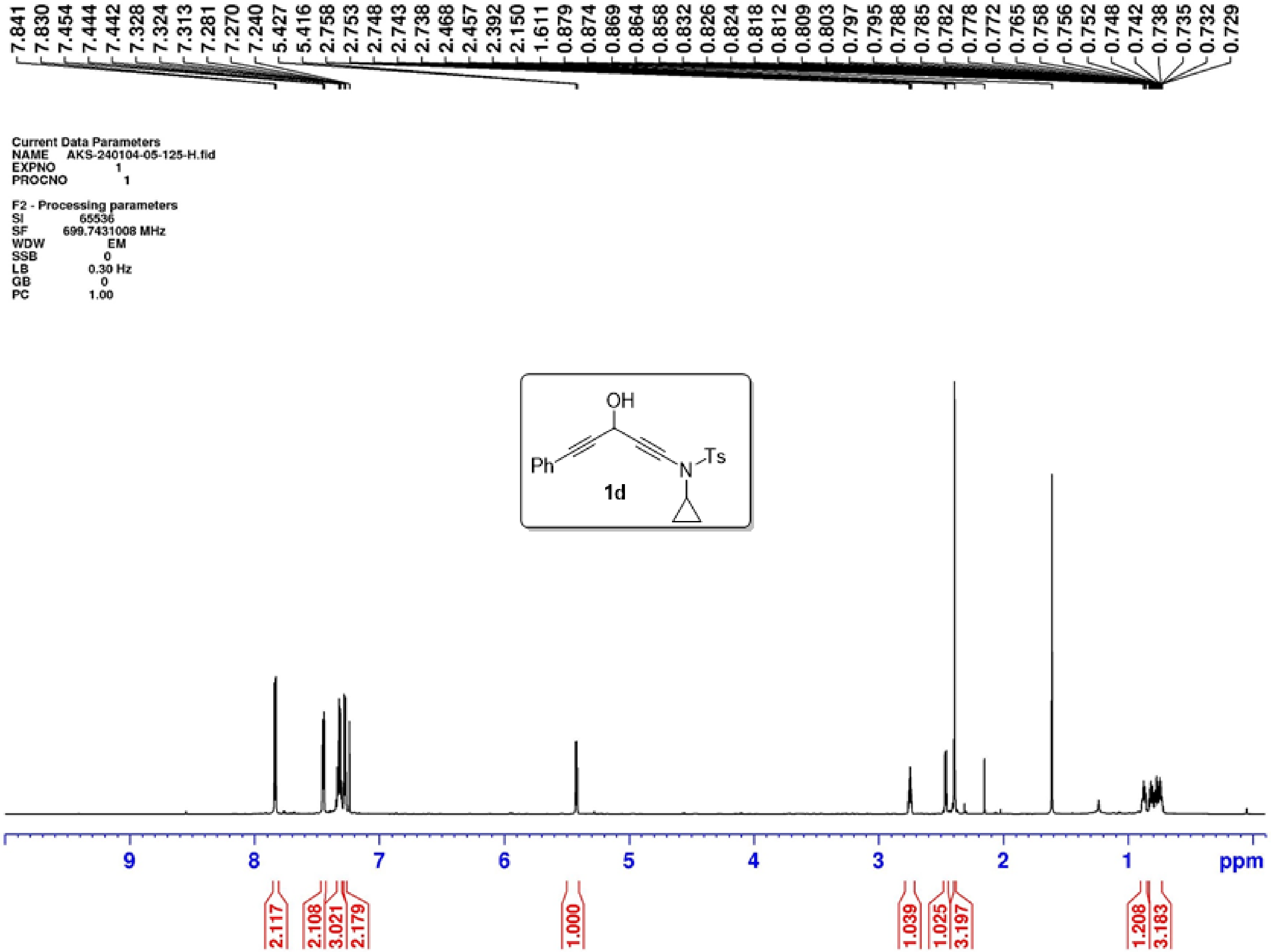

$^{13}C\{1H\}$ and DEPT NMR ($CDCl_3$, 175 MHz)

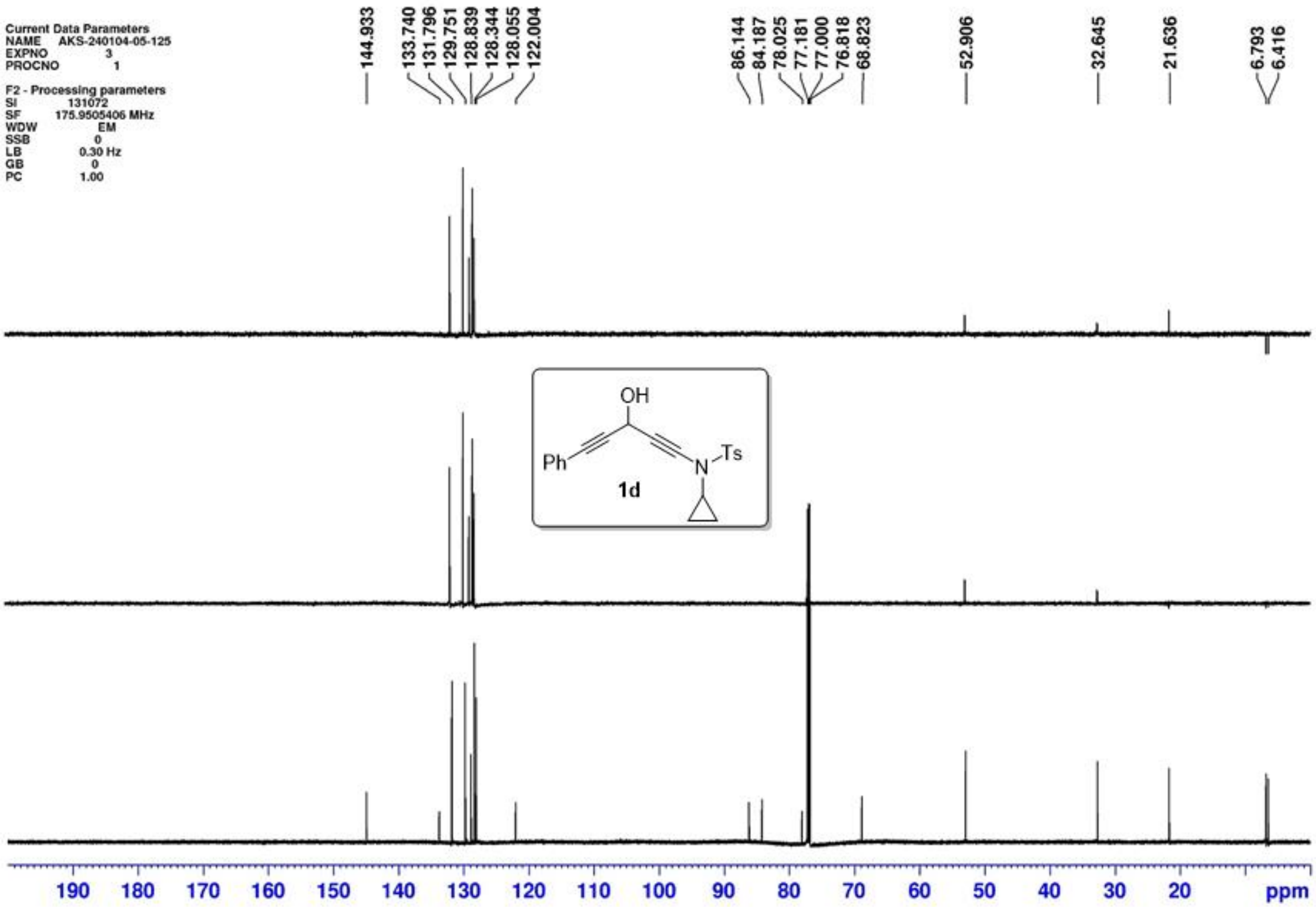

^{1}H NMR ($CDCl_3$, 700 MHz)

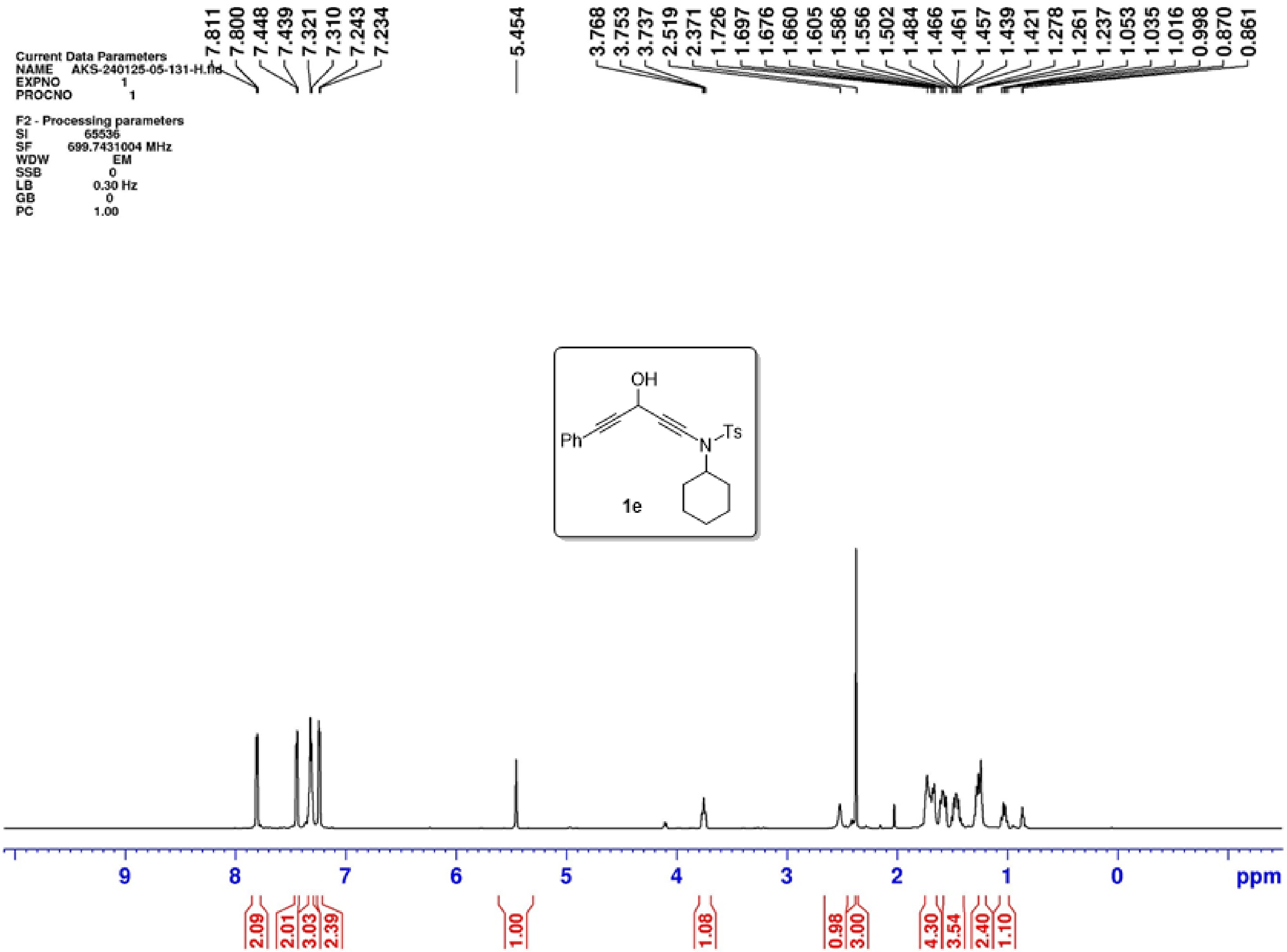

$^{13}C\{1H\}$ and DEPT NMR ($CDCl_3$, 175 MHz)

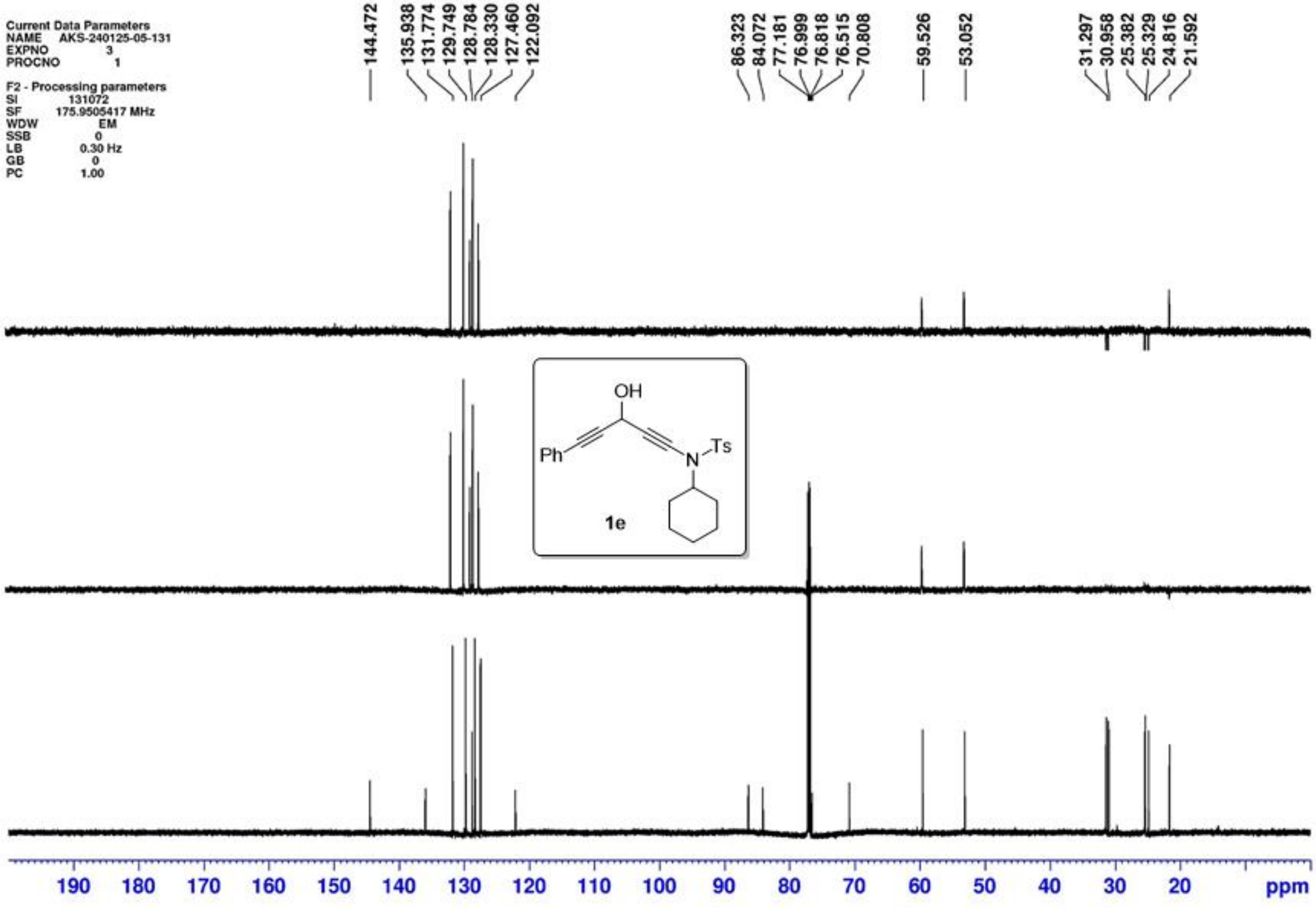

^{1}H NMR ($CDCl_3$, 700 MHz)

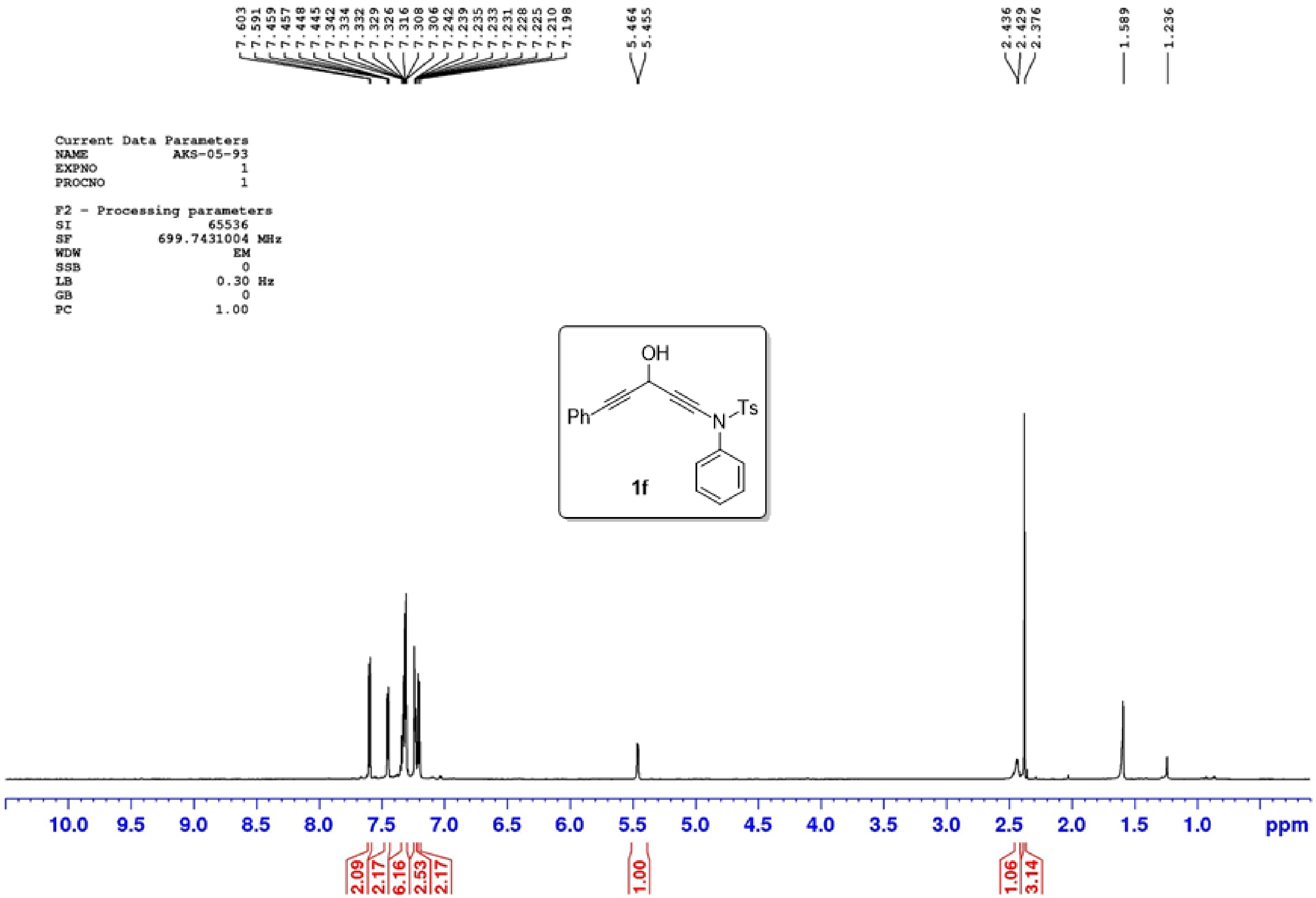

$^{13}C\{1H\}$ and DEPT NMR ($CDCl_3$, 175 MHz)

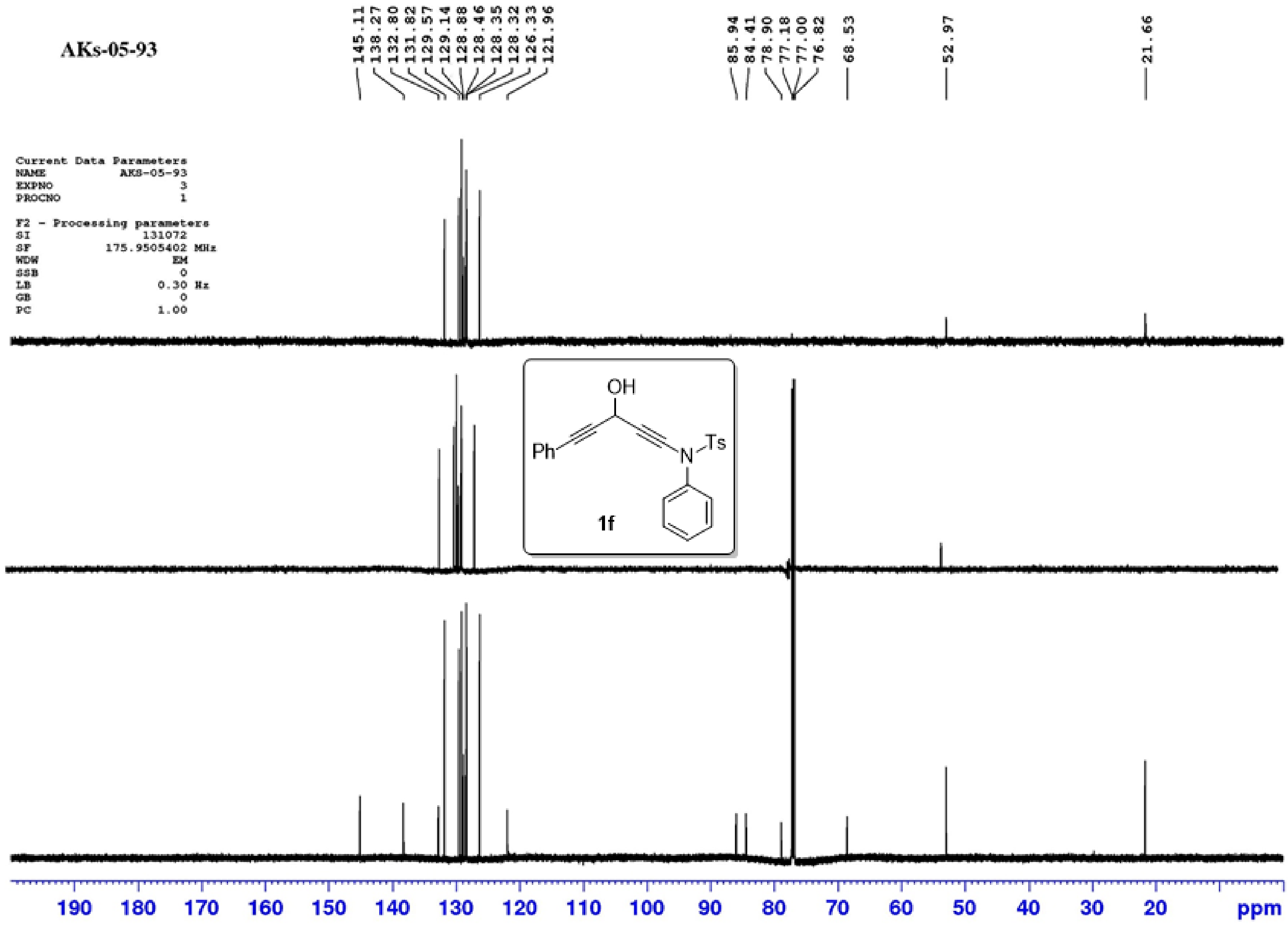

^{1}H NMR ($CDCl_3$, 700 MHz)

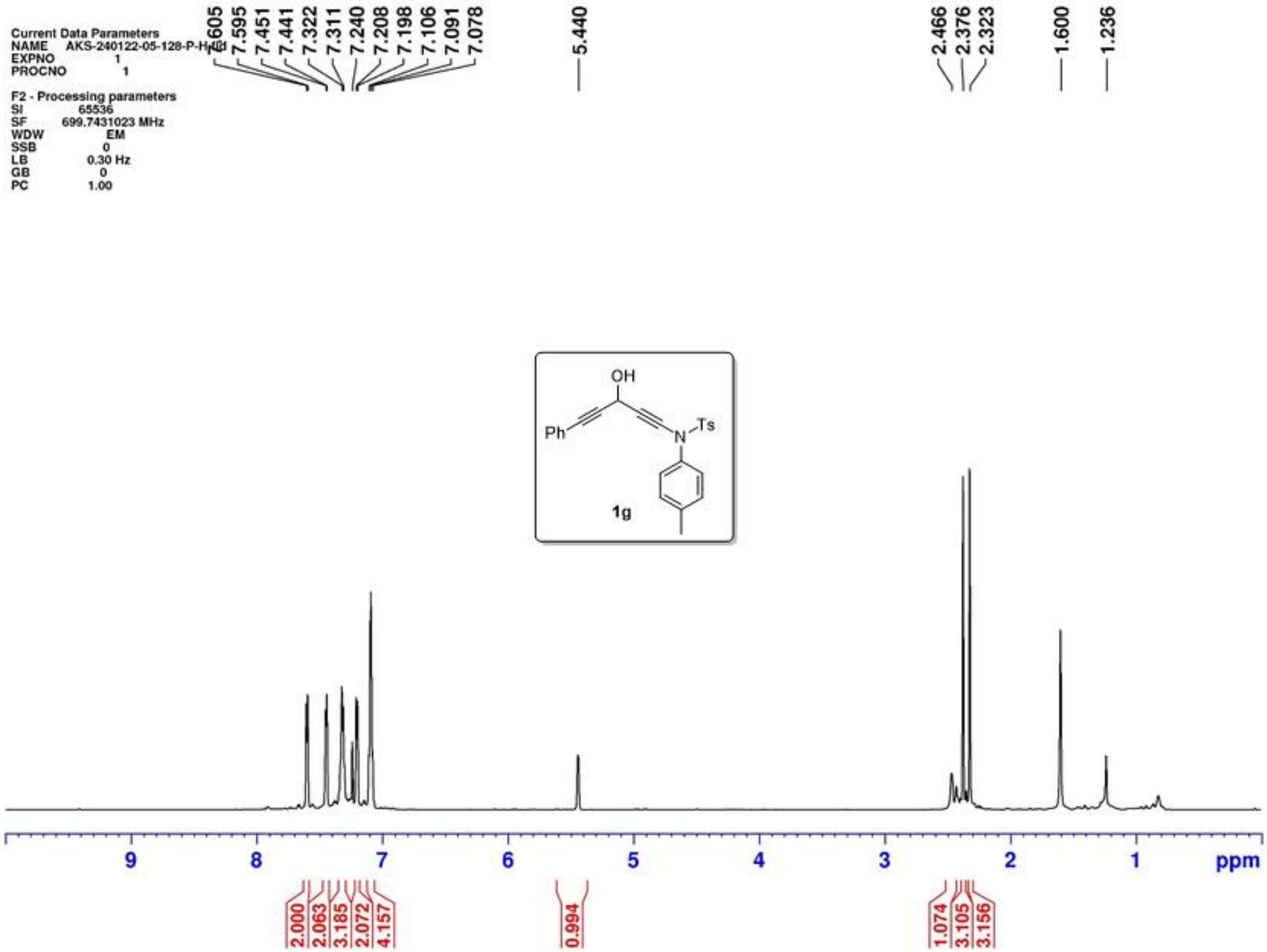

$^{13}C\{1H\}$ and DEPT NMR ($CDCl_3$, 175 MHz)

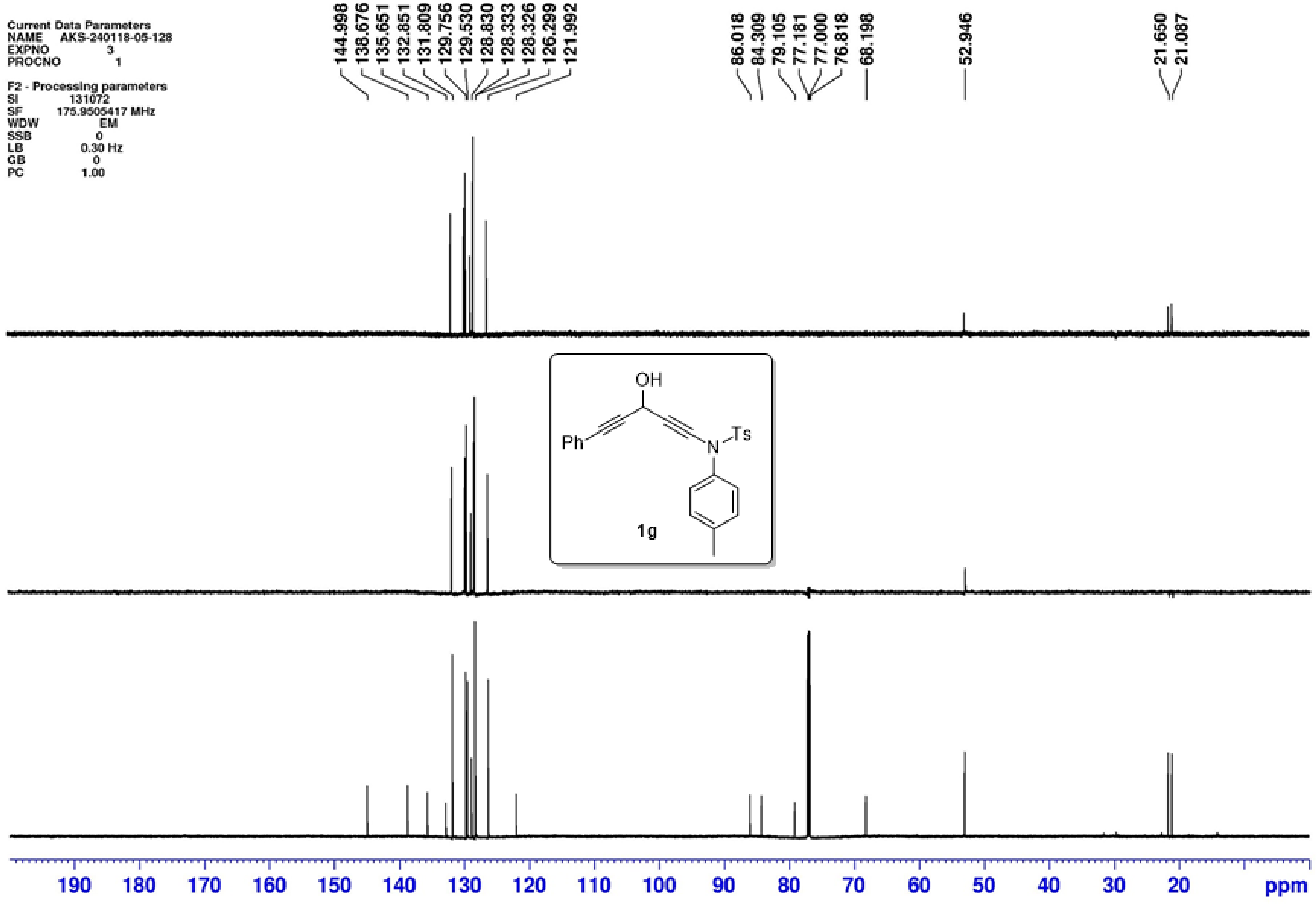

^{1}H NMR (CDCl$_3$, 700 MHz)

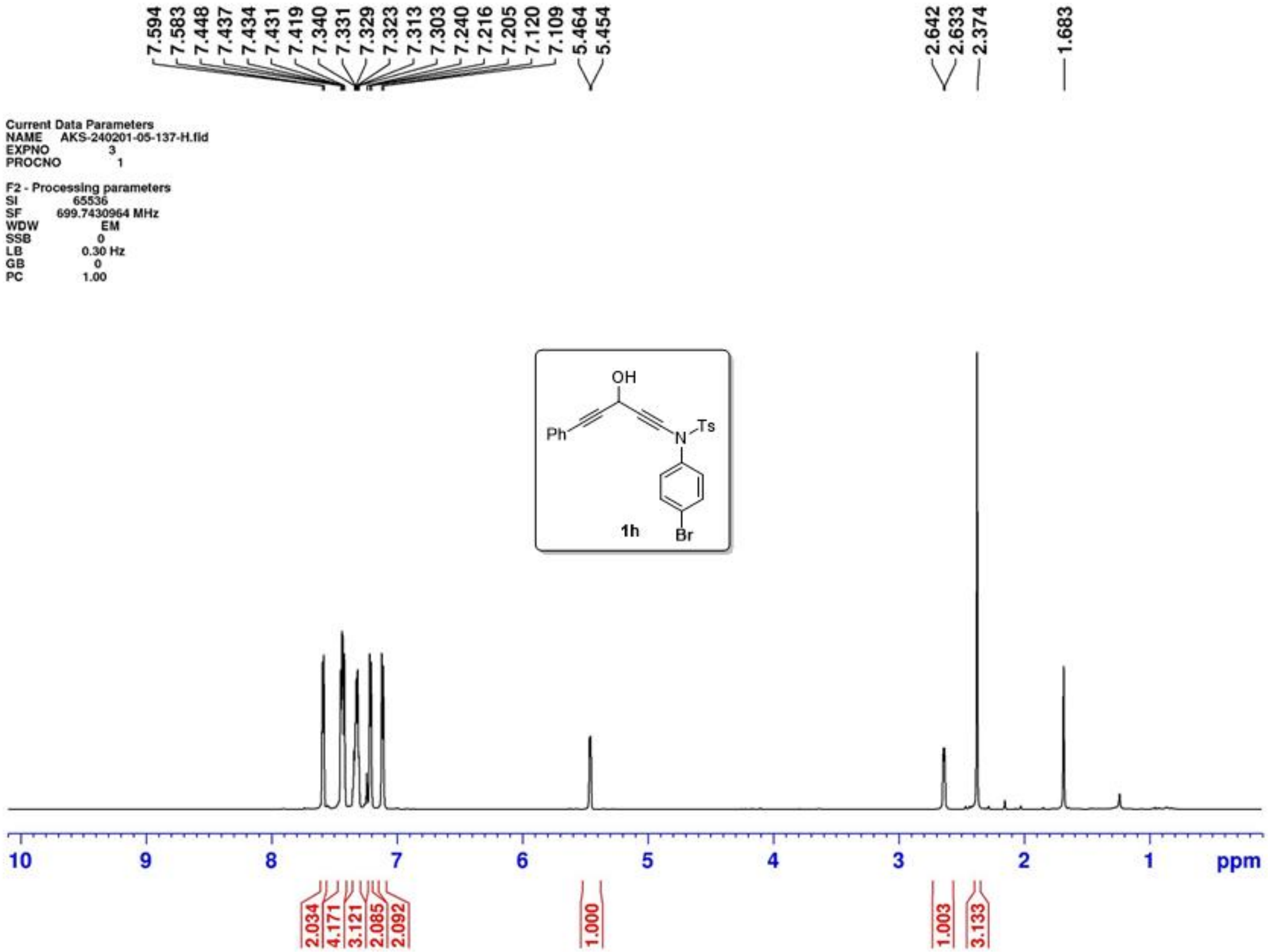

$^{13}C\{1H\}$ and DEPT NMR ($CDCl_3$, 175 MHz)

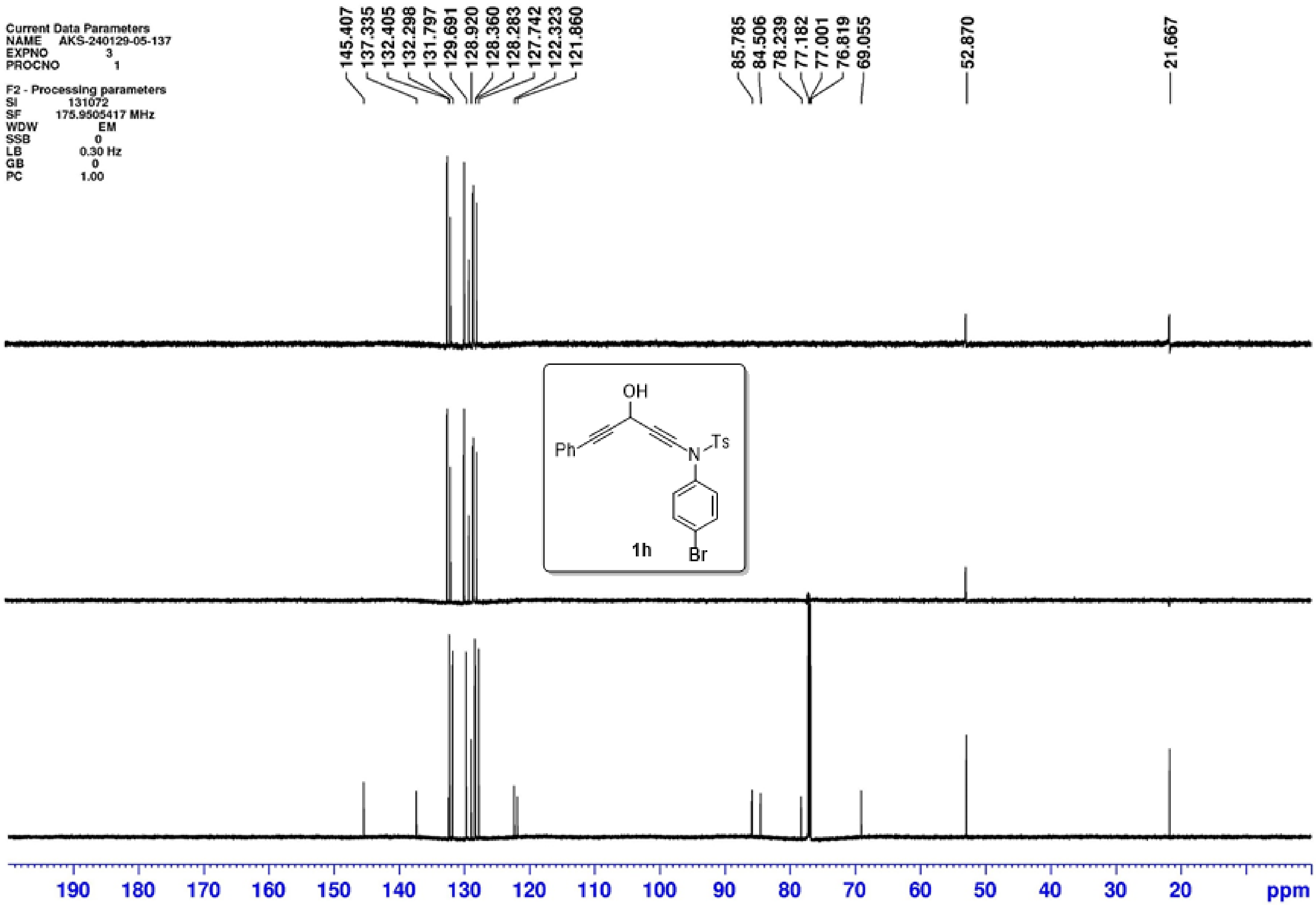

^{1}H NMR ($CDCl_3$, 700 MHz)

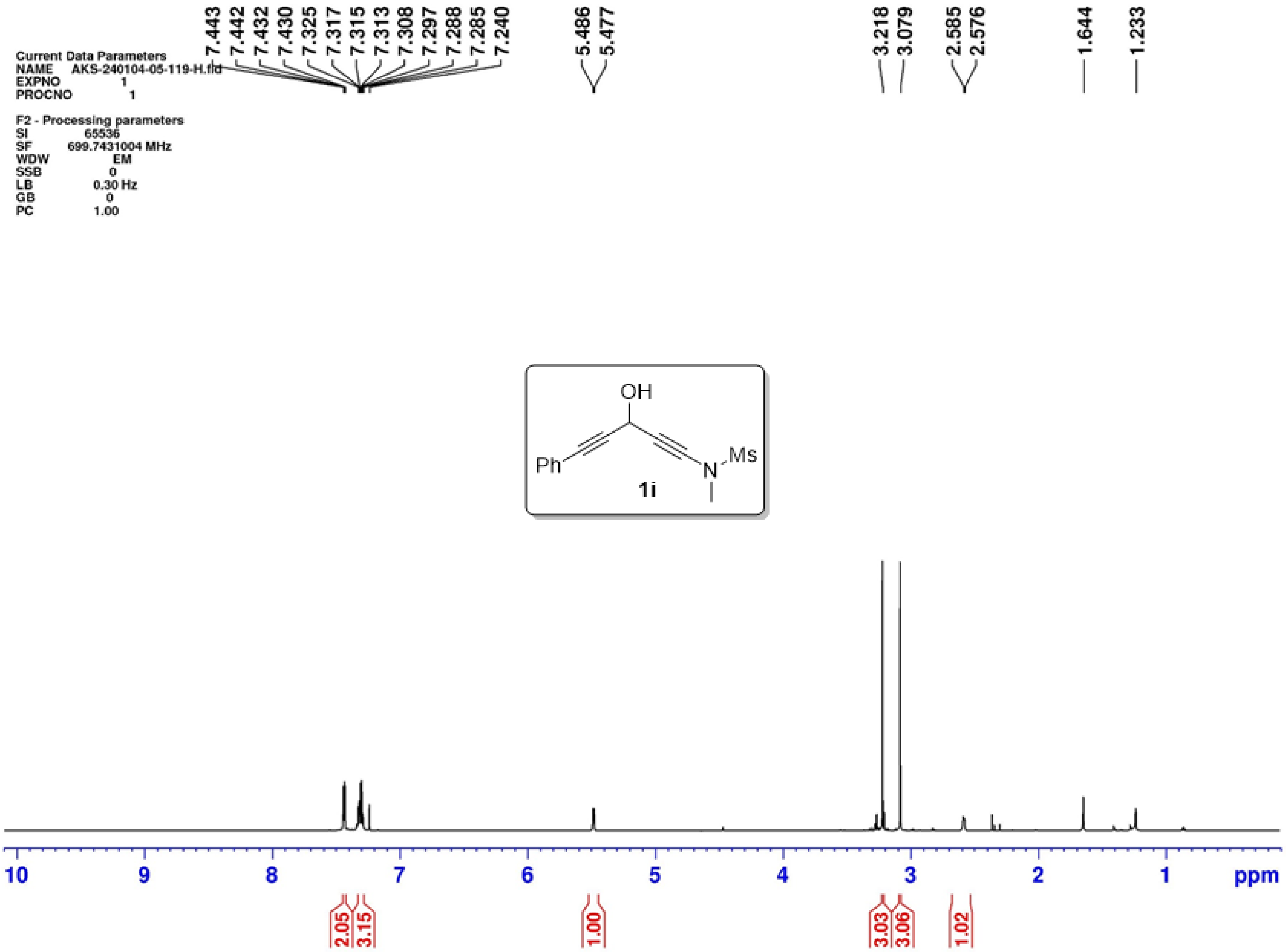

$^{13}C\{1H\}$ and DEPT NMR ($CDCl_3$, 175 MHz)

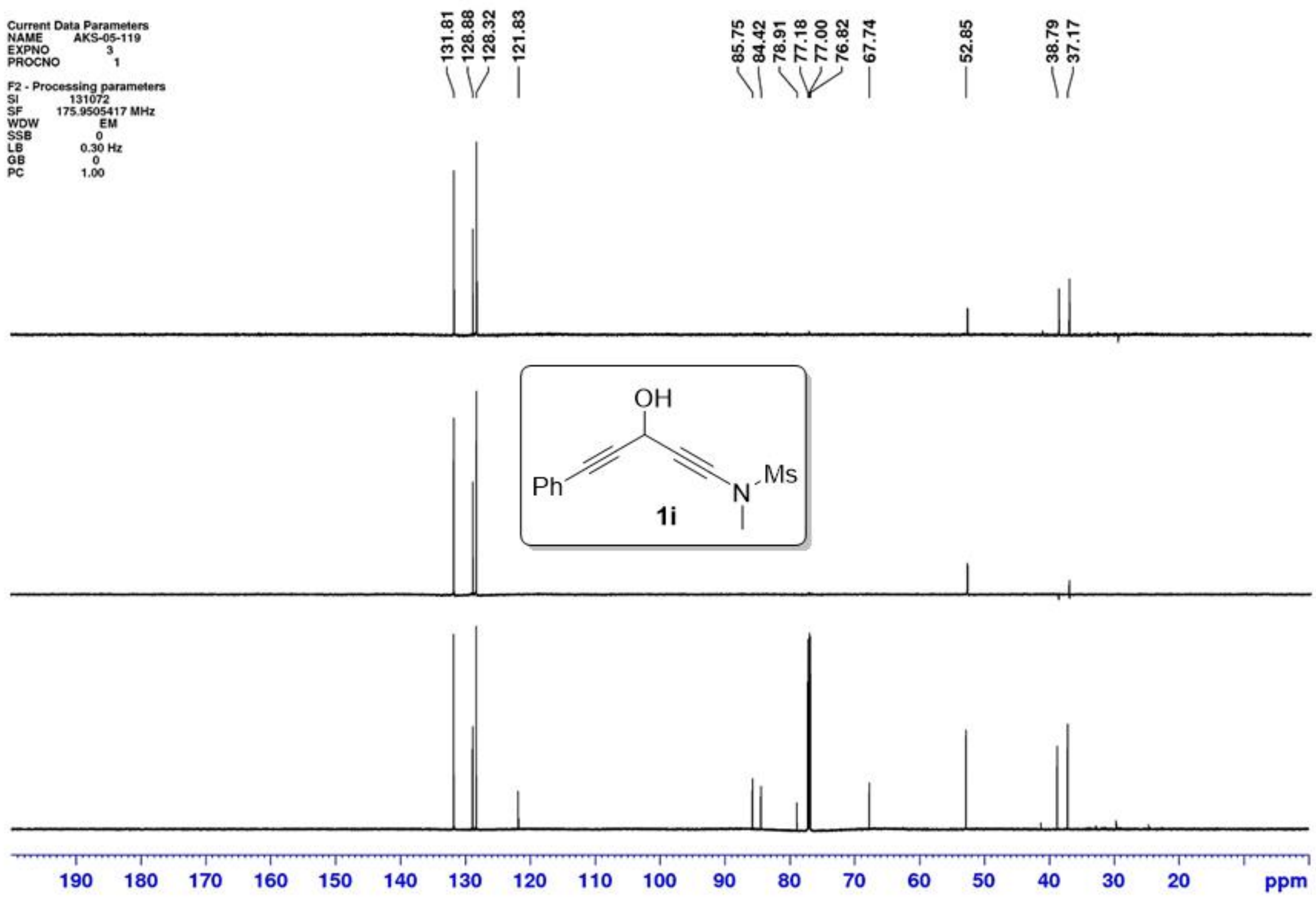

^{1}H NMR ($CDCl_3$, 700 MHz)

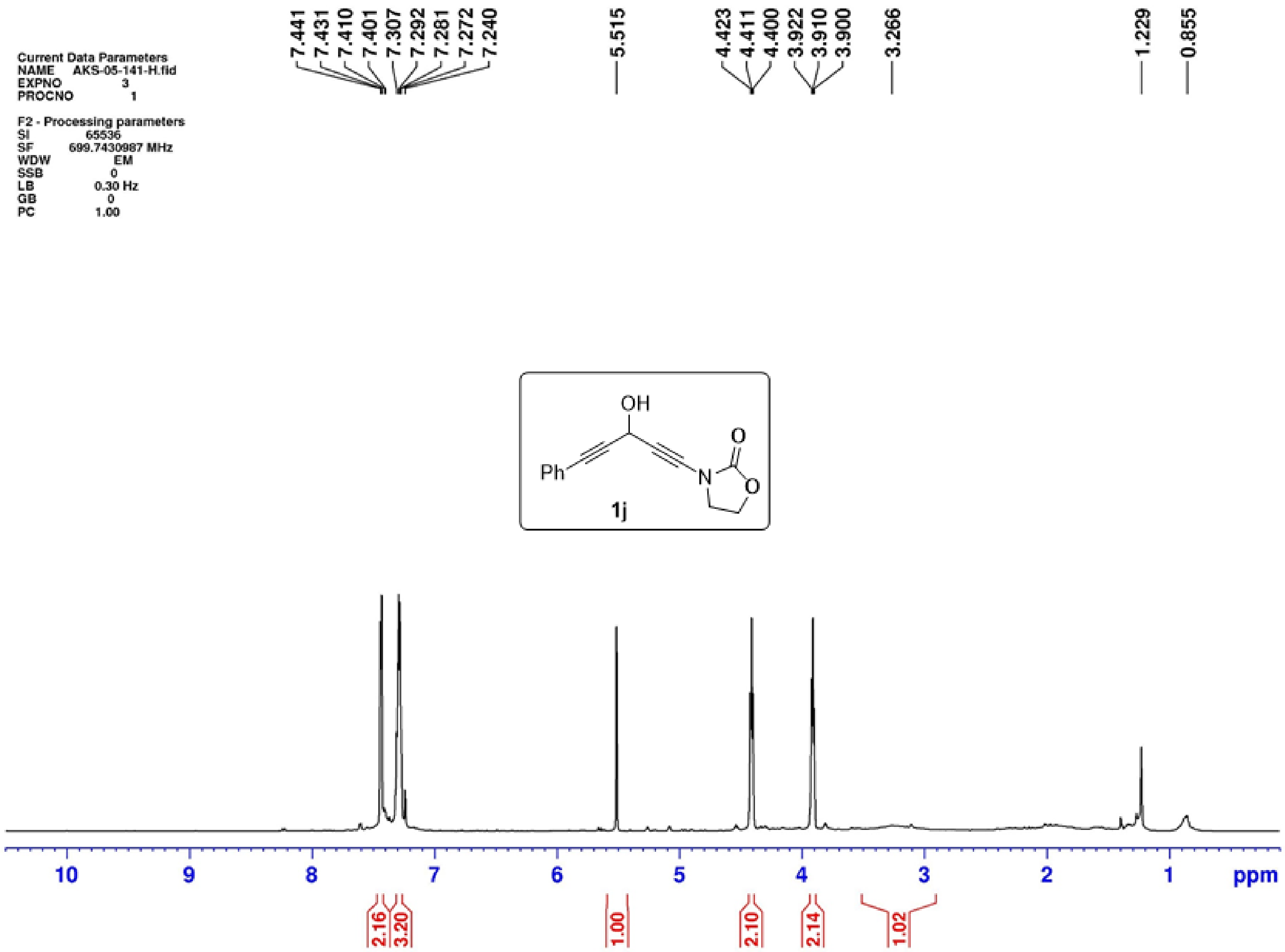

^{13}C{1H} and DEPT NMR ($CDCl_3$, 175 MHz)

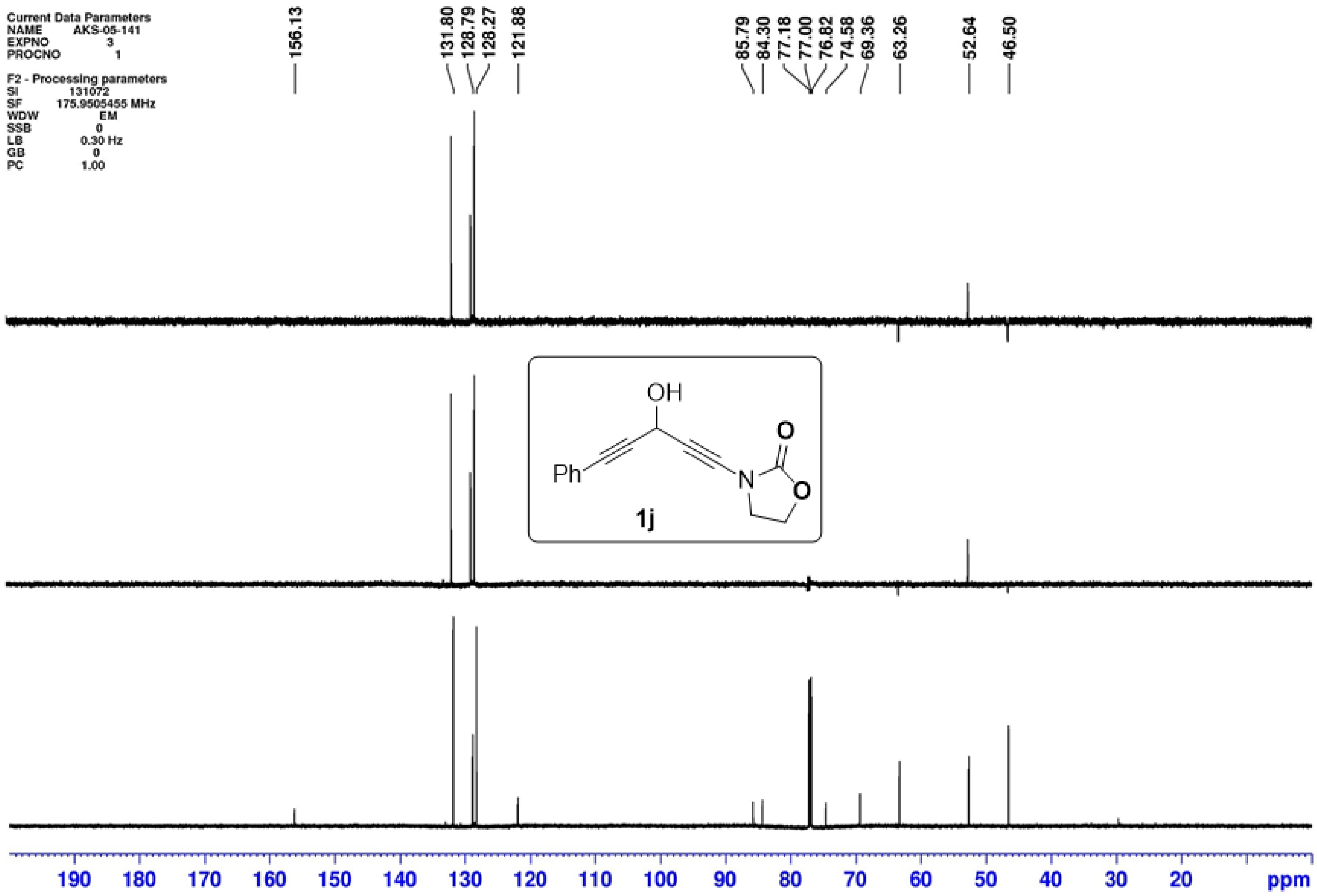

^{1}H NMR ($CDCl_3$, 700 MHz)

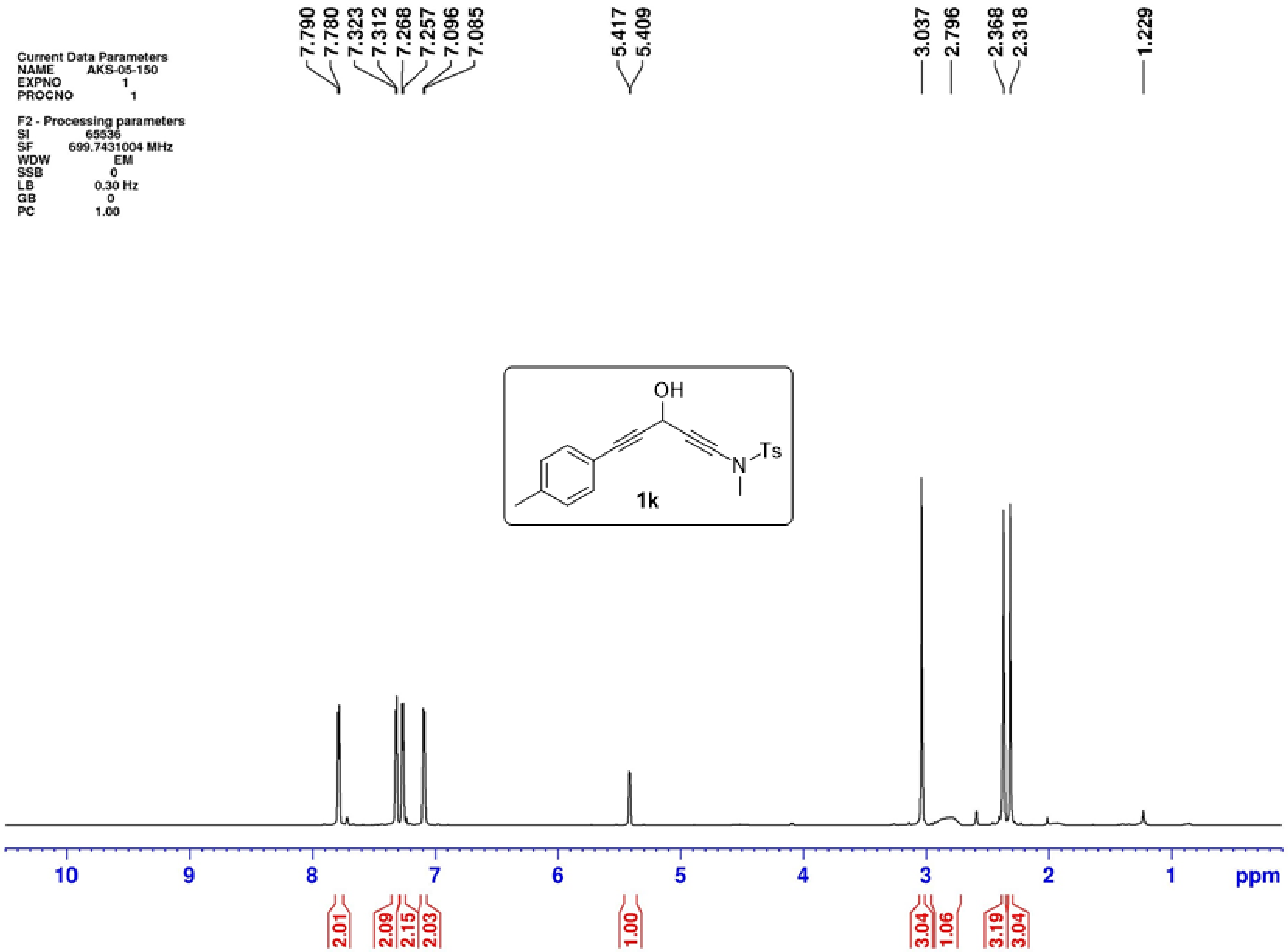

$^{13}C\{1H\}$ and DEPT NMR ($CDCl_3$, 175 MHz)

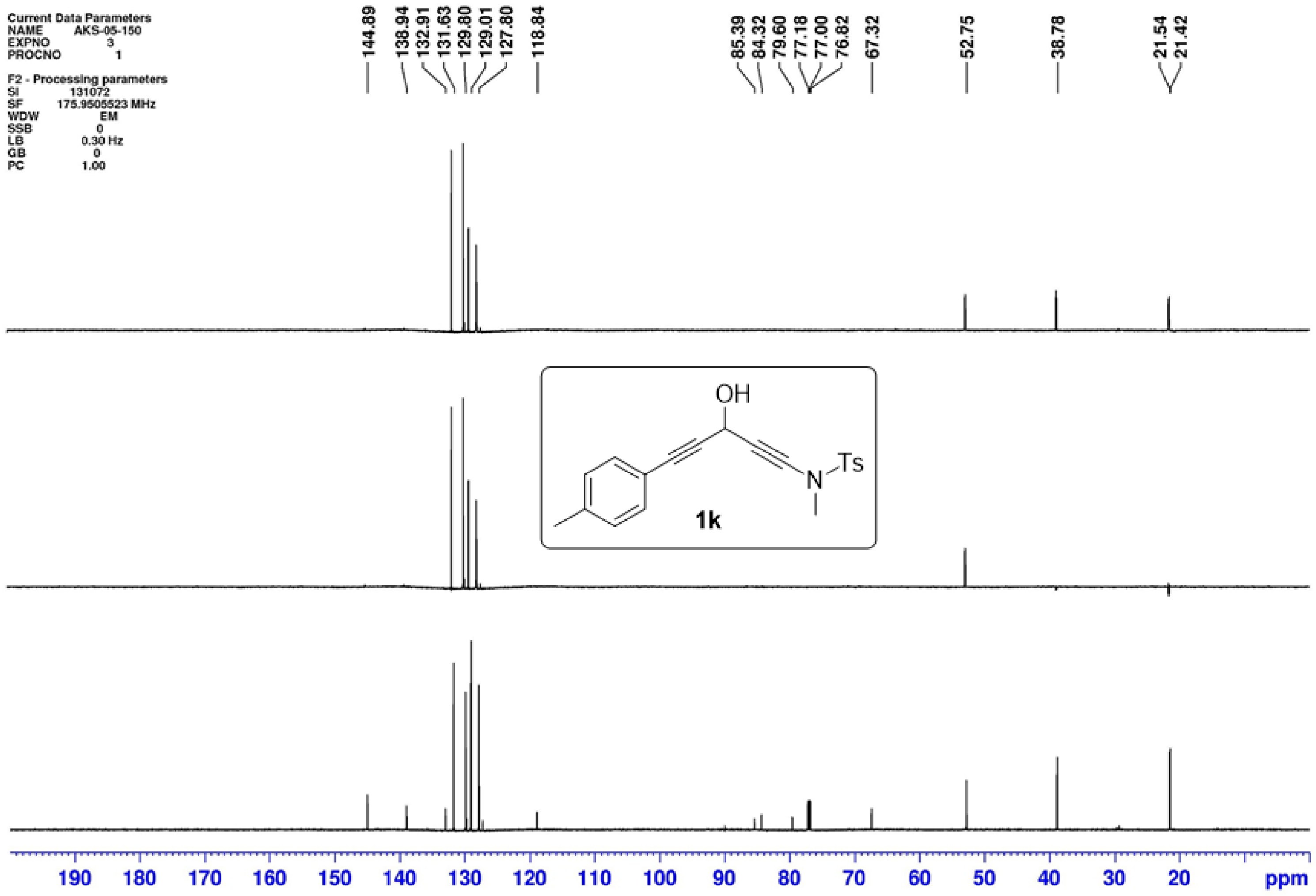

^{1}H NMR ($CDCl_3$, 700 MHz)

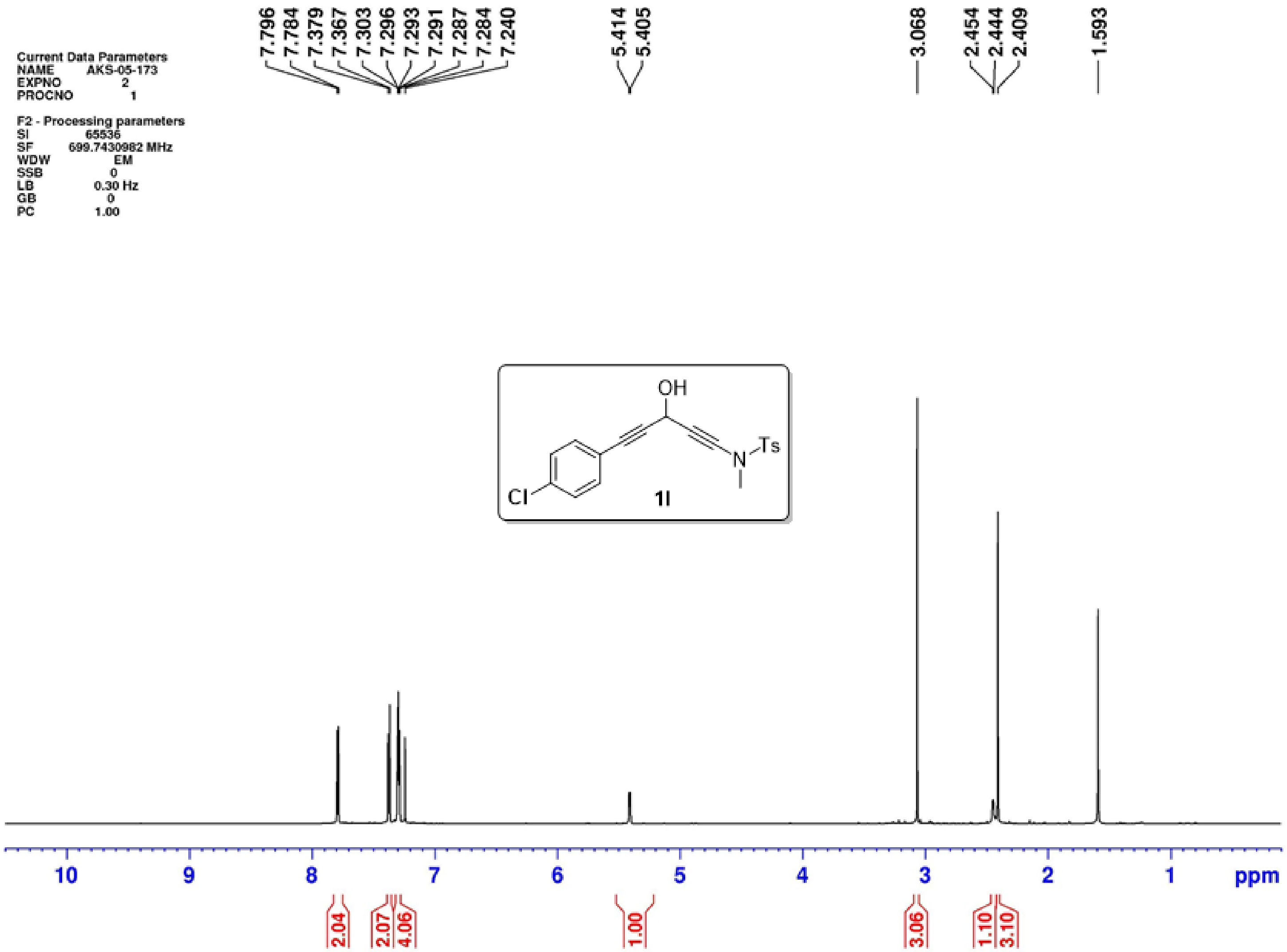

$^{13}C\{1H\}$ and DEPT NMR ($CDCl_3$, 175 MHz)

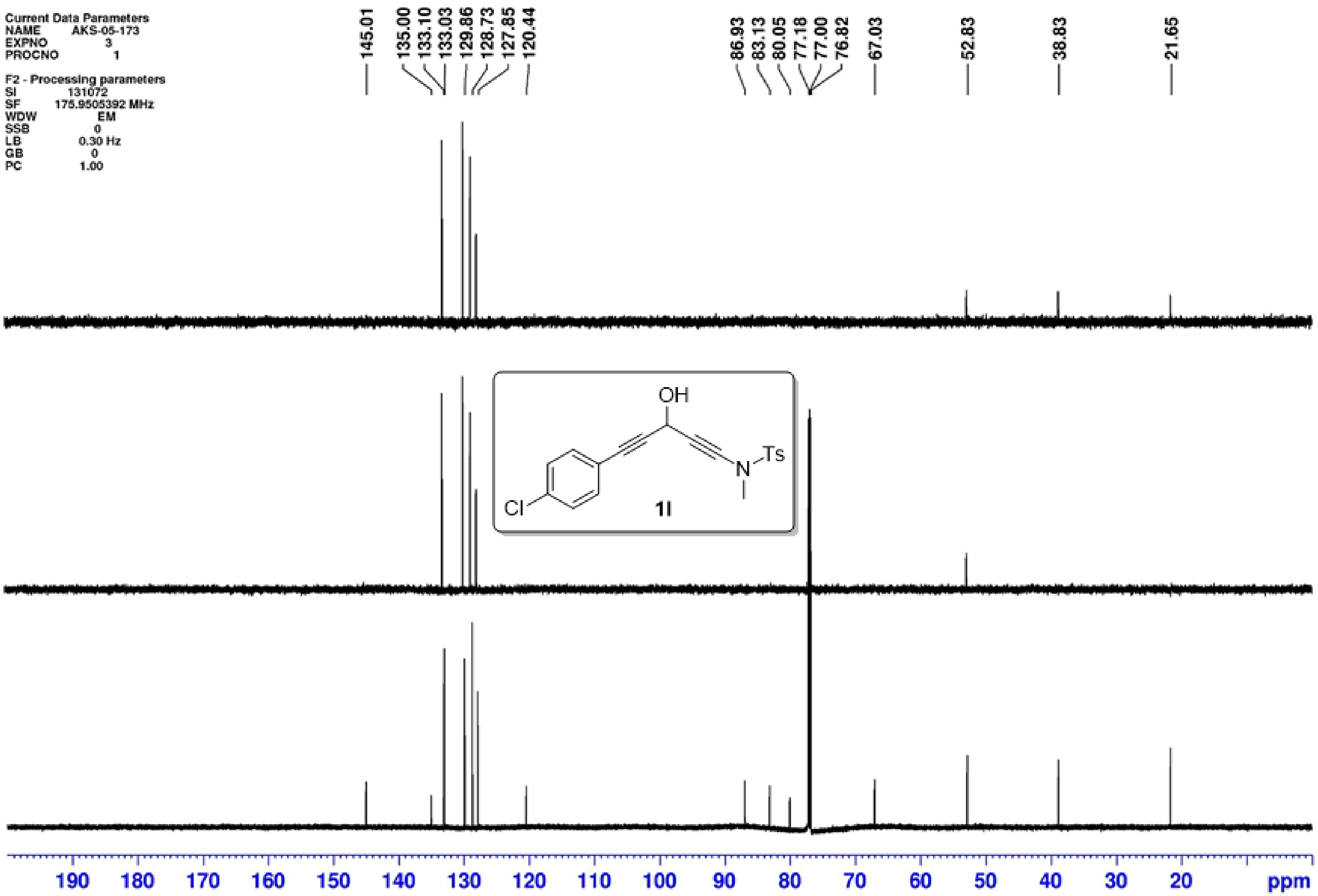

^{1}H NMR ($CDCl_3$, 400 MHz)

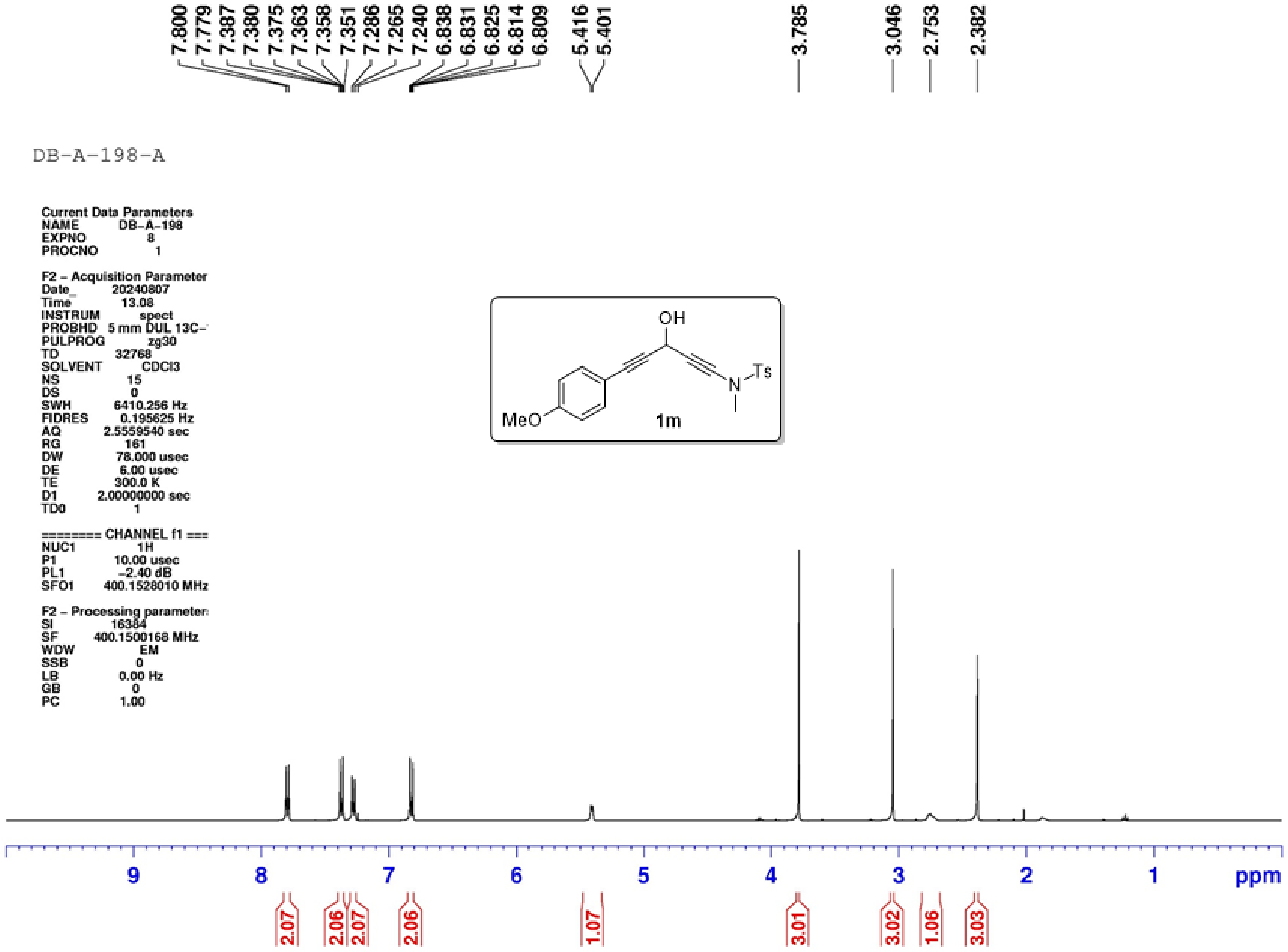

$^{13}C\{1H\}$ NMR ($CDCl_3$, 100 MHz)

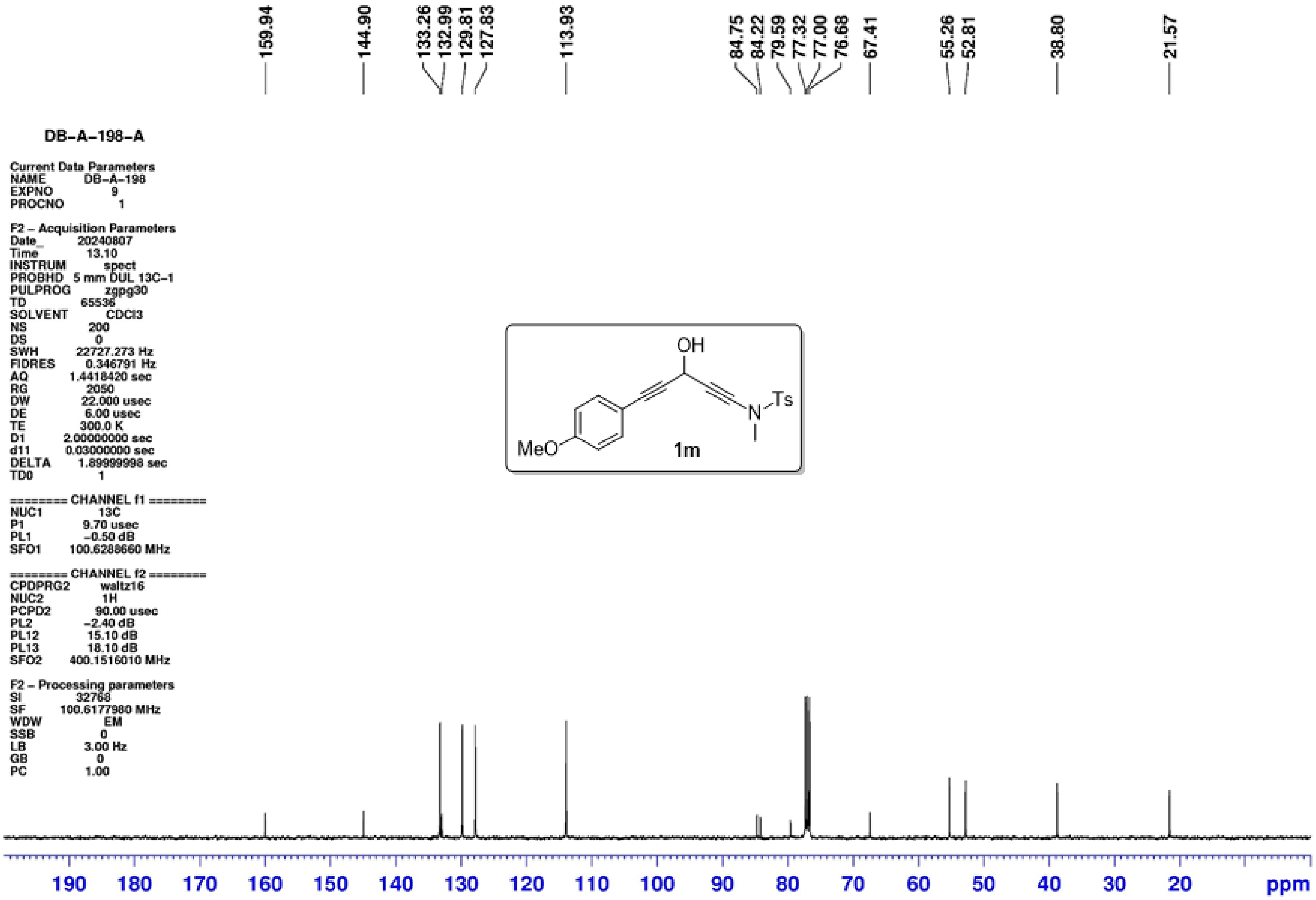

^{1}H NMR ($CDCl_3$, 400 MHz)

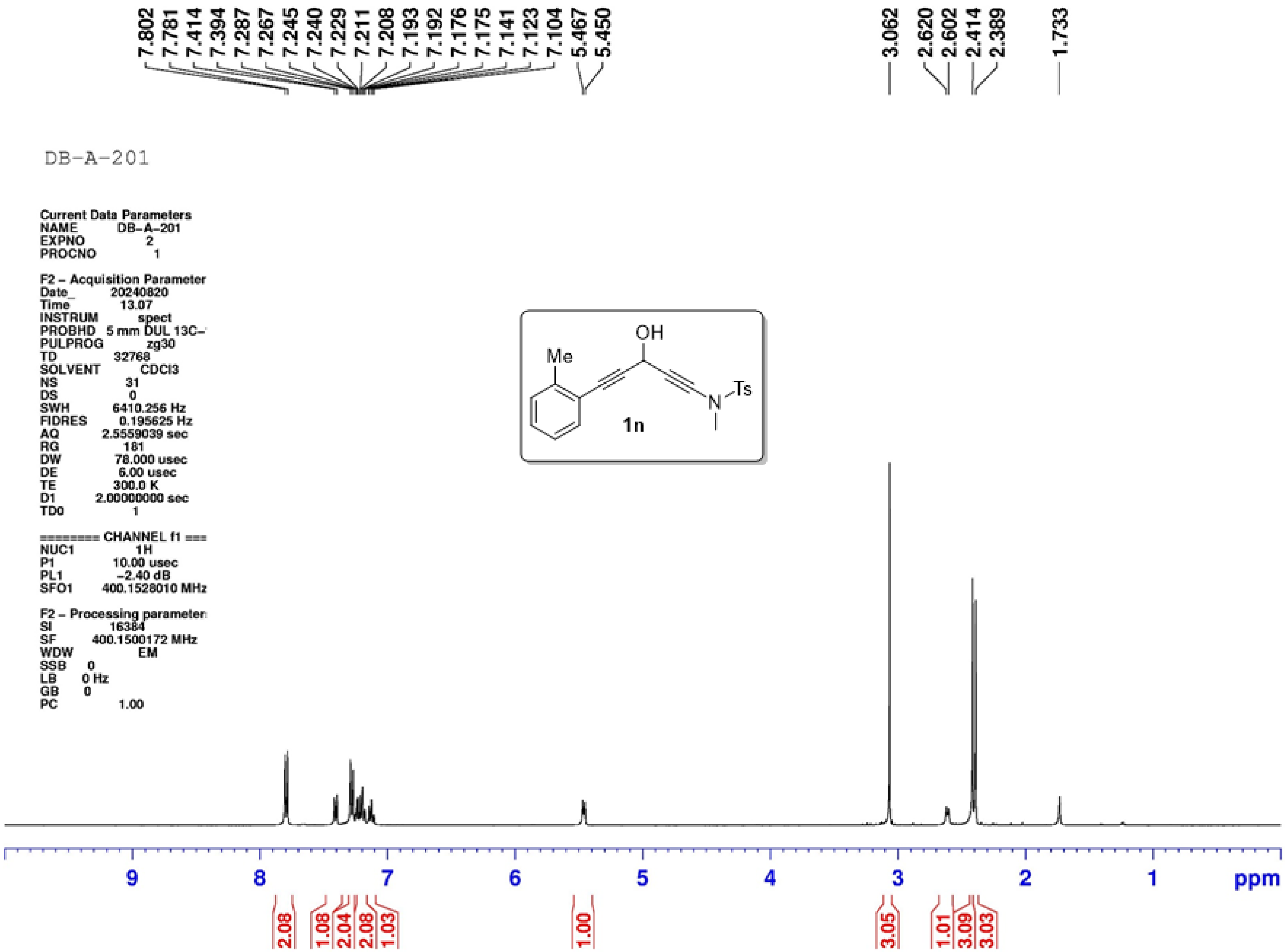

$^{13}C\{1H\}$ NMR ($CDCl_3$, 100 MHz)

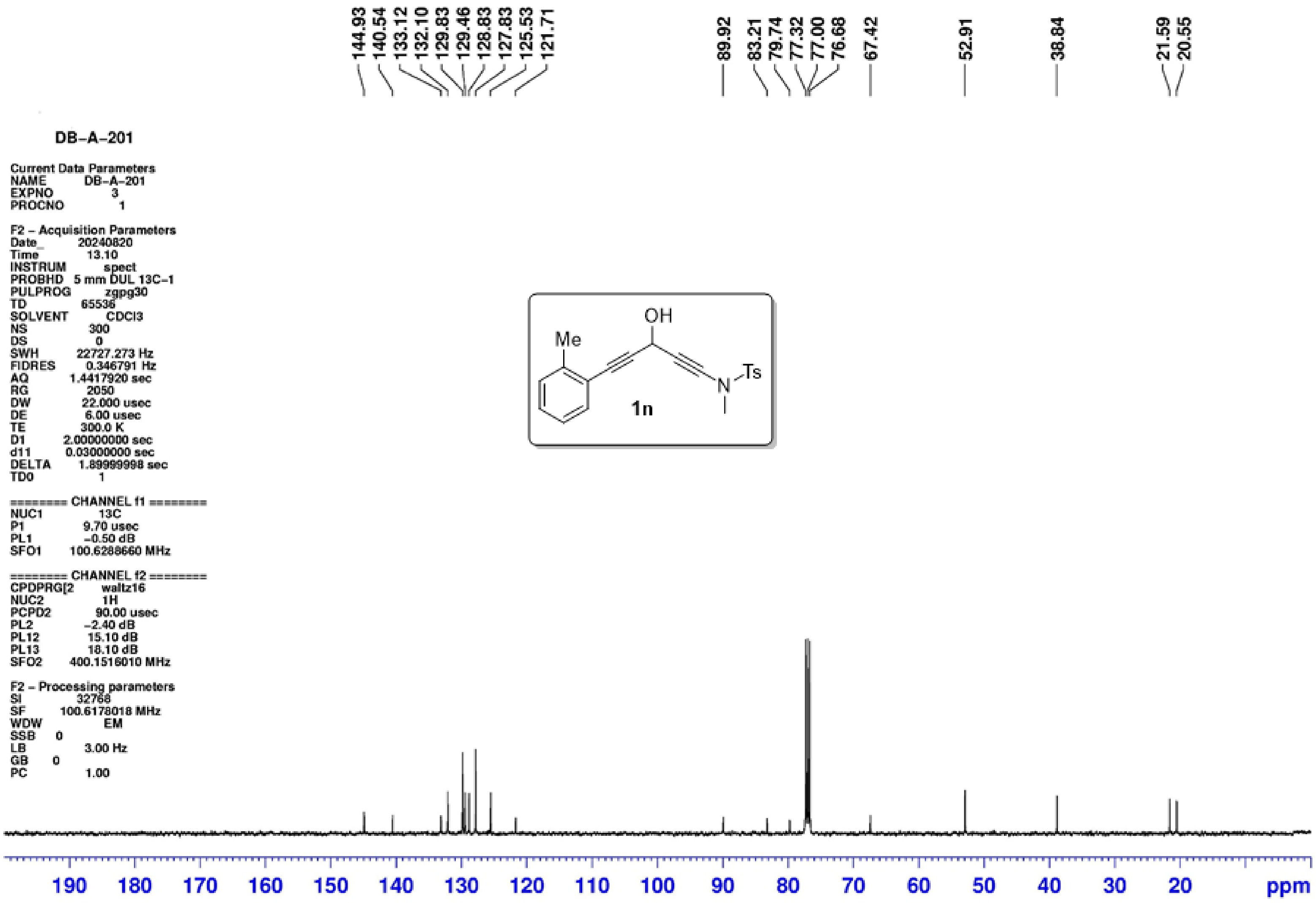

^{1}H NMR (CDCl$_3$, 700 MHz)

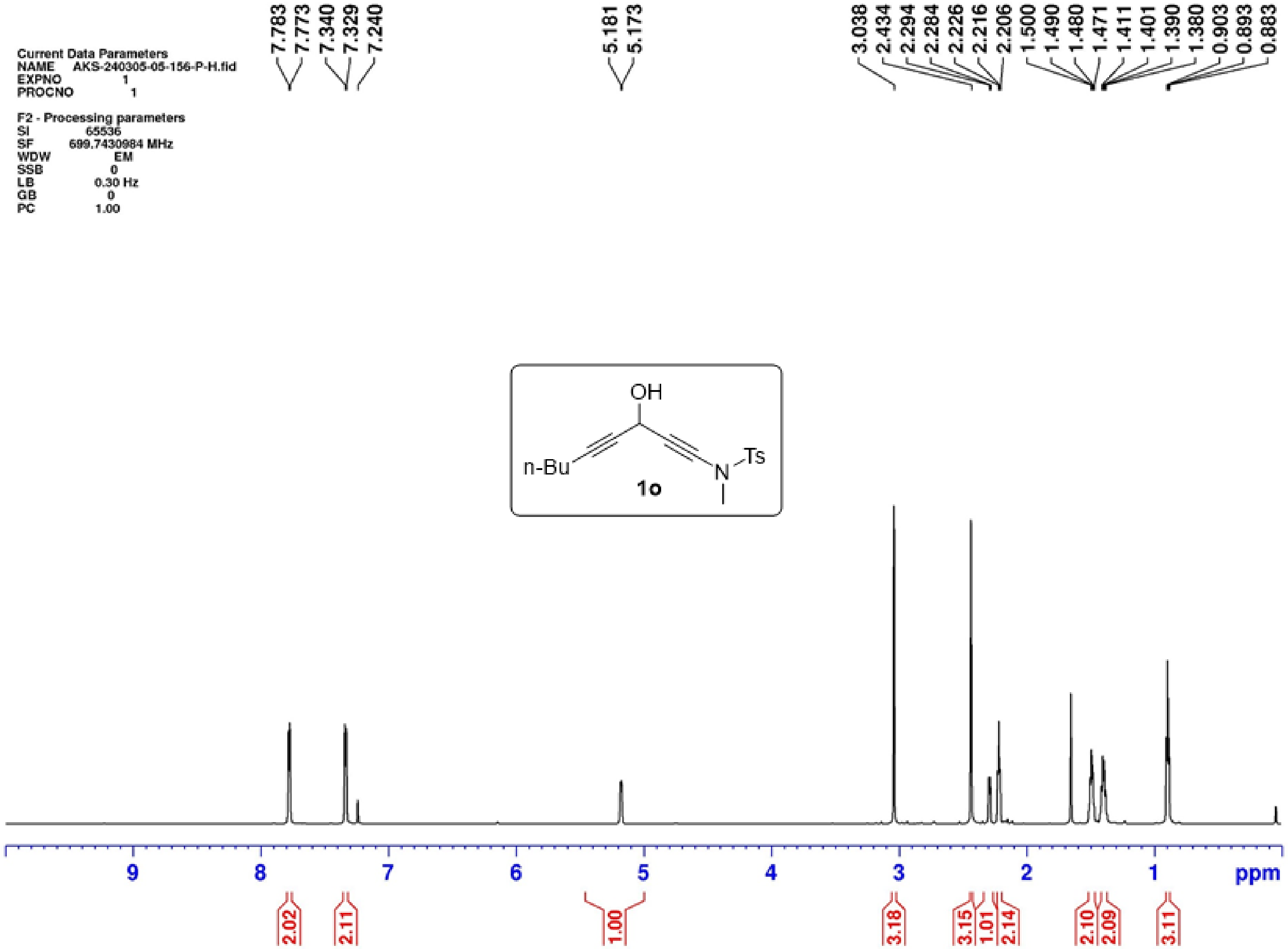

$^{13}C\{1H\}$ and DEPT NMR ($CDCl_3$, 175 MHz)

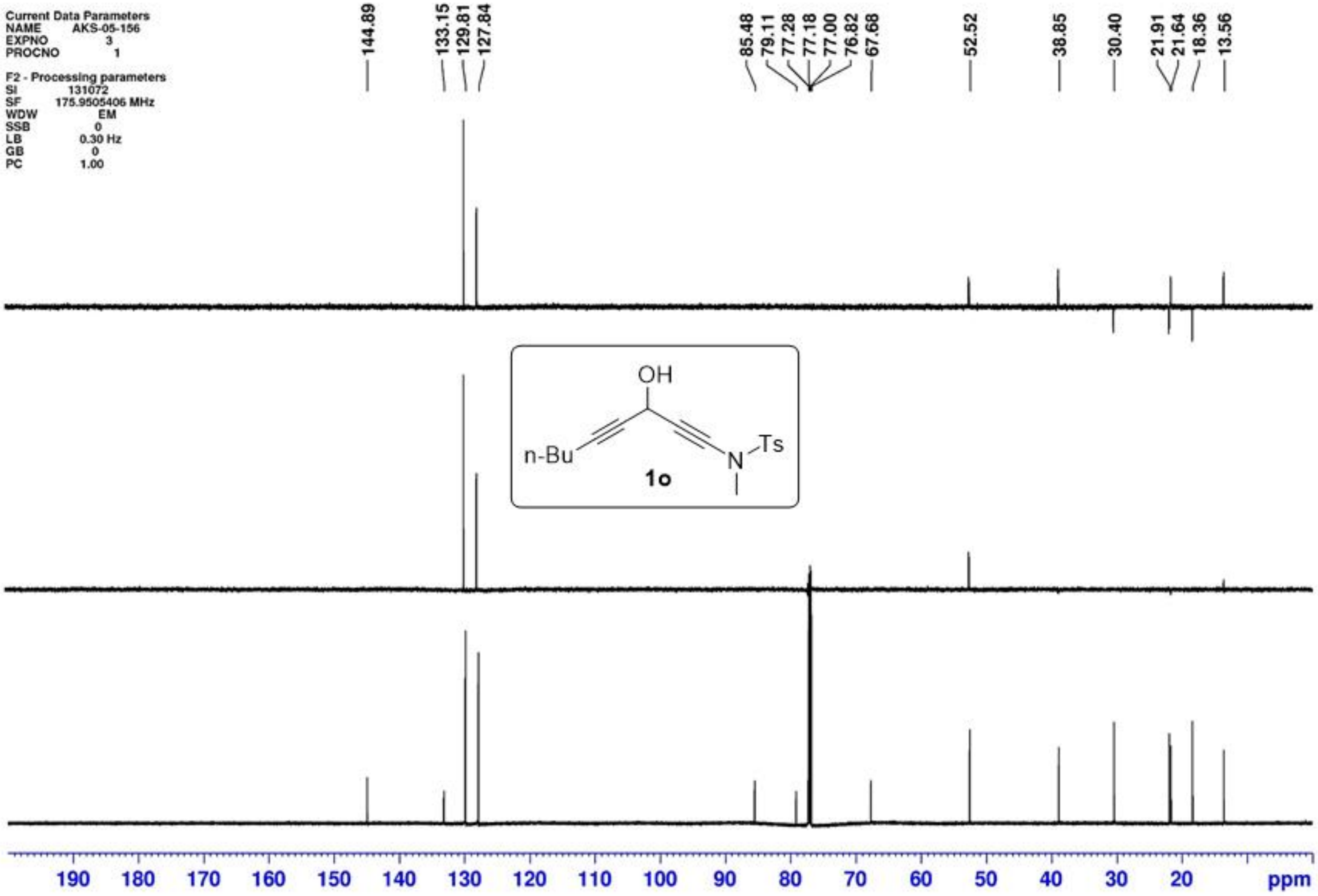

^{1}H NMR ($CDCl_3$, 400 MHz)

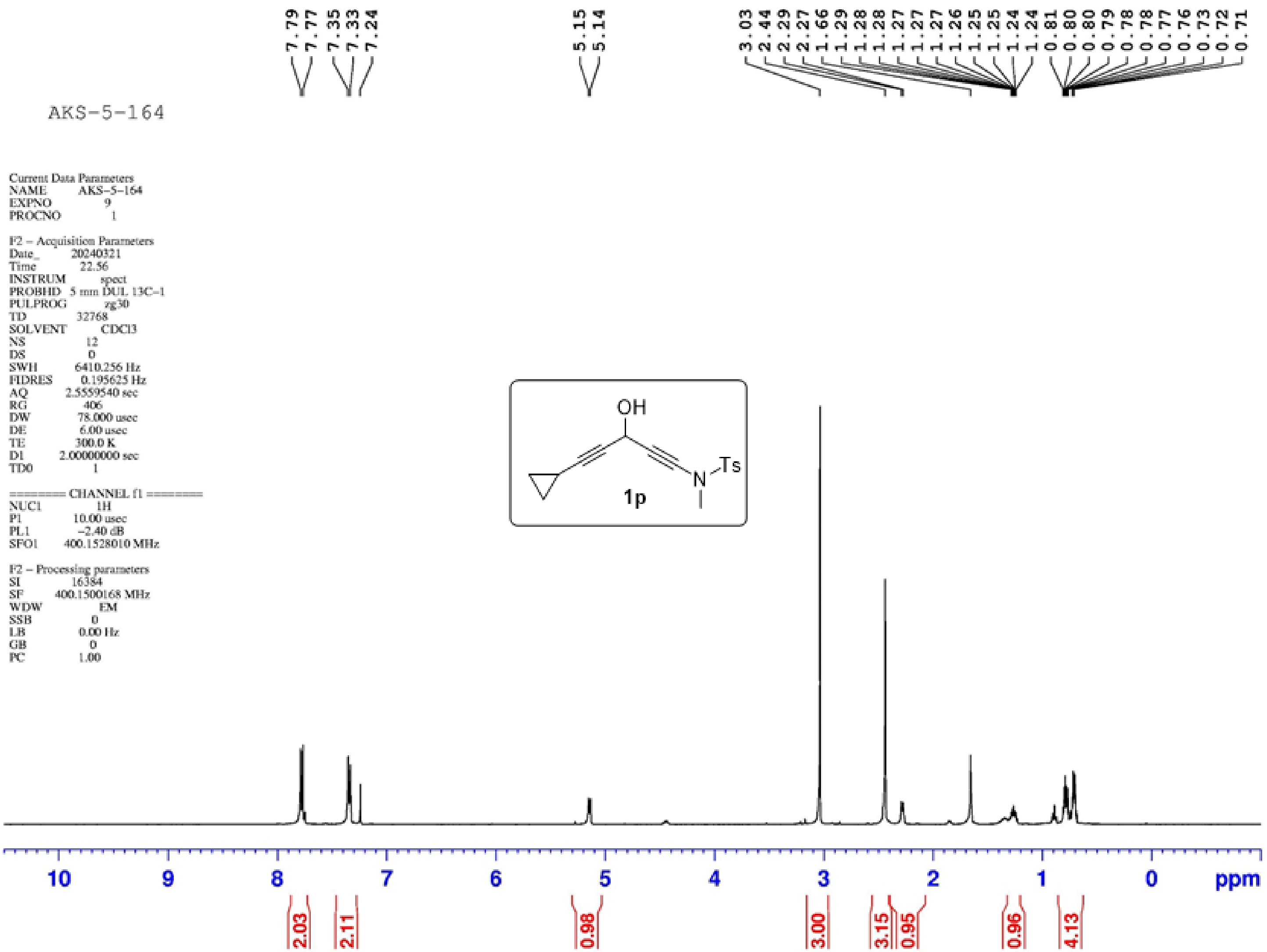

$^{13}C\{1H\}$ and DEPT NMR ($CDCl_3$, 175 MHz)

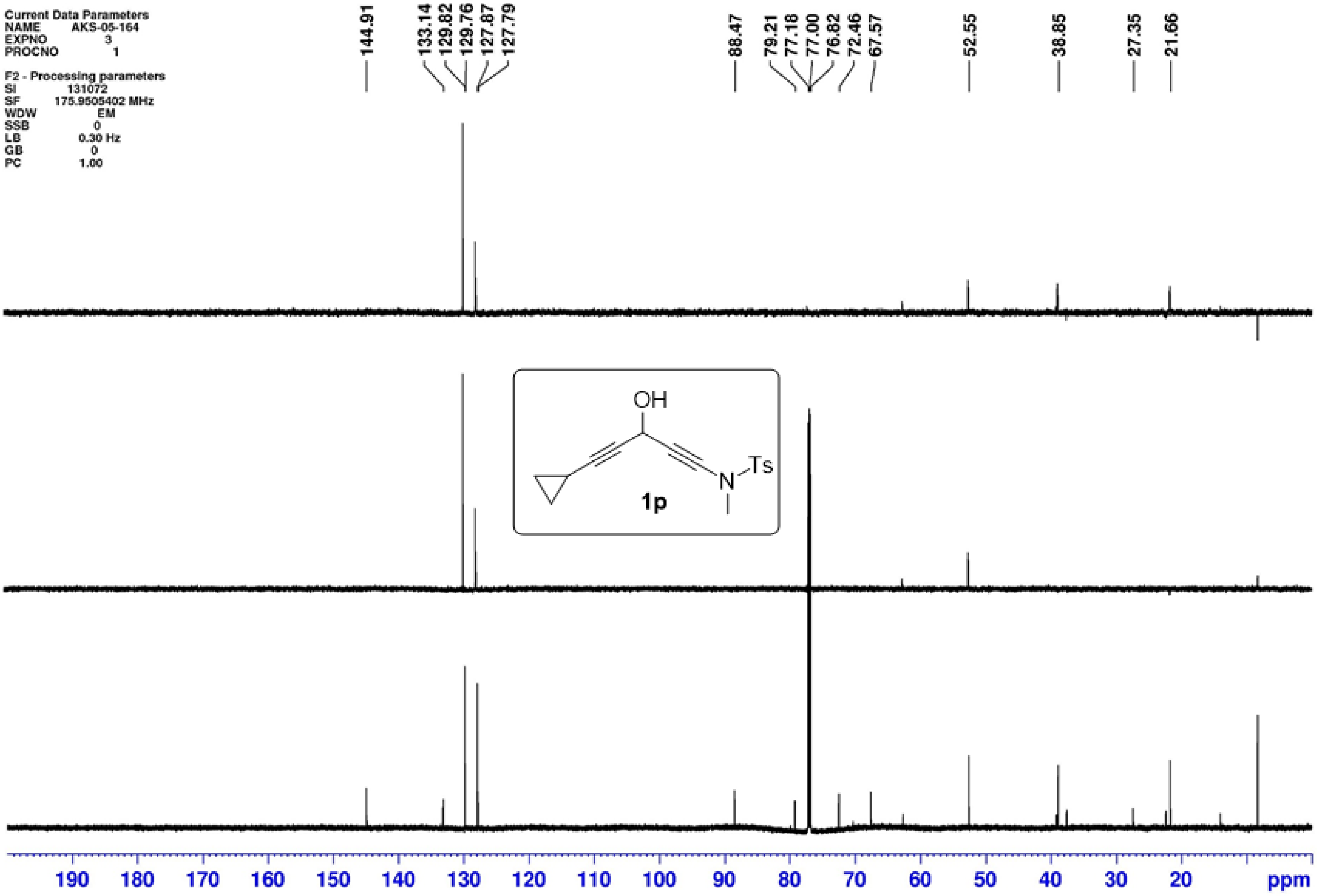

^{1}H NMR ($CDCl_3$, 700 MHz)

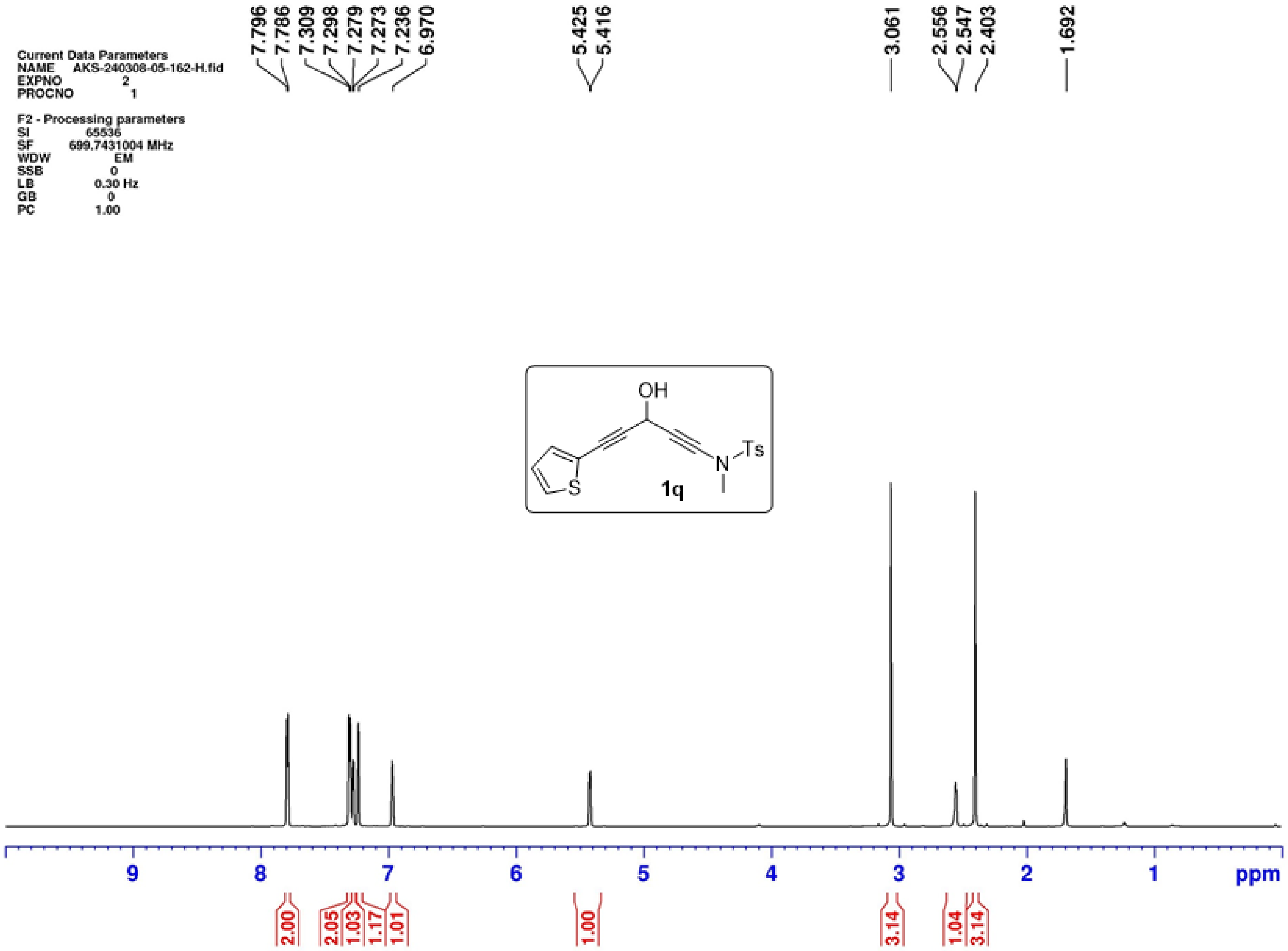

^{13}C{1H} and DEPT NMR ($CDCl_3$, 175 MHz)

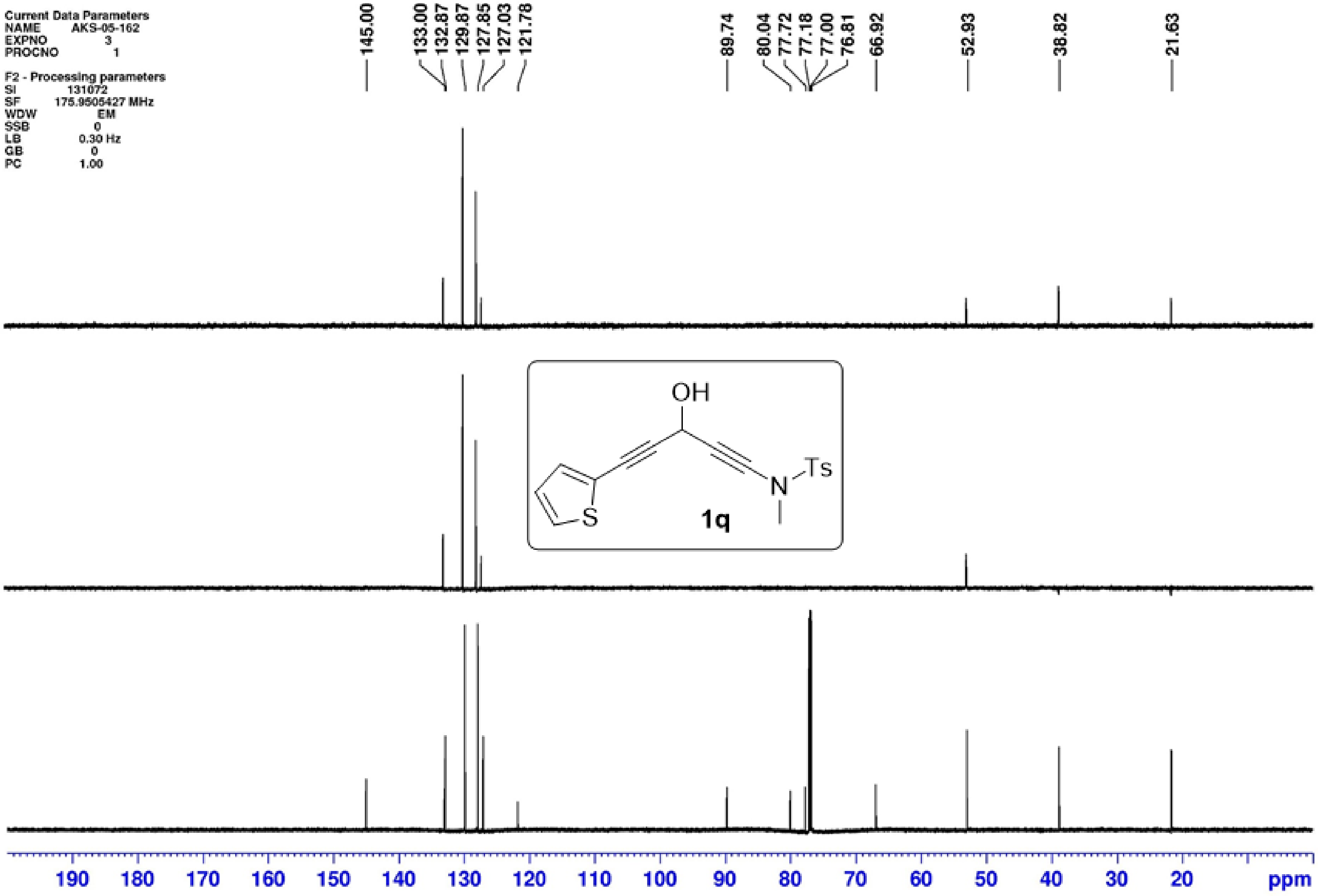

^{1}H NMR ($CDCl_3$, 700 MHz)

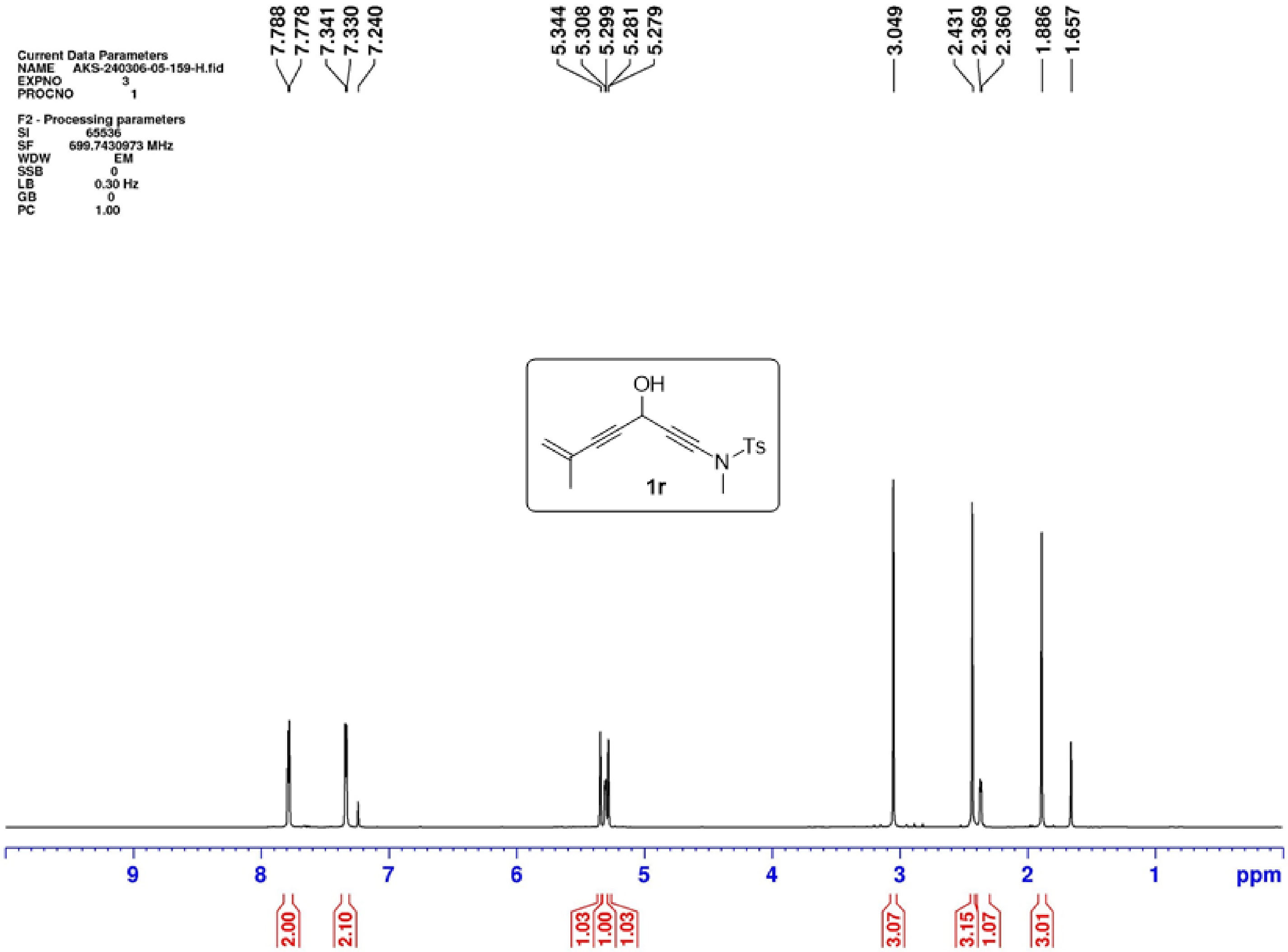

$^{13}C\{1H\}$ and DEPT NMR ($CDCl_3$, 175 MHz)

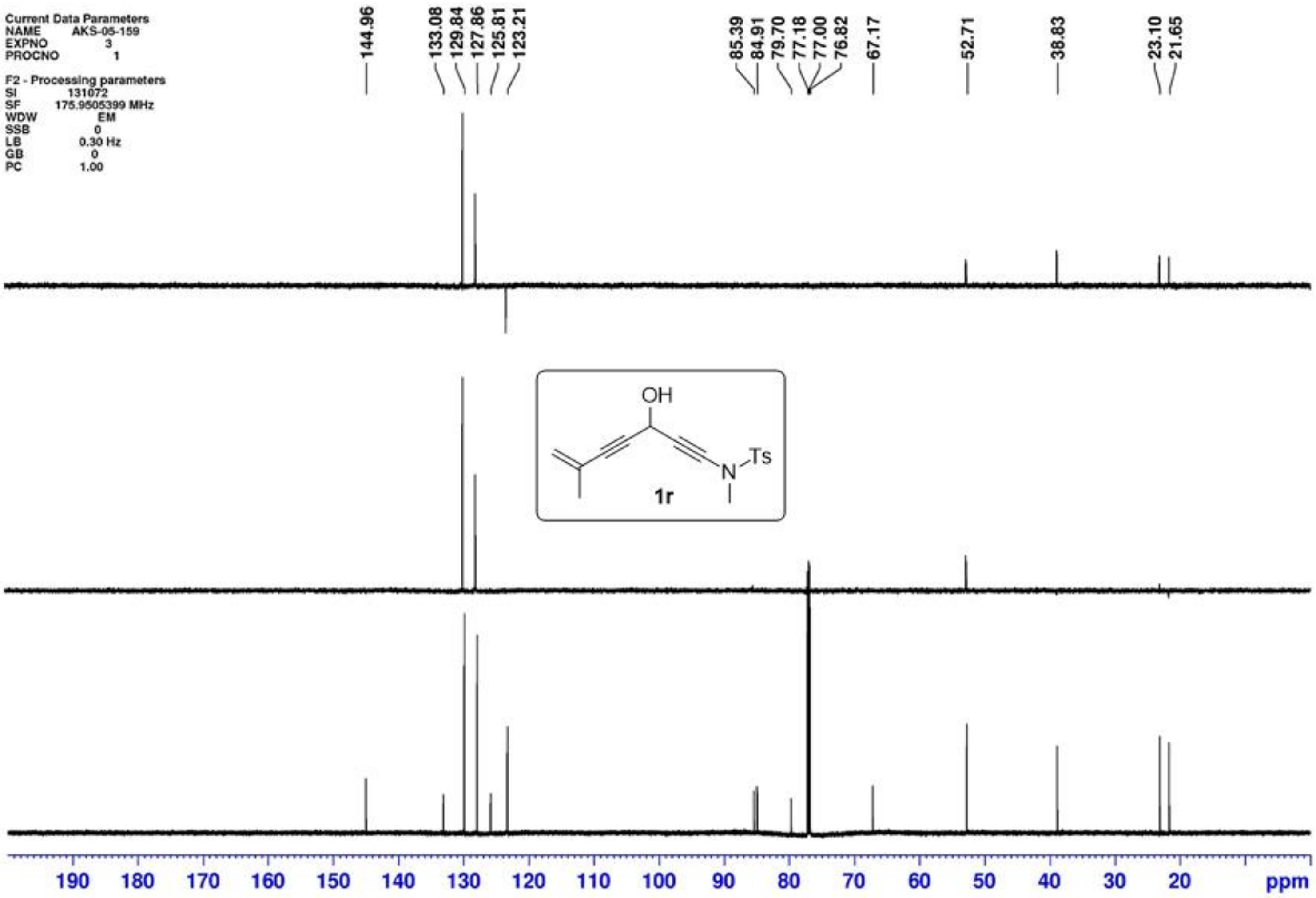

^{1}H NMR ($CDCl_3$, 700 MHz)

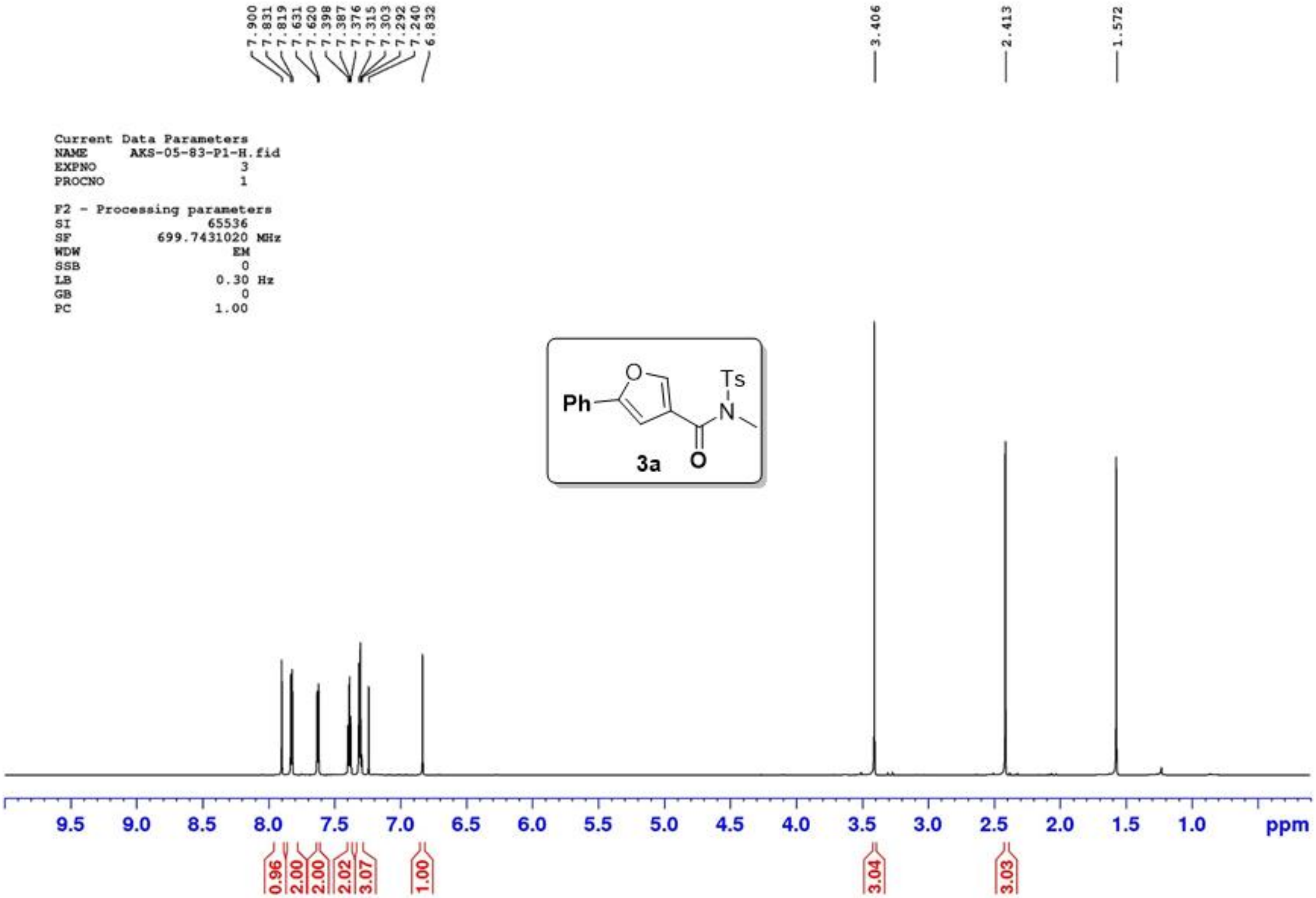

$^{13}C\{1H\}$ and DEPT NMR ($CDCl_3$, 175 MHz)

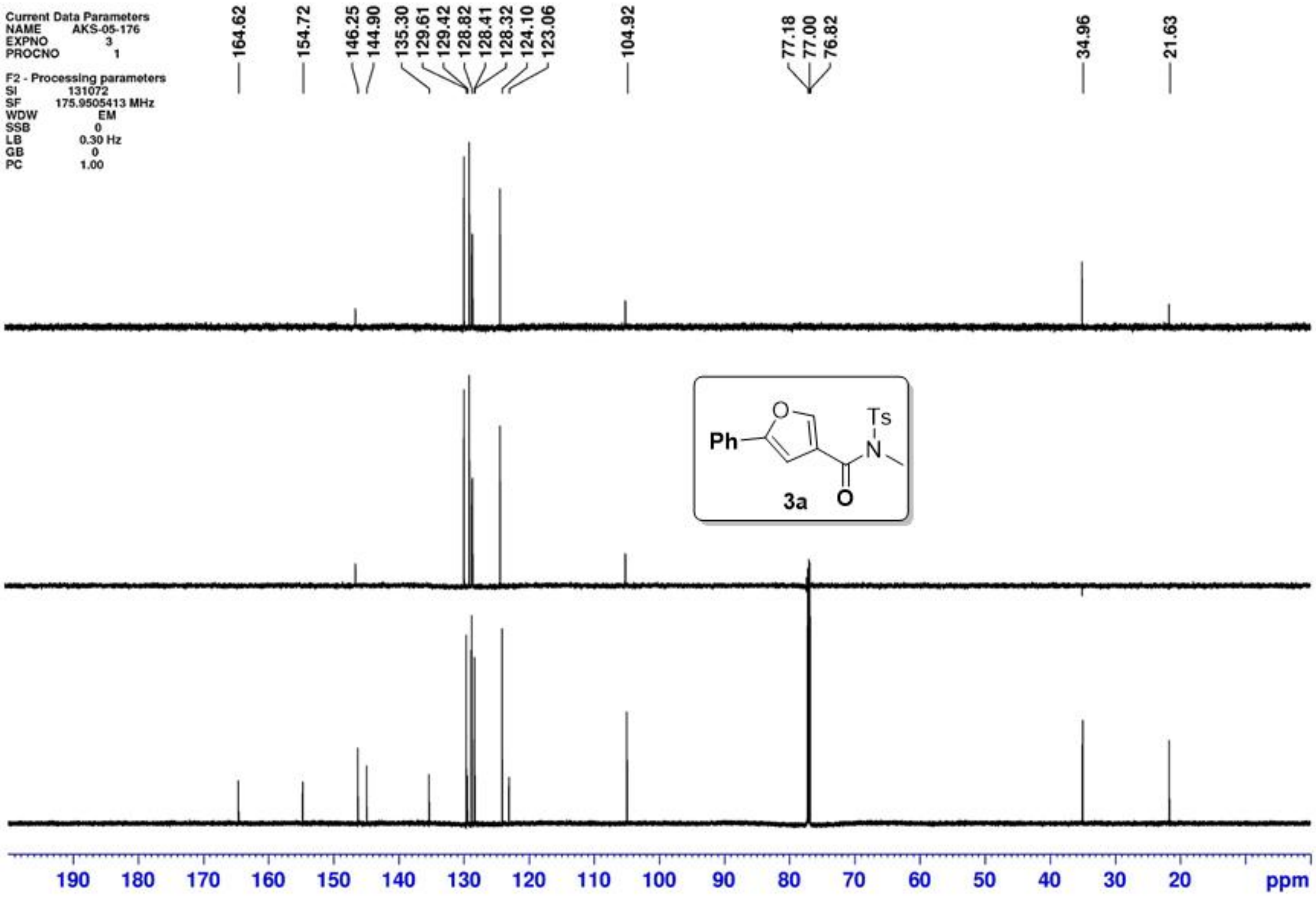

^{1}H NMR ($CDCl_3$, 700 MHz)

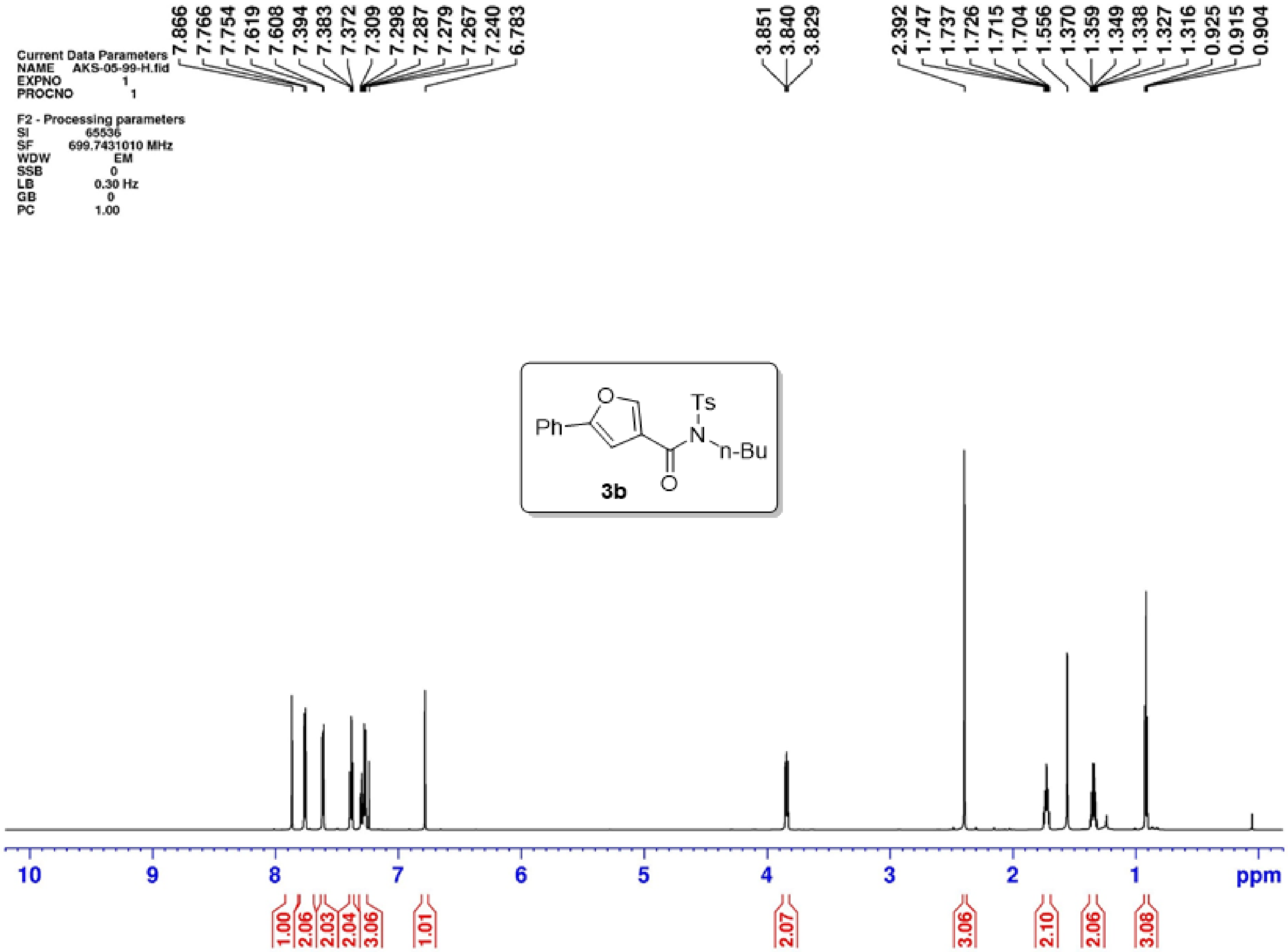

$^{13}C\{1H\}$ and DEPT NMR ($CDCl_3$, 175 MHz)

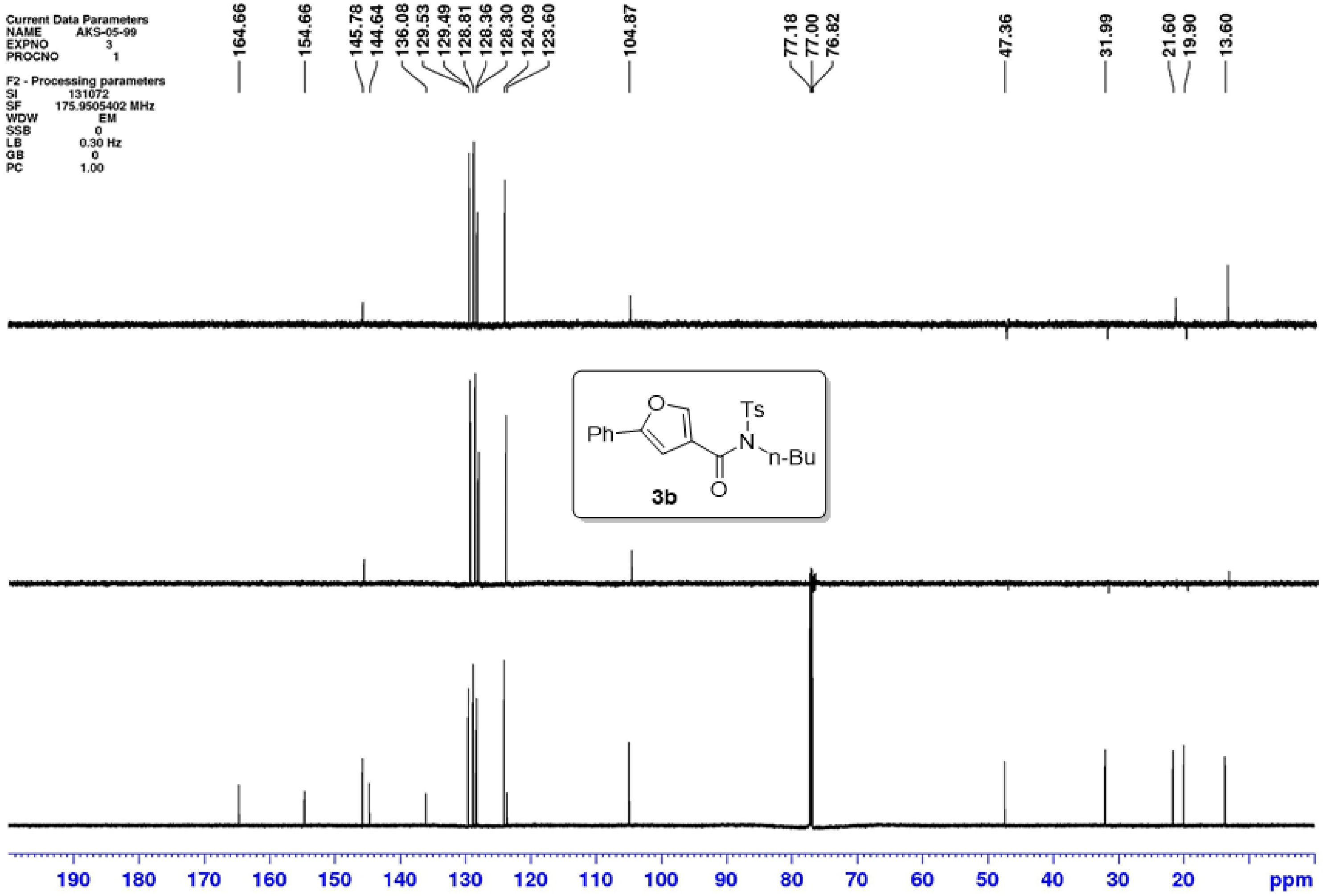

^{1}H NMR ($CDCl_3$, 700 MHz)

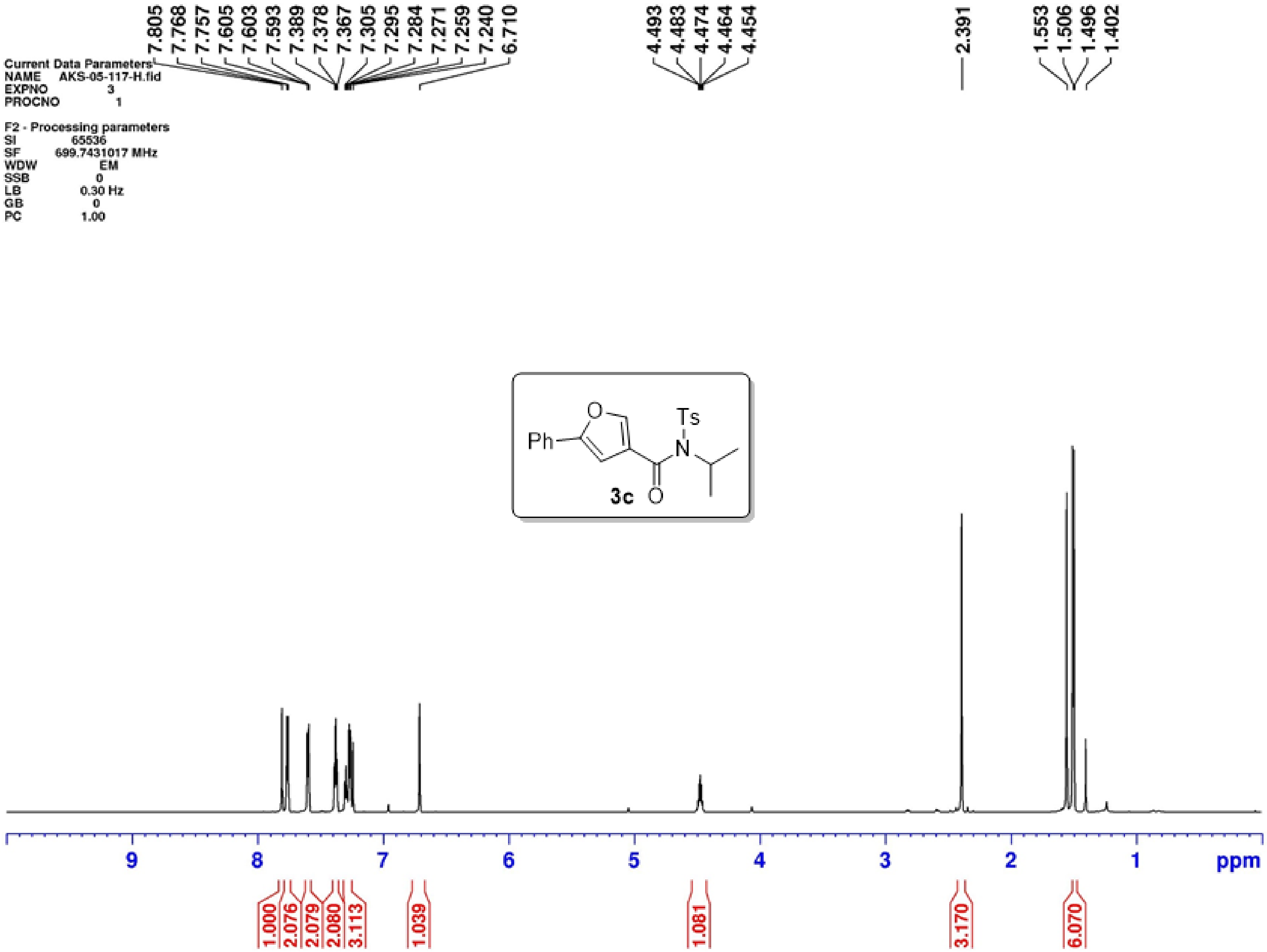

$^{13}C\{1H\}$ and DEPT NMR ($CDCl_3$, 175 MHz)

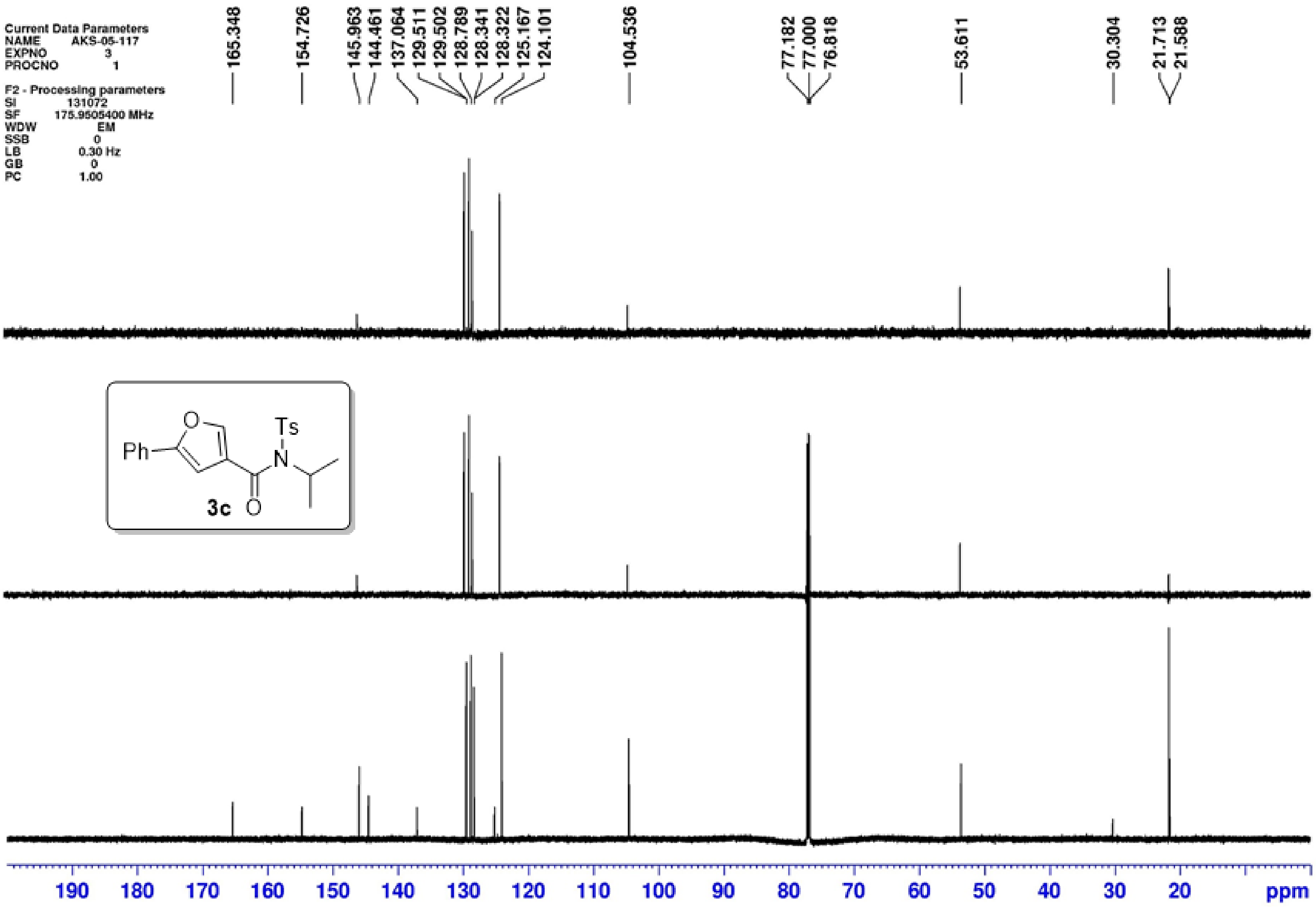

^{1}H NMR ($CDCl_3$, 700 MHz)

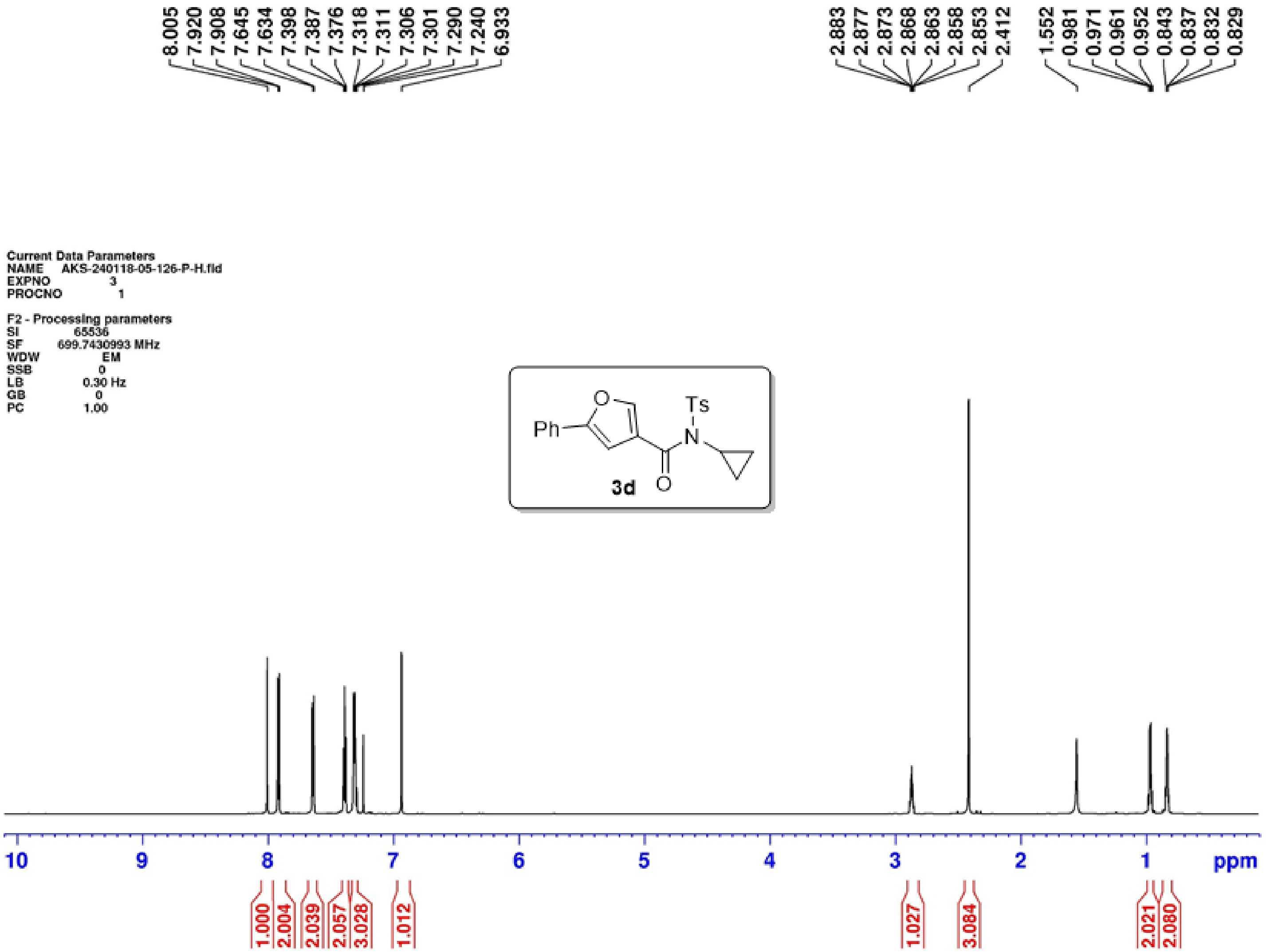

$^{13}C\{1H\}$ and DEPT NMR ($CDCl_3$, 175 MHz)

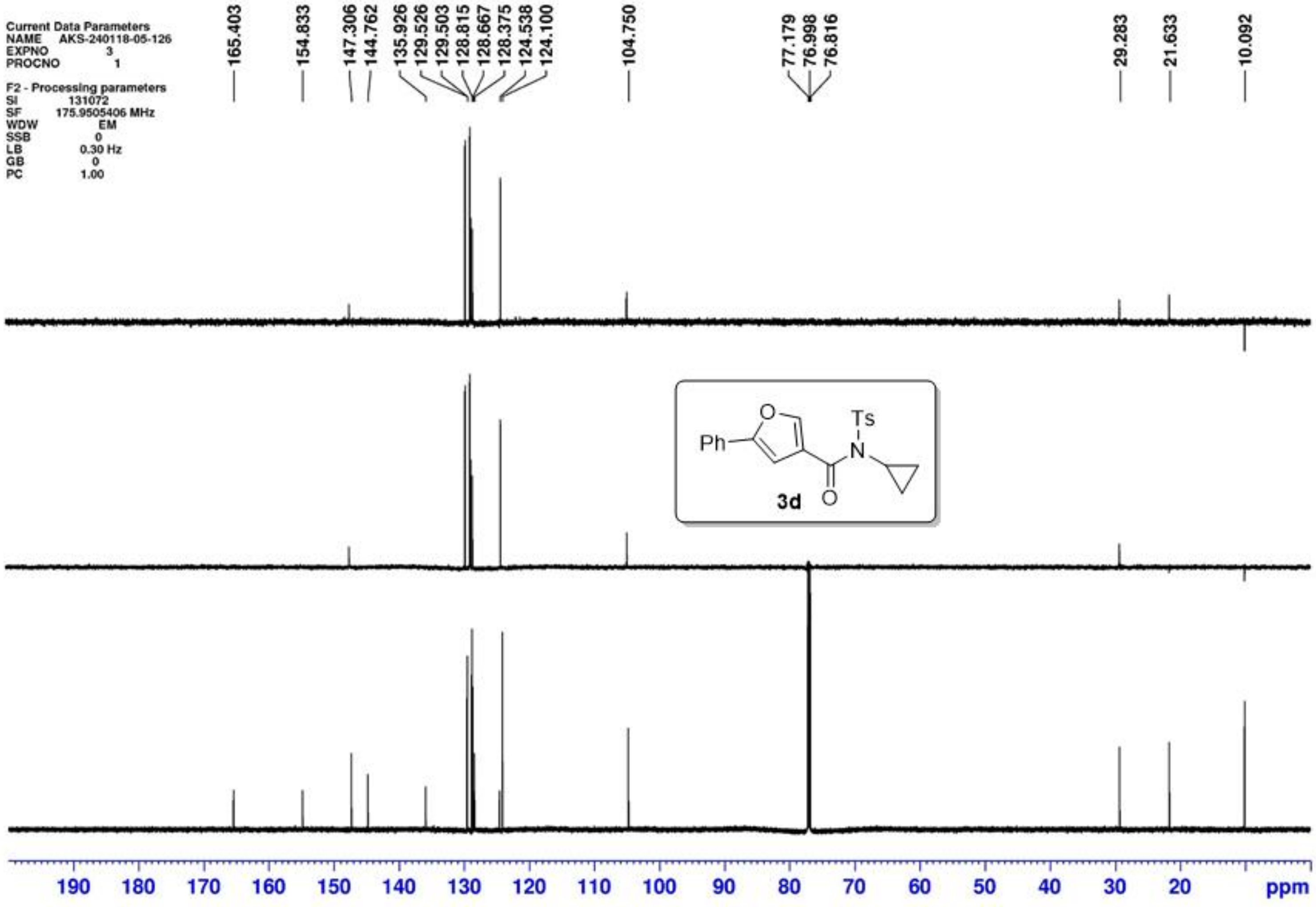

^1H NMR ($CDCl_3$, 700 MHz)

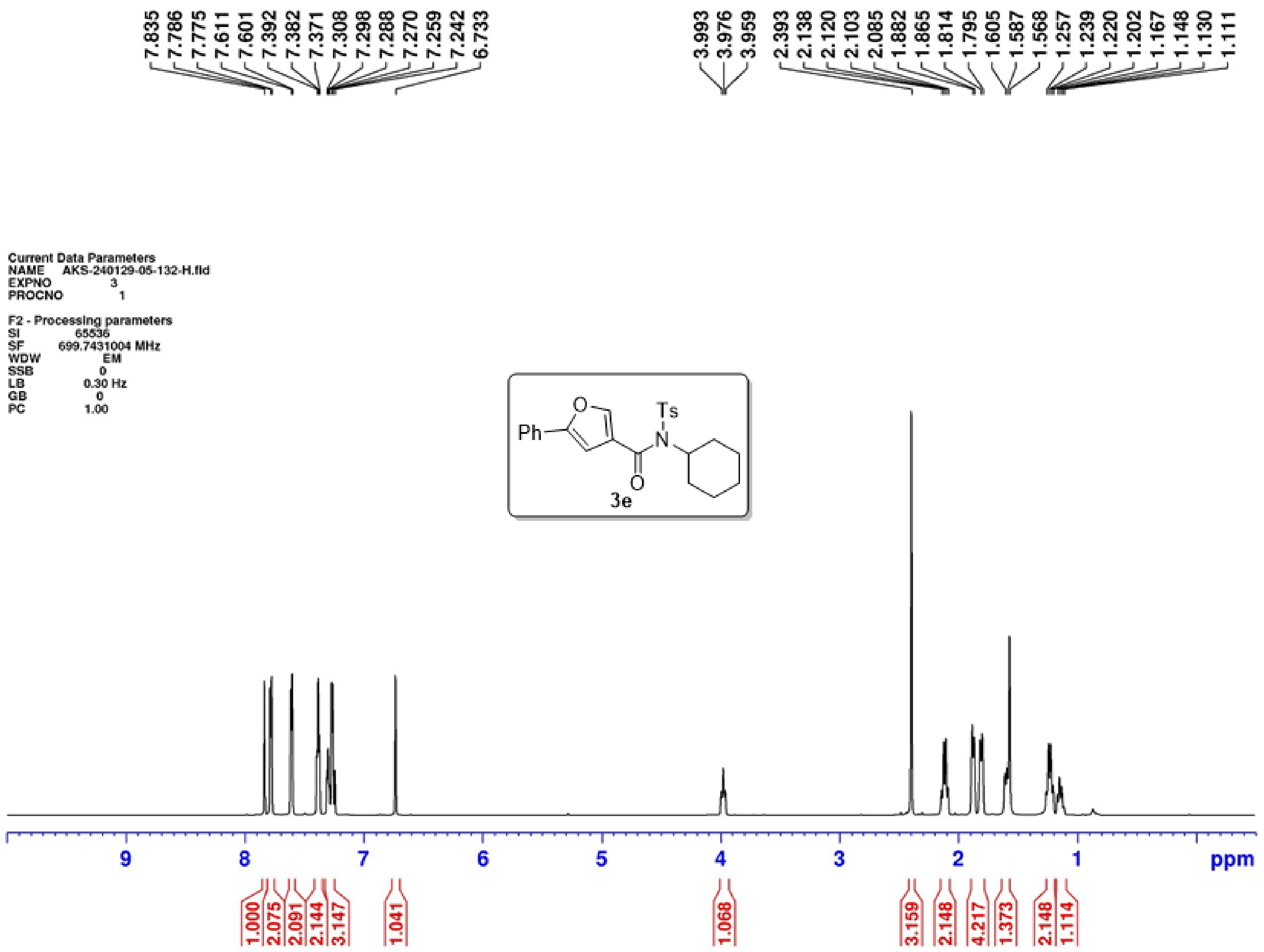

$^{13}C\{1H\}$ and DEPT NMR ($CDCl_3$, 175 MHz)

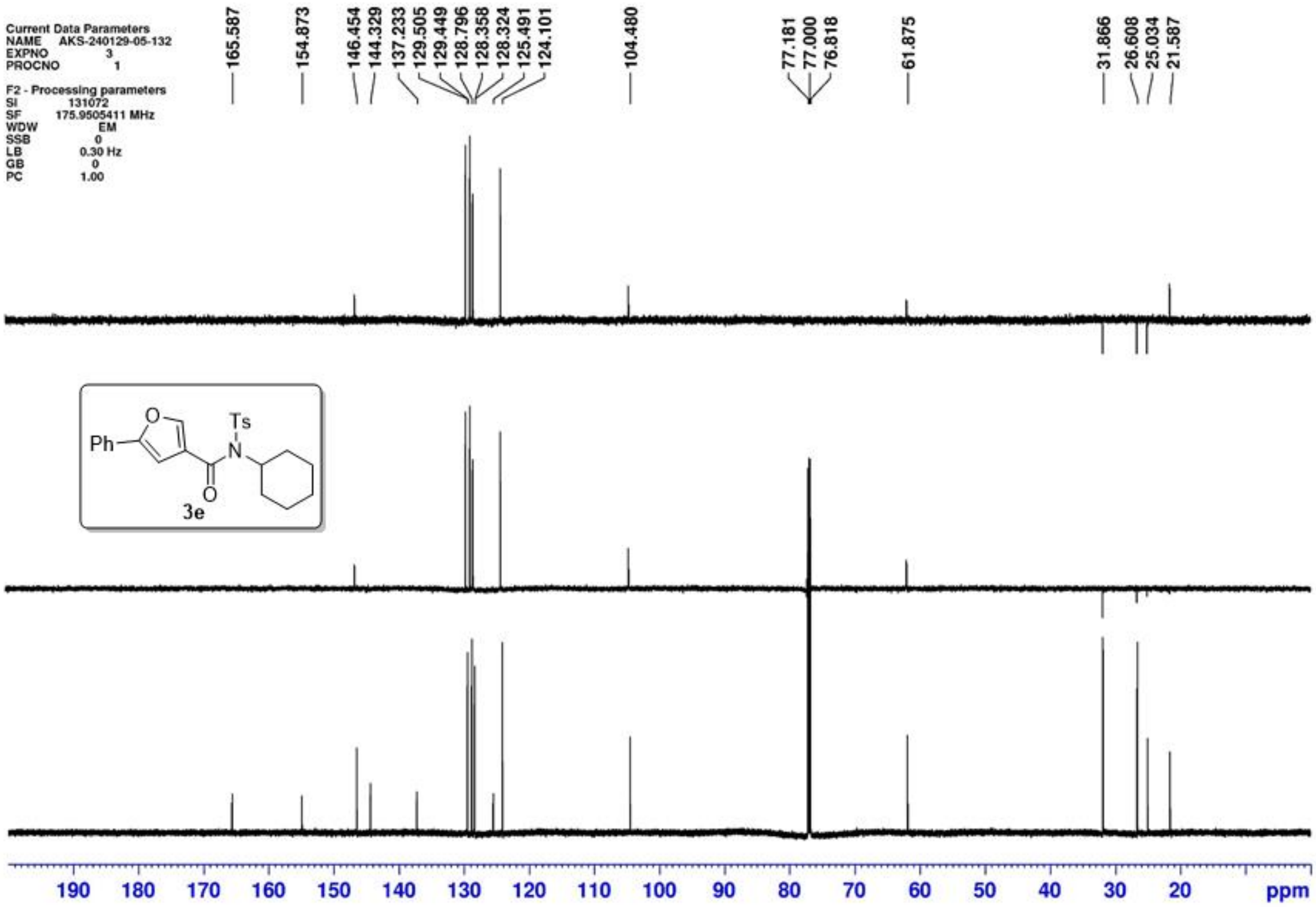

^{1}H NMR ($CDCl_3$, 700 MHz)

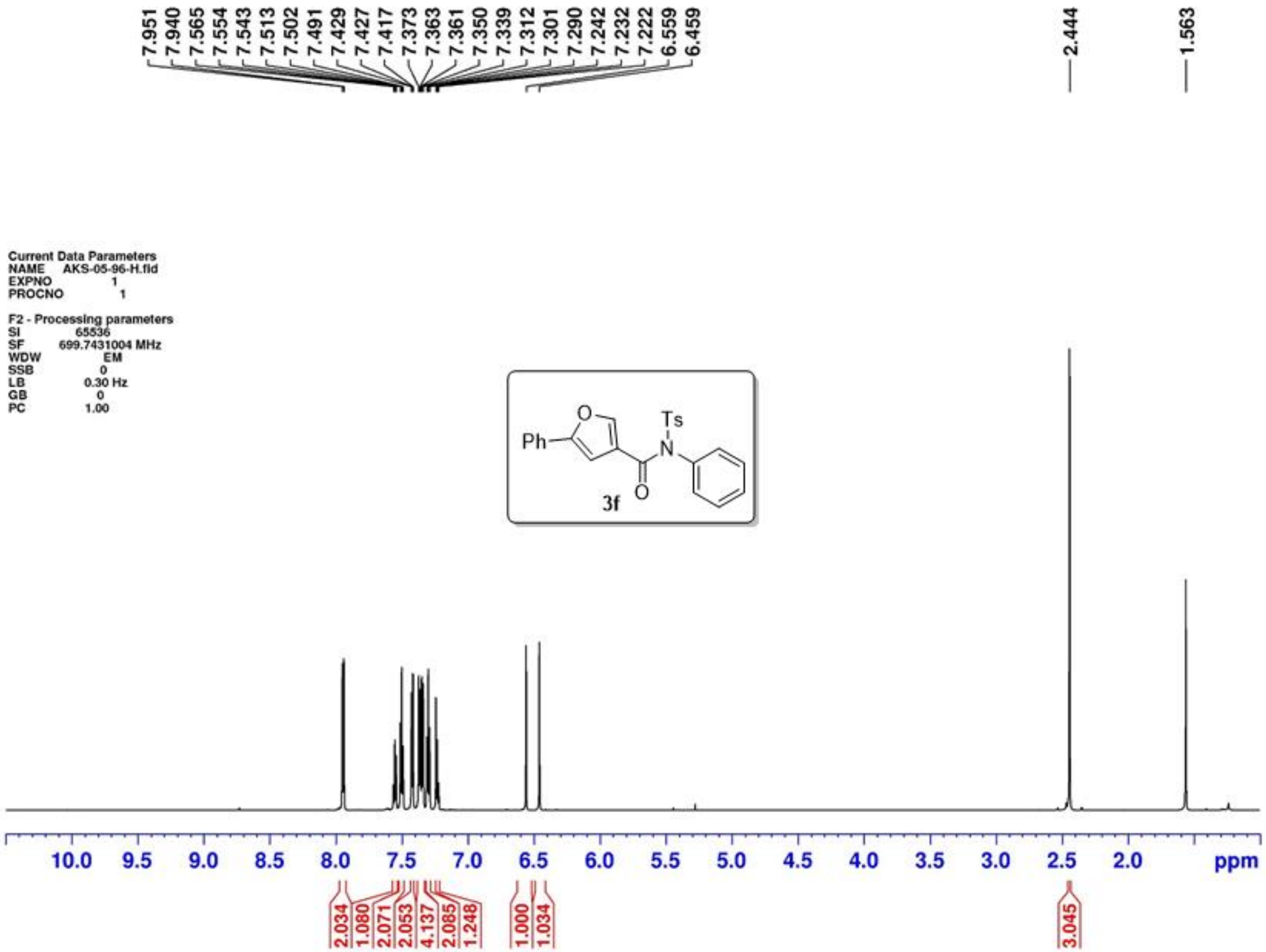

$^{13}C\{1H\}$ and DEPT NMR ($CDCl_3$, 175 MHz)

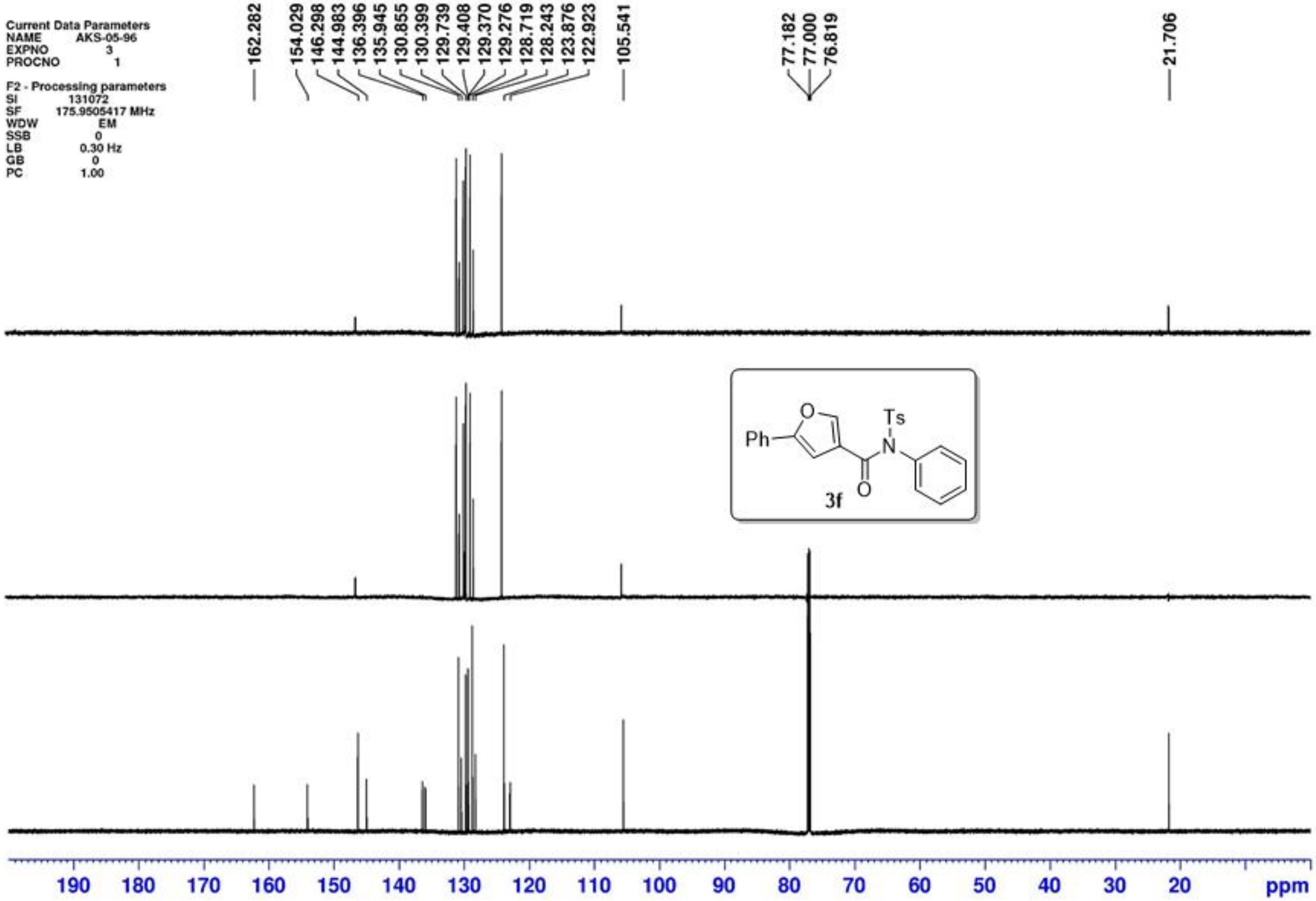

^{1}H NMR ($CDCl_3$, 700 MHz)

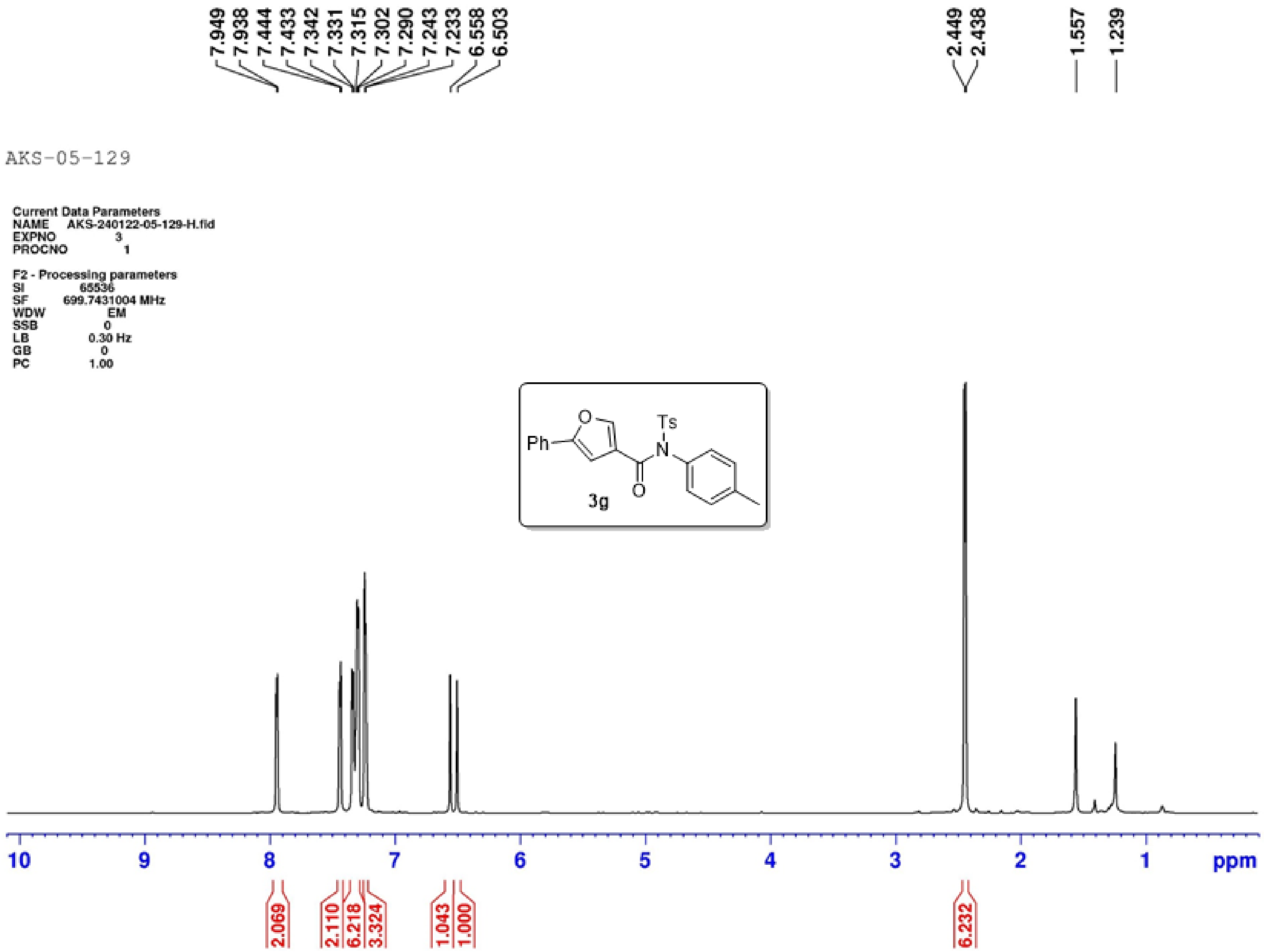

$^{13}C\{1H\}$ and DEPT NMR ($CDCl_3$, 175 MHz)

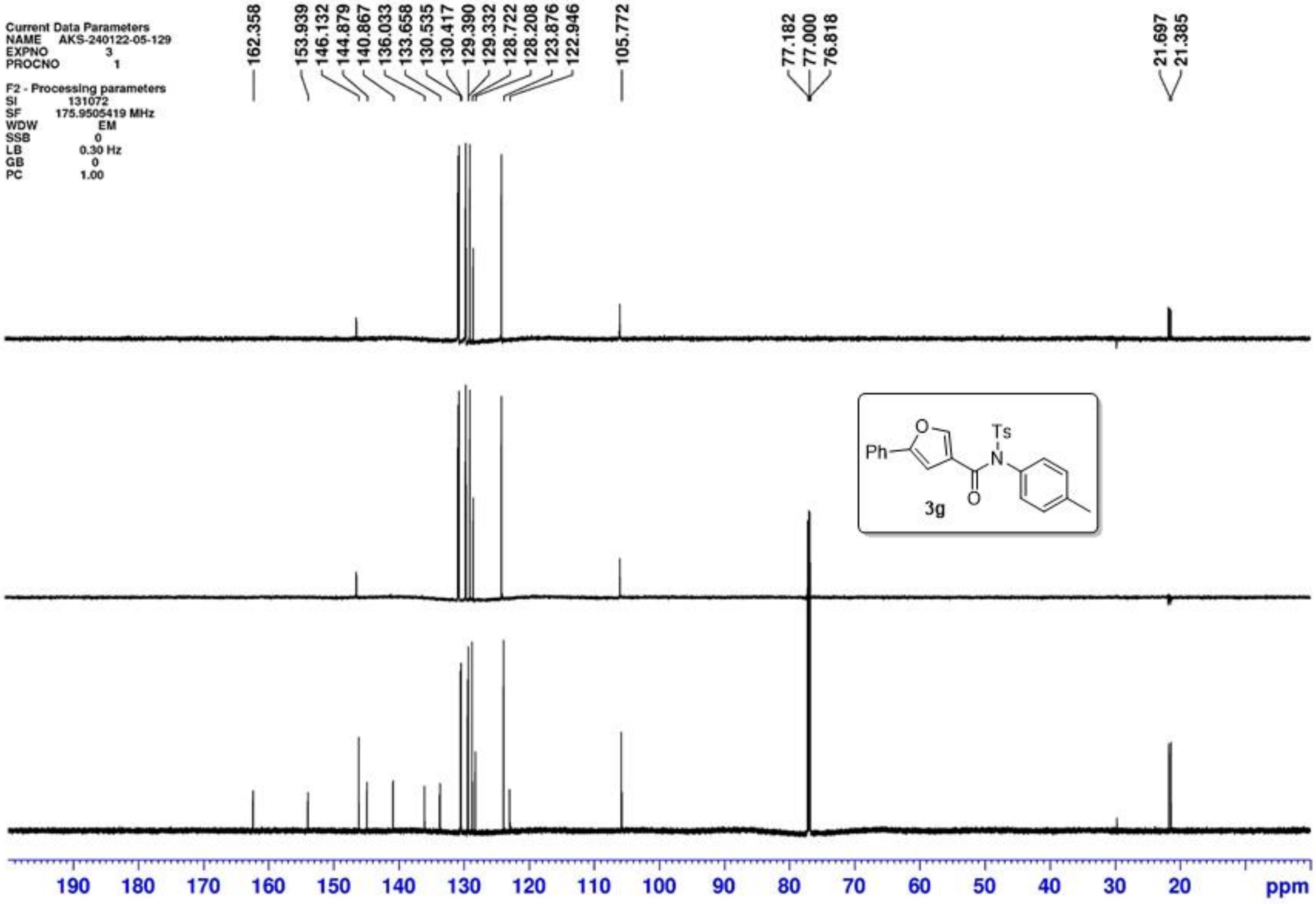

^{1}H NMR ($CDCl_3$, 700 MHz)

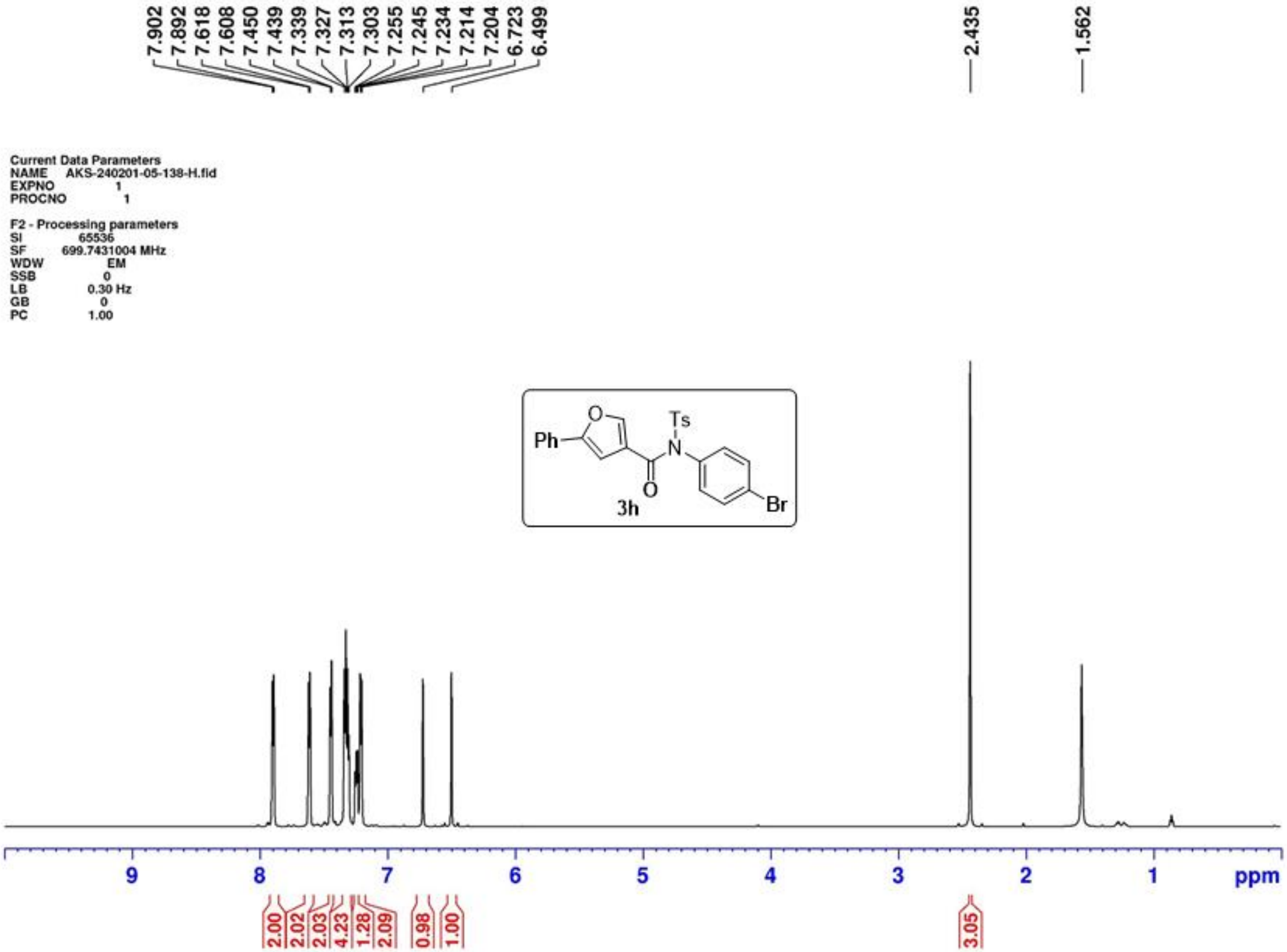

$^{13}C\{1H\}$ and DEPT NMR ($CDCl_3$, 175 MHz)

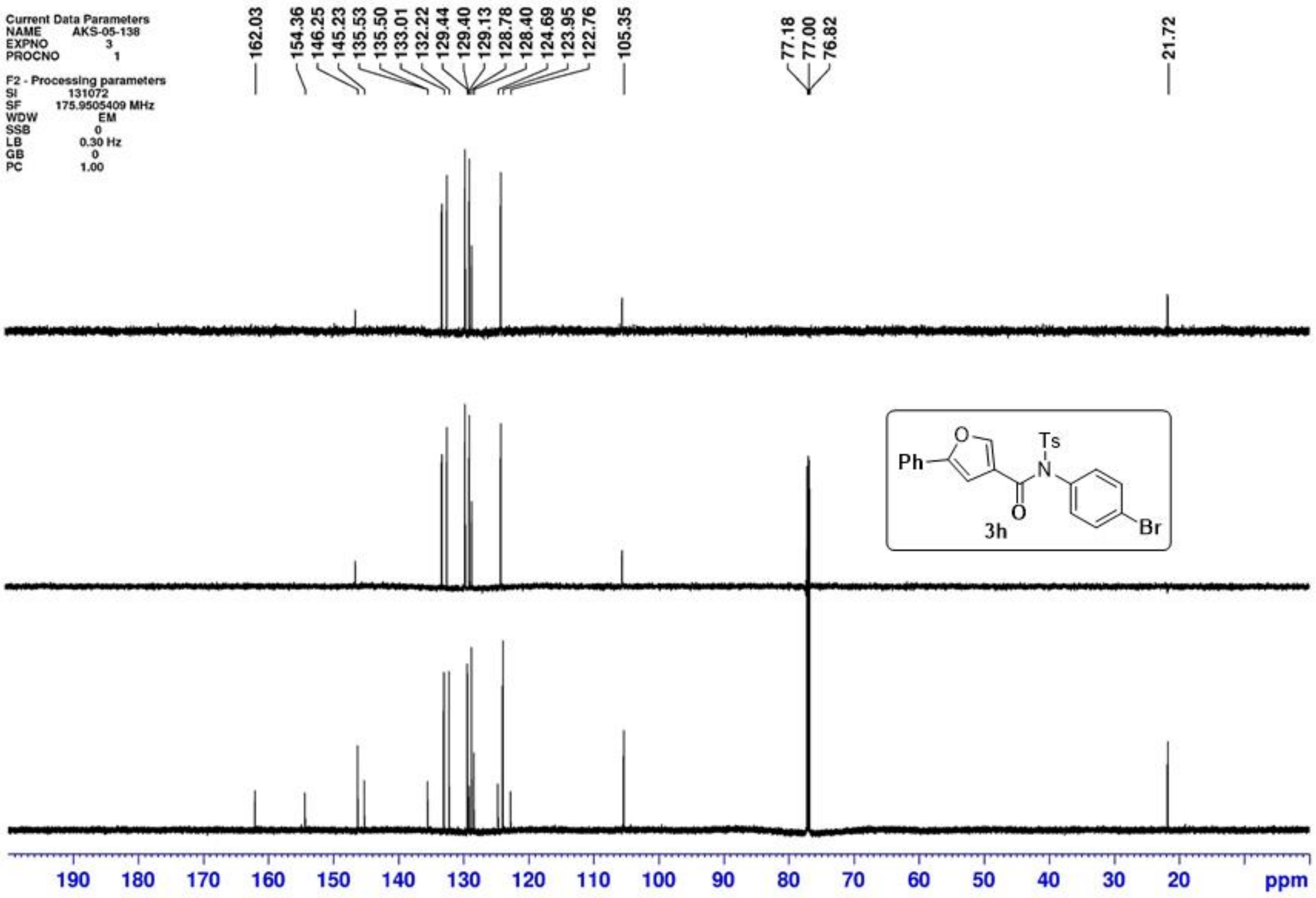

^{1}H NMR ($CDCl_3$, 700 MHz)

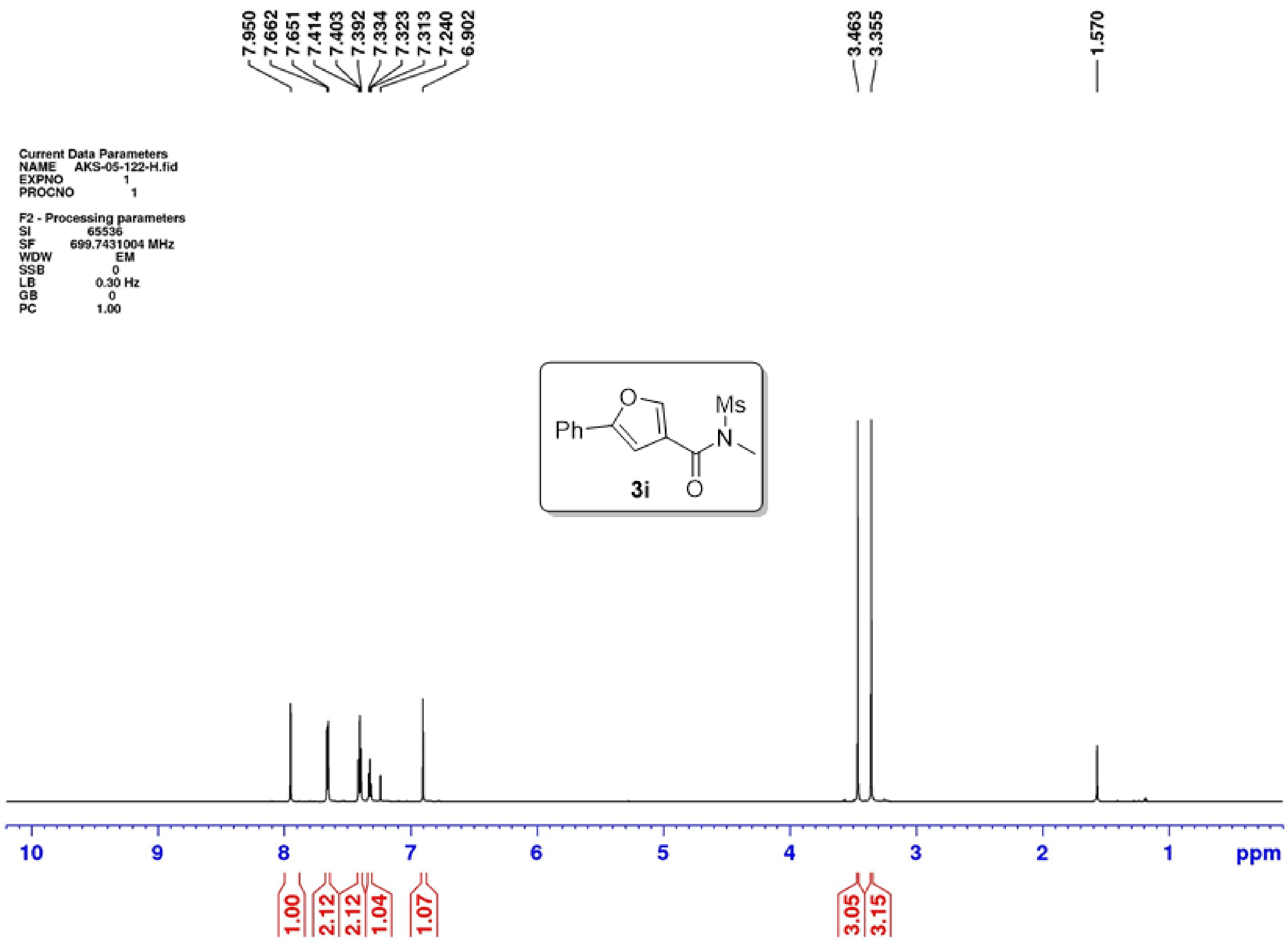

$^{13}C\{1H\}$ **and DEPT NMR ($CDCl_3$, 175 MHz)**

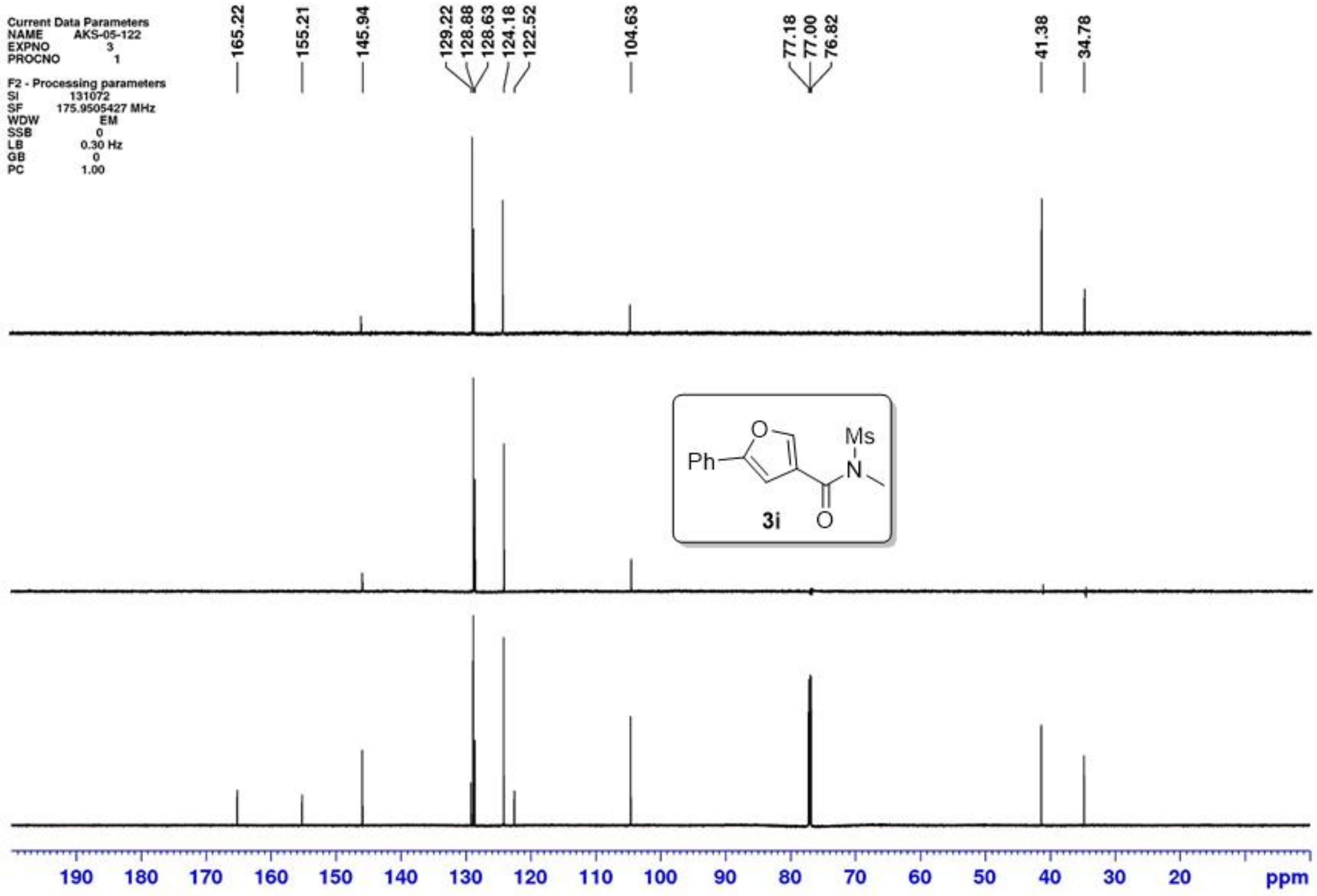

^{1}H NMR ($CDCl_3$, 700 MHz)

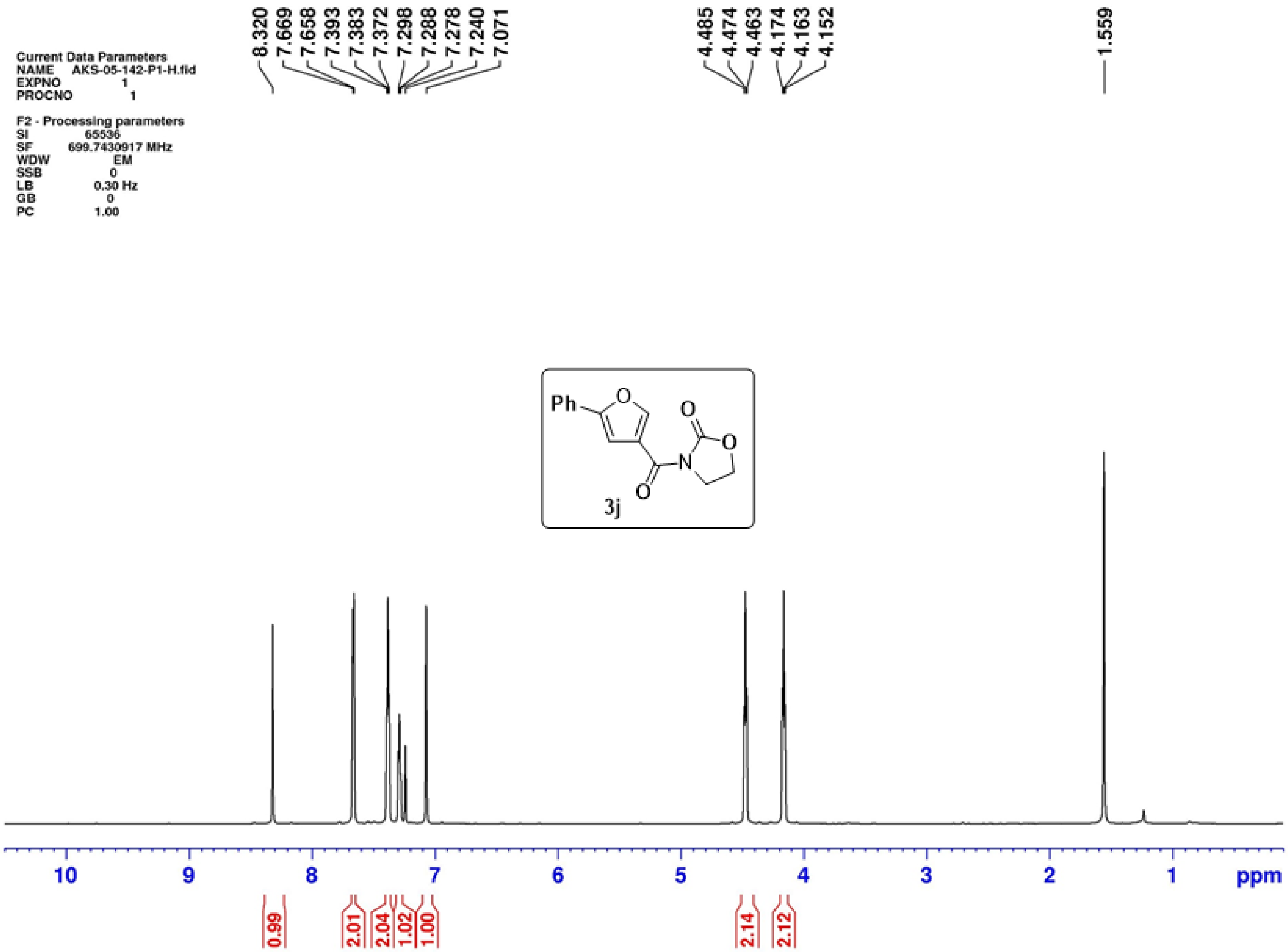

^{13}C{1H} and DEPT NMR (CDCl$_3$, 175 MHz)

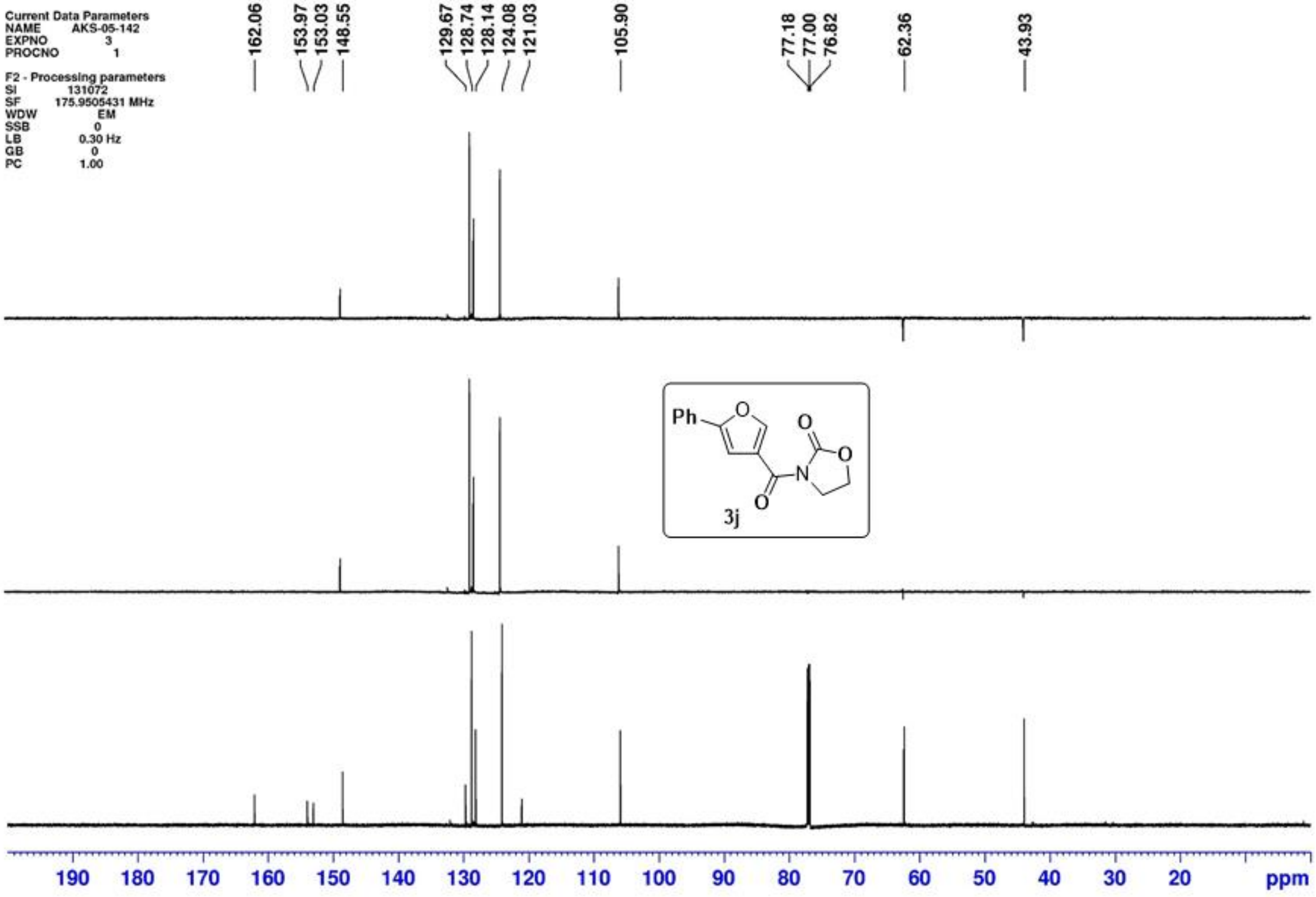

^{1}H NMR ($CDCl_3$, 700 MHz)

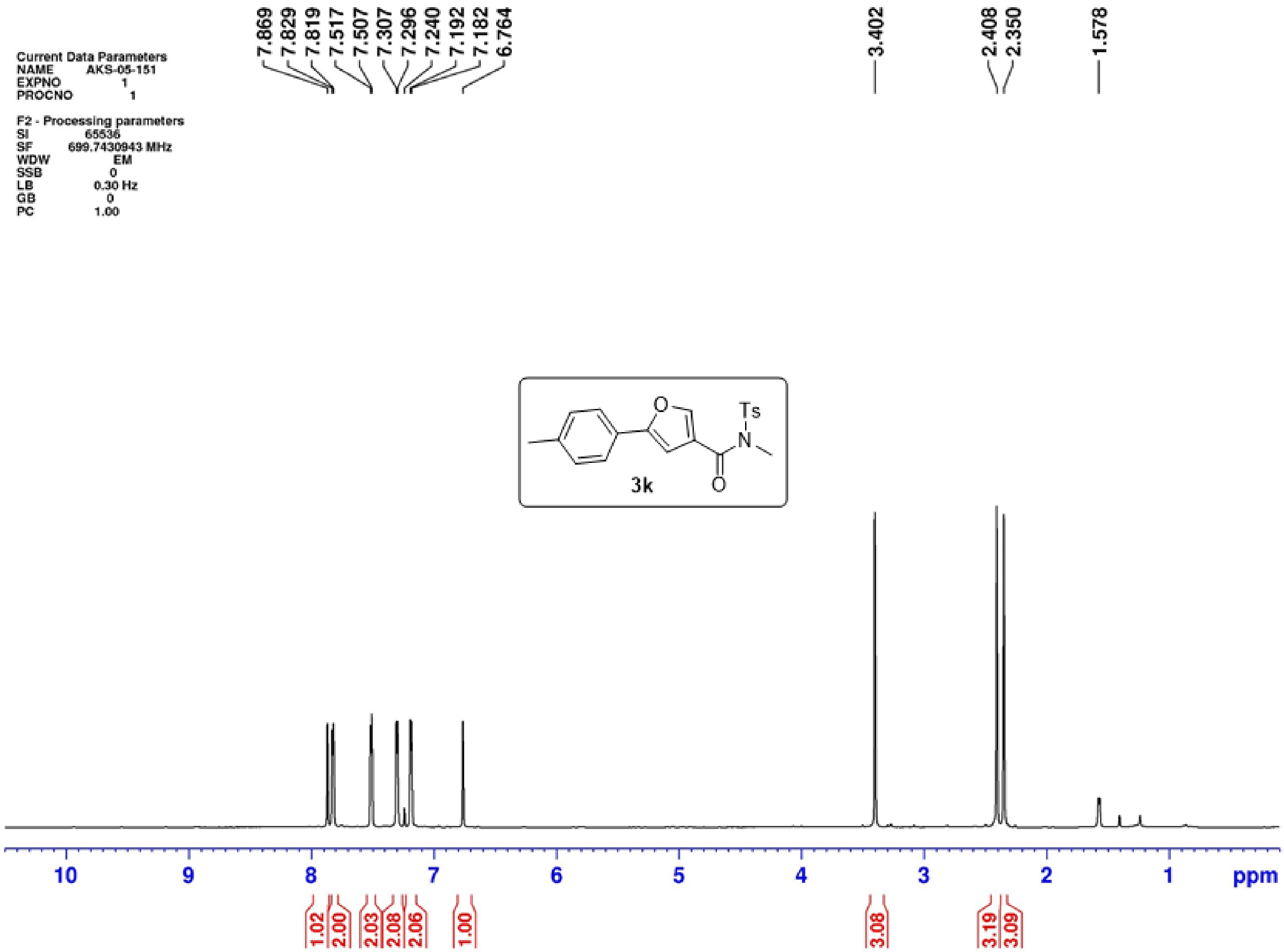

$^{13}C\{1H\}$ and DEPT NMR ($CDCl_3$, 175 MHz)

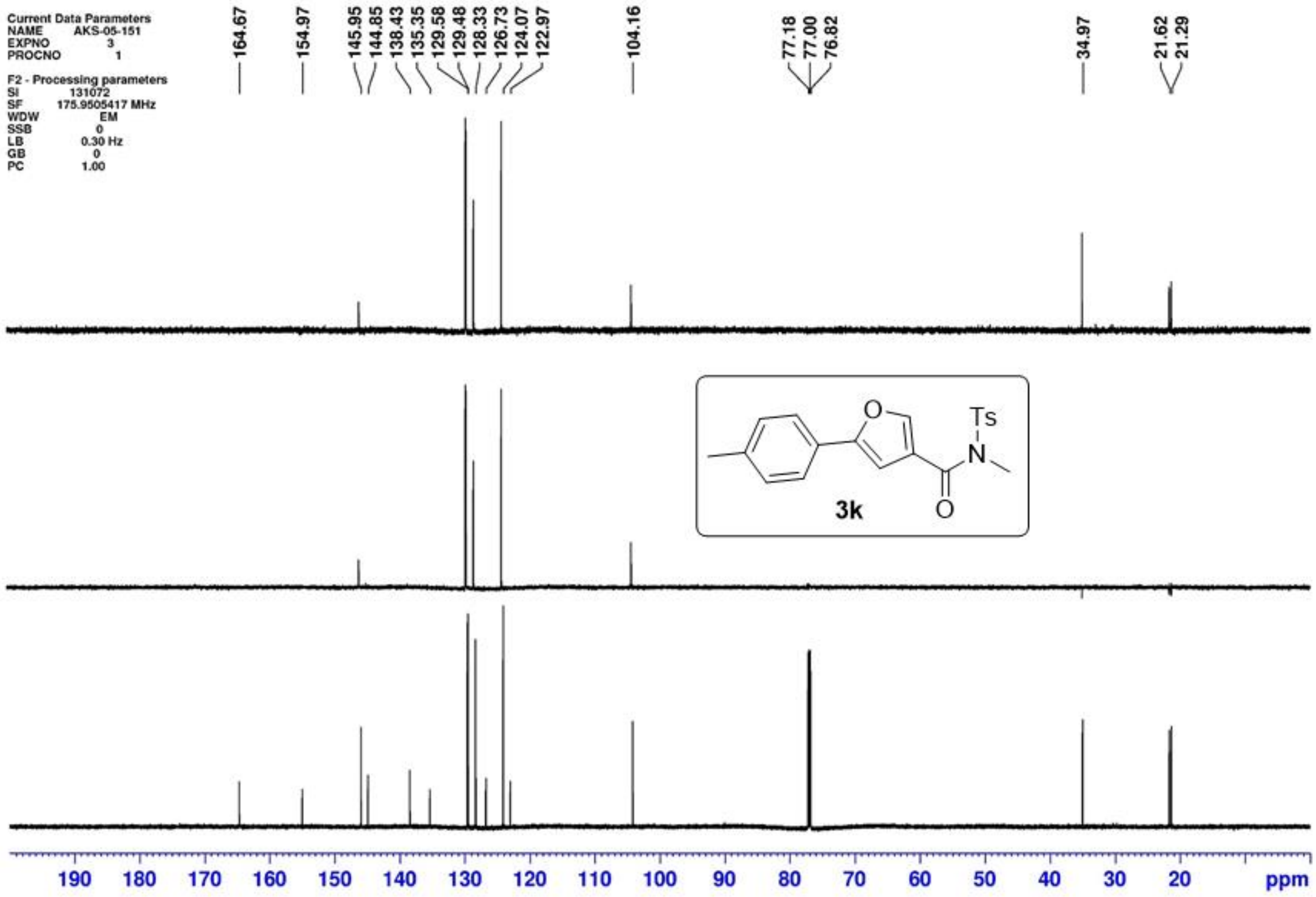

^{1}H NMR ($CDCl_3$, 700 MHz)

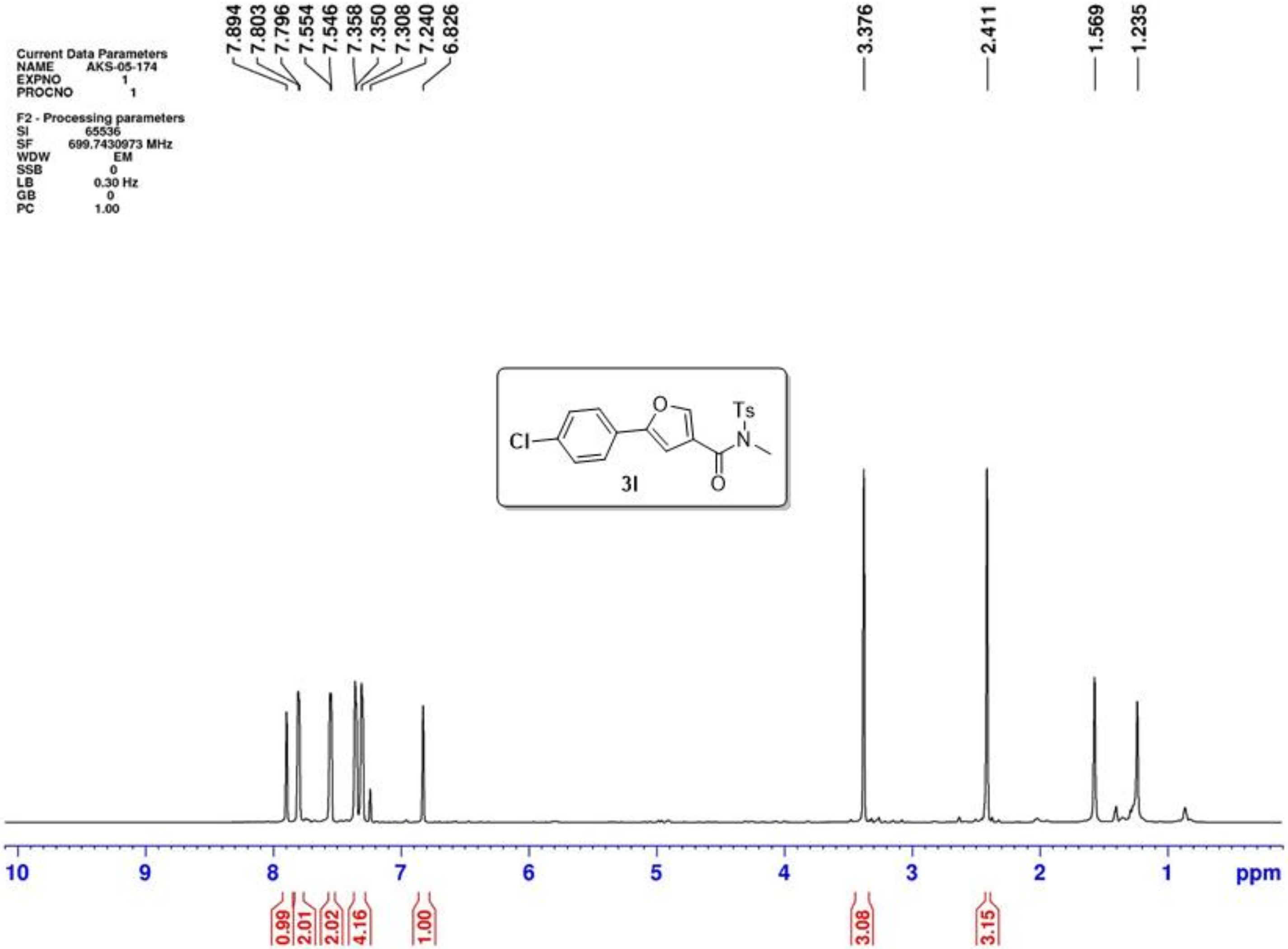

^{13}C{1H} and DEPT NMR ($CDCl_3$, 175 MHz)

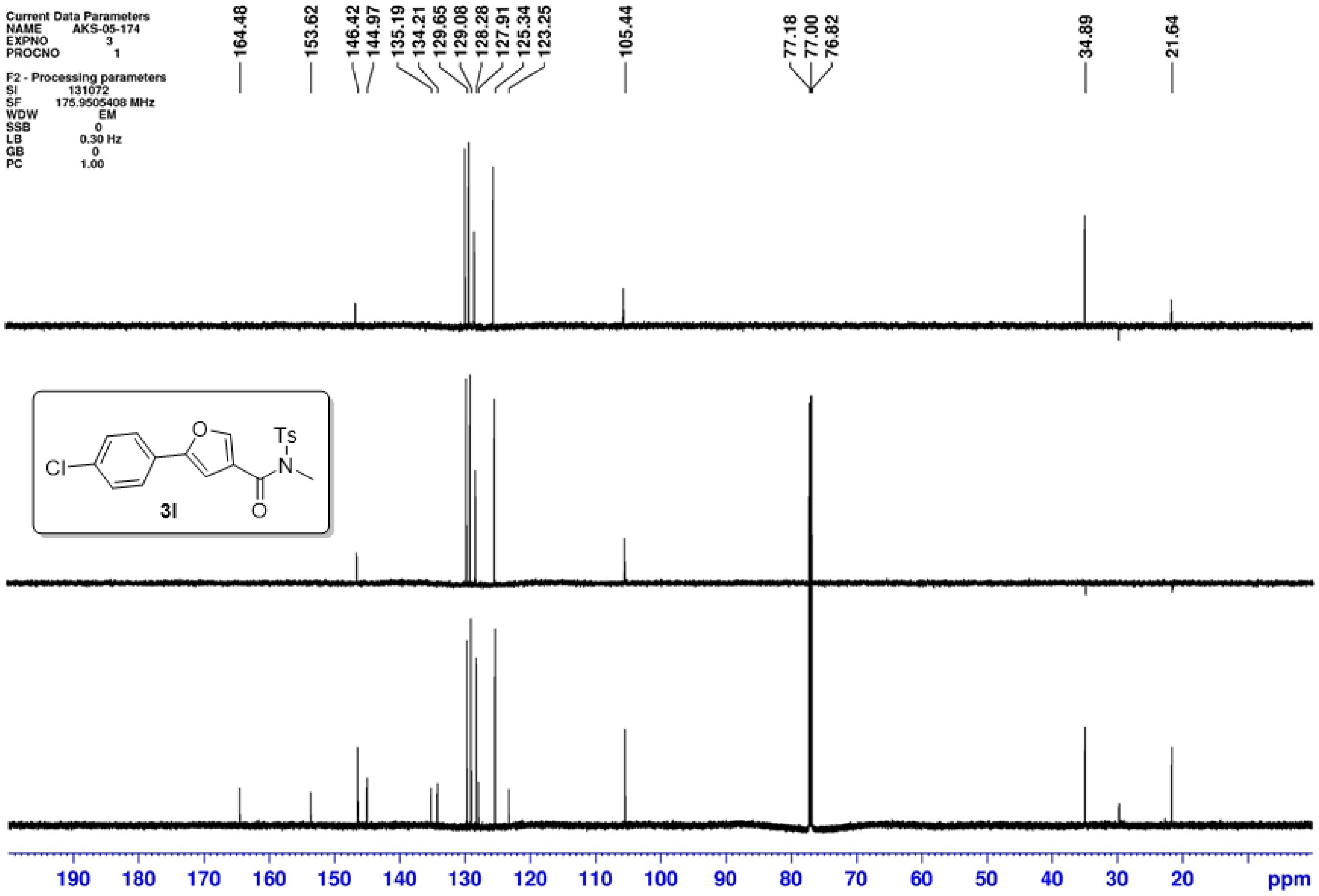

^{1}H NMR ($CDCl_3$, 400 MHz)

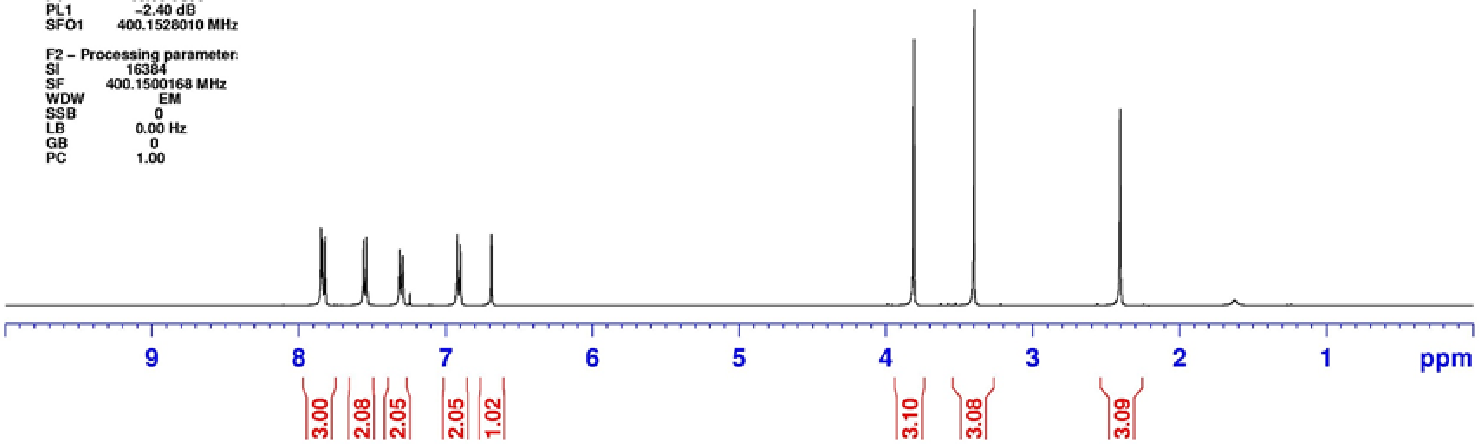

DB-A-199

Current Data Parameters
NAME DB-A-199
EXPNO 2
PROCNO 1

F2 - Acquisition Parameter
Date_ 20240805
Time 23.02
INSTRUM spect
PROBHD 5 mm DUL 13C-
PULPROG zg30
TD 32768
SOLVENT CDCl3
NS 52
DS 0
SWH 6410.256 Hz
FIDRES 0.195625 Hz
AQ 2.5559540 sec
RG 181
DW 78.000 usec
DE 6.00 usec
TE 300.0 K
D1 2.00000000 sec
TD0 1

======== CHANNEL f1 ===
NUC1 1H
P1 10.00 usec
PL1 -2.40 dB
SFO1 400.1528010 MHz

F2 - Processing parameter
SI 16384
SF 400.1500168 MHz
WDW EM
SSB 0
LB 0.00 Hz
GB 0
PC 1.00

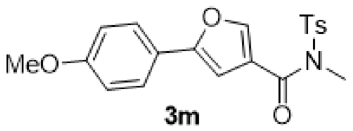

3m

$^{13}C\{1H\}$ NMR ($CDCl_3$, 100 MHz)

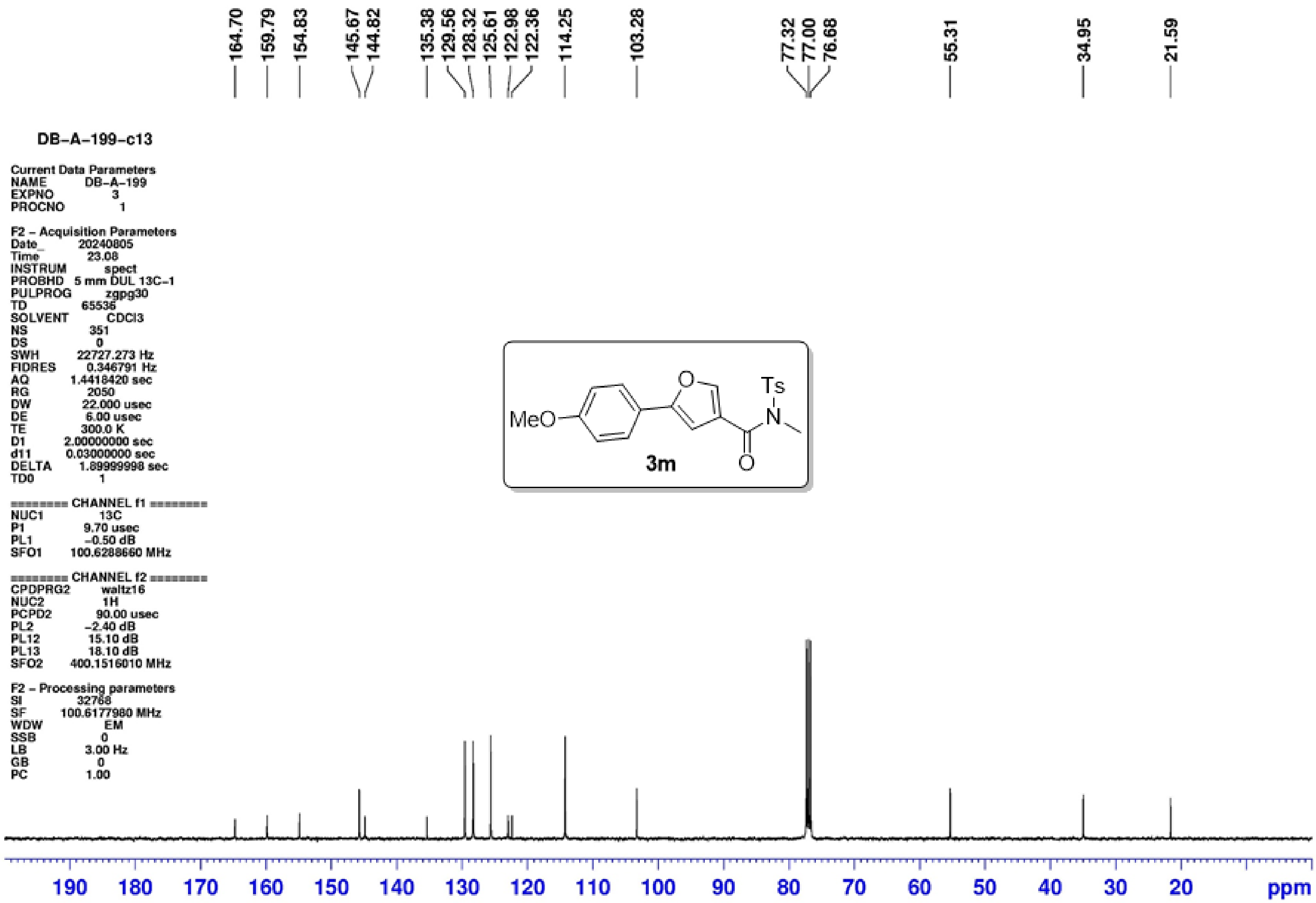

^{1}H NMR ($CDCl_3$, 700 MHz)

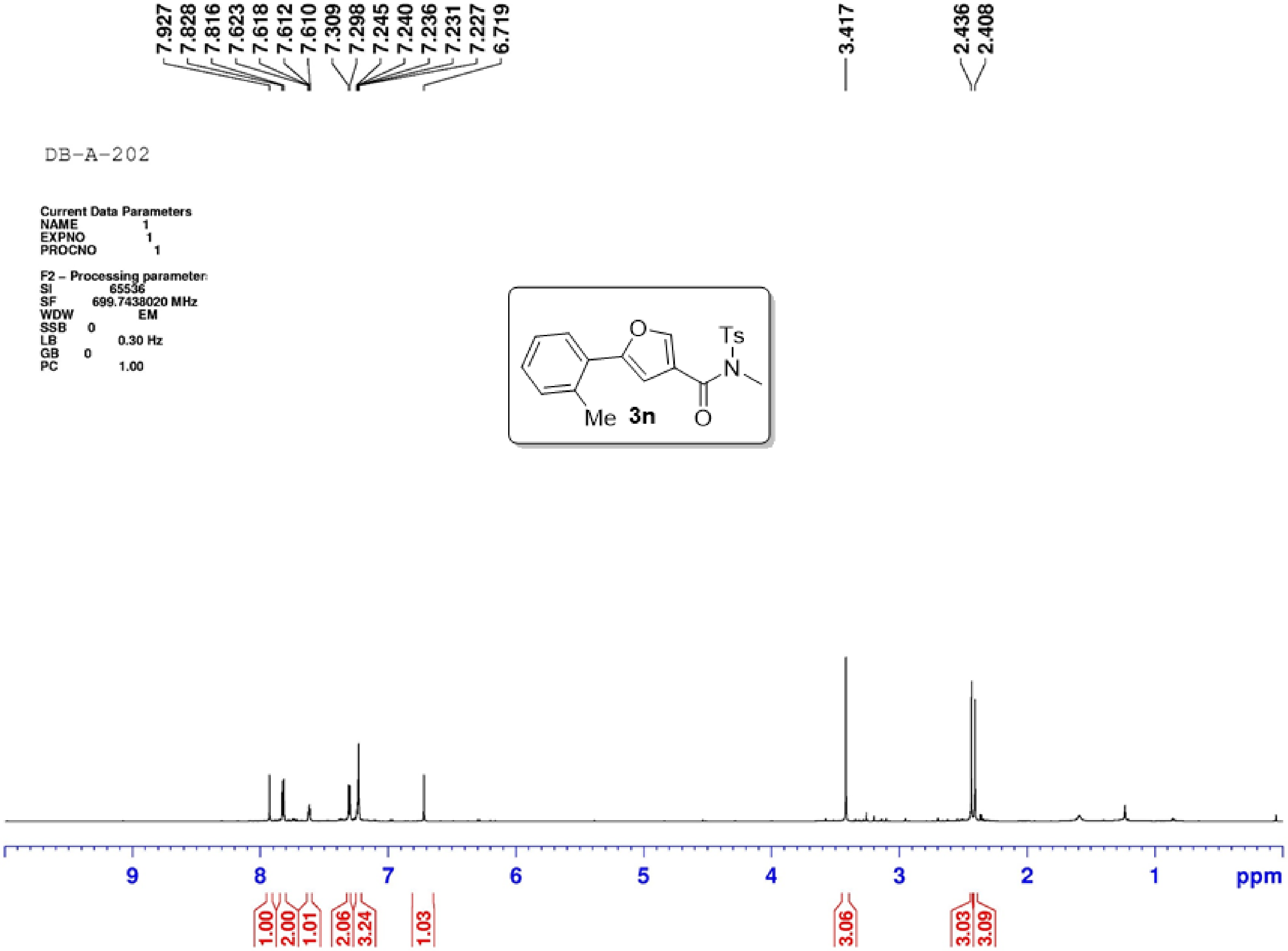

$^{13}C\{1H\}$ NMR ($CDCl_3$, 175 MHz)

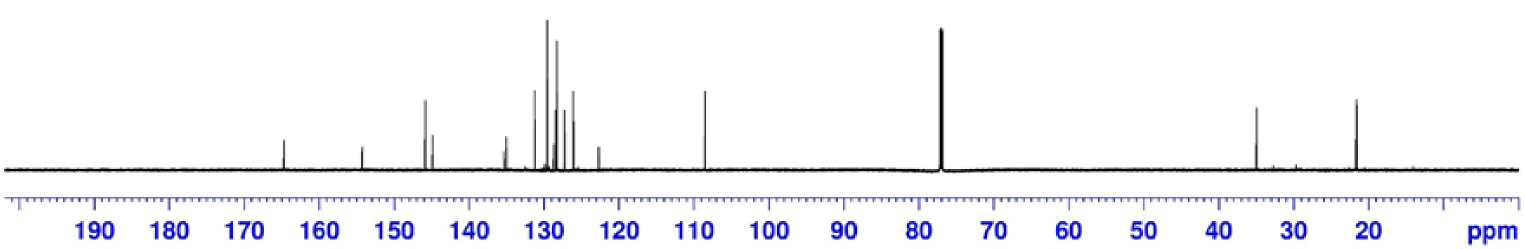

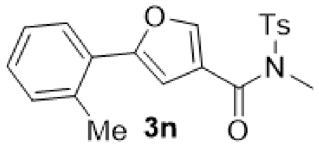

3n

^{1}H NMR ($CDCl_3$, 700 MHz)

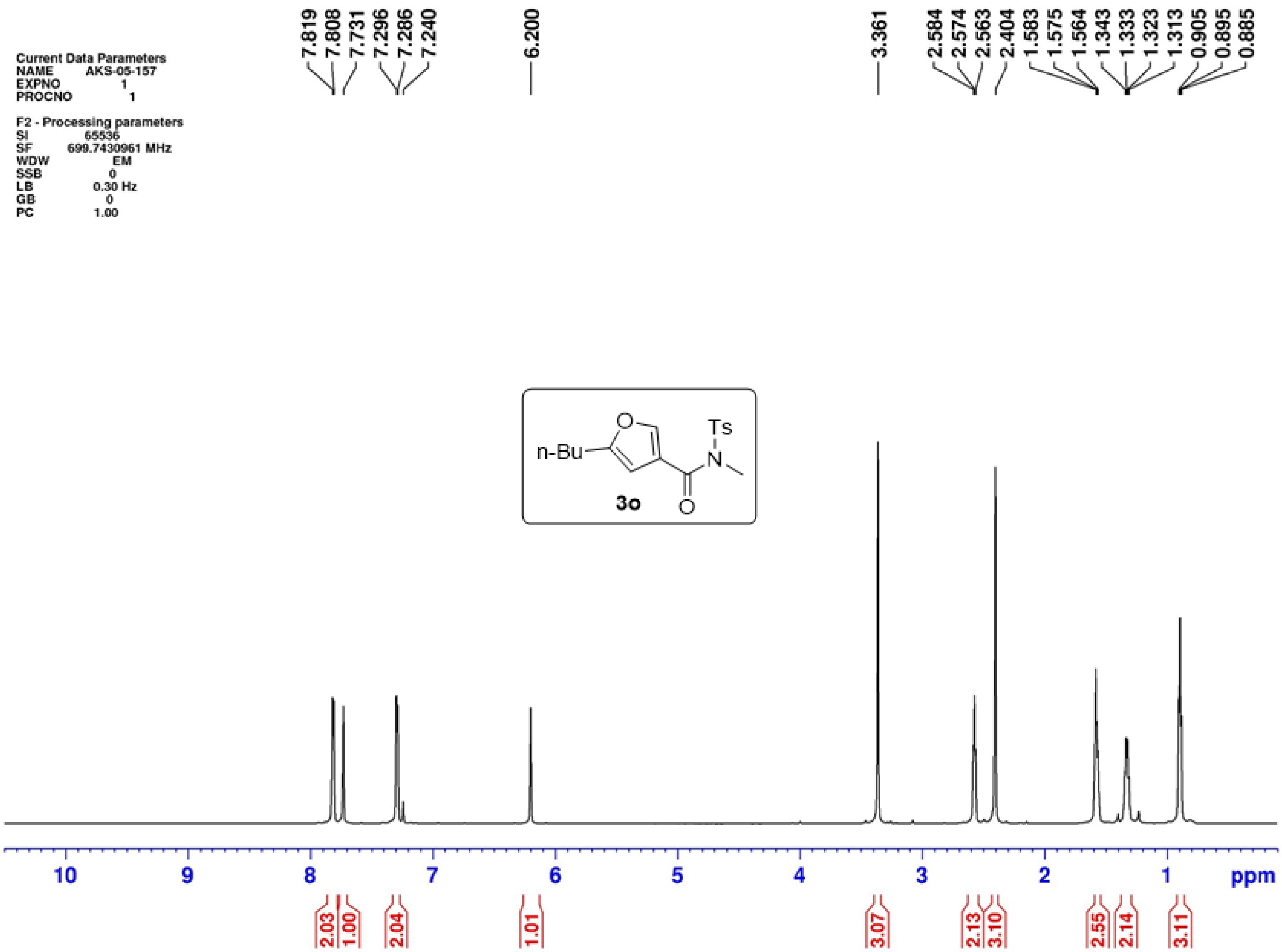

$^{13}C\{1H\}$ and DEPT NMR ($CDCl_3$, 175 MHz)

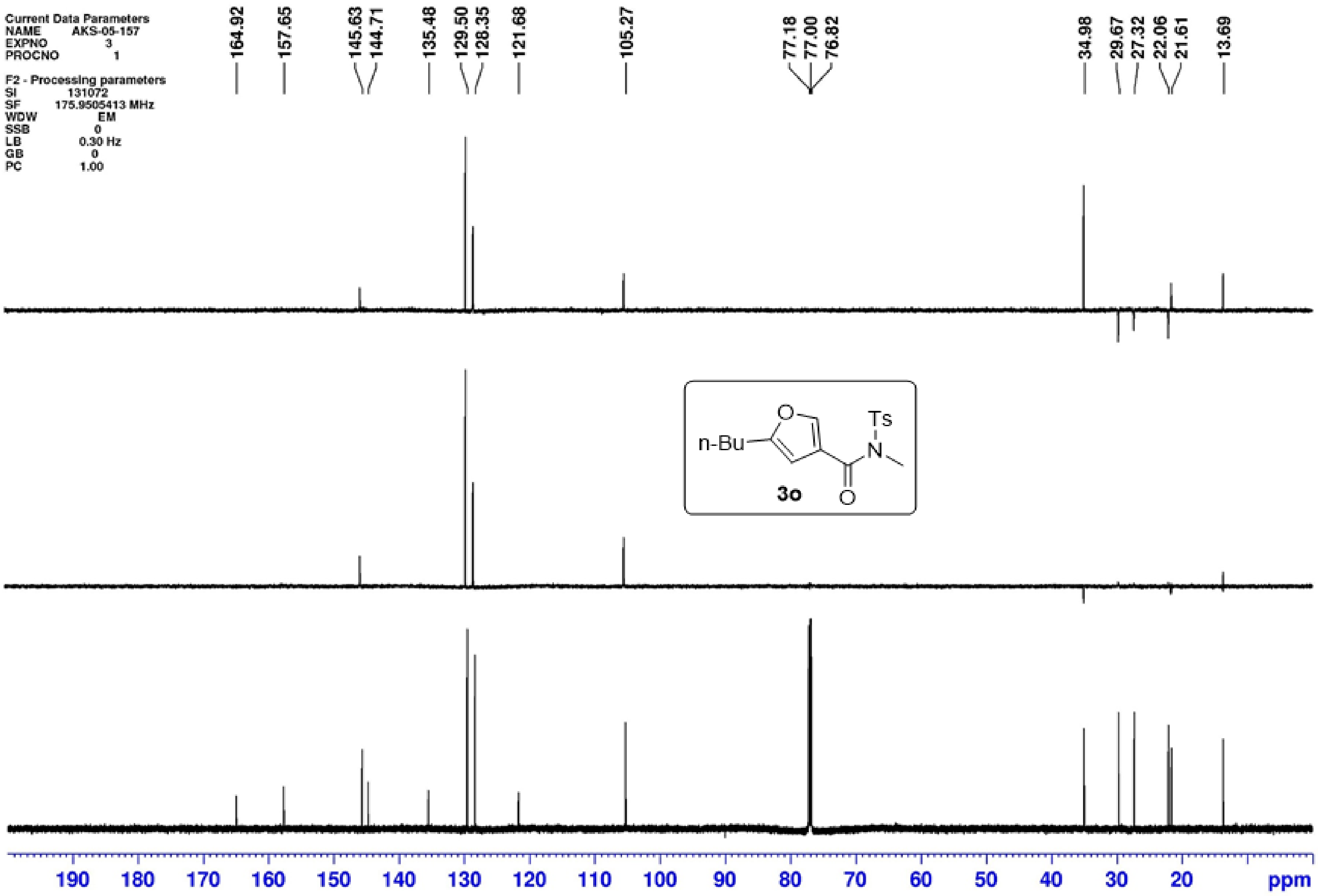

^{1}H NMR ($CDCl_3$, 700 MHz)

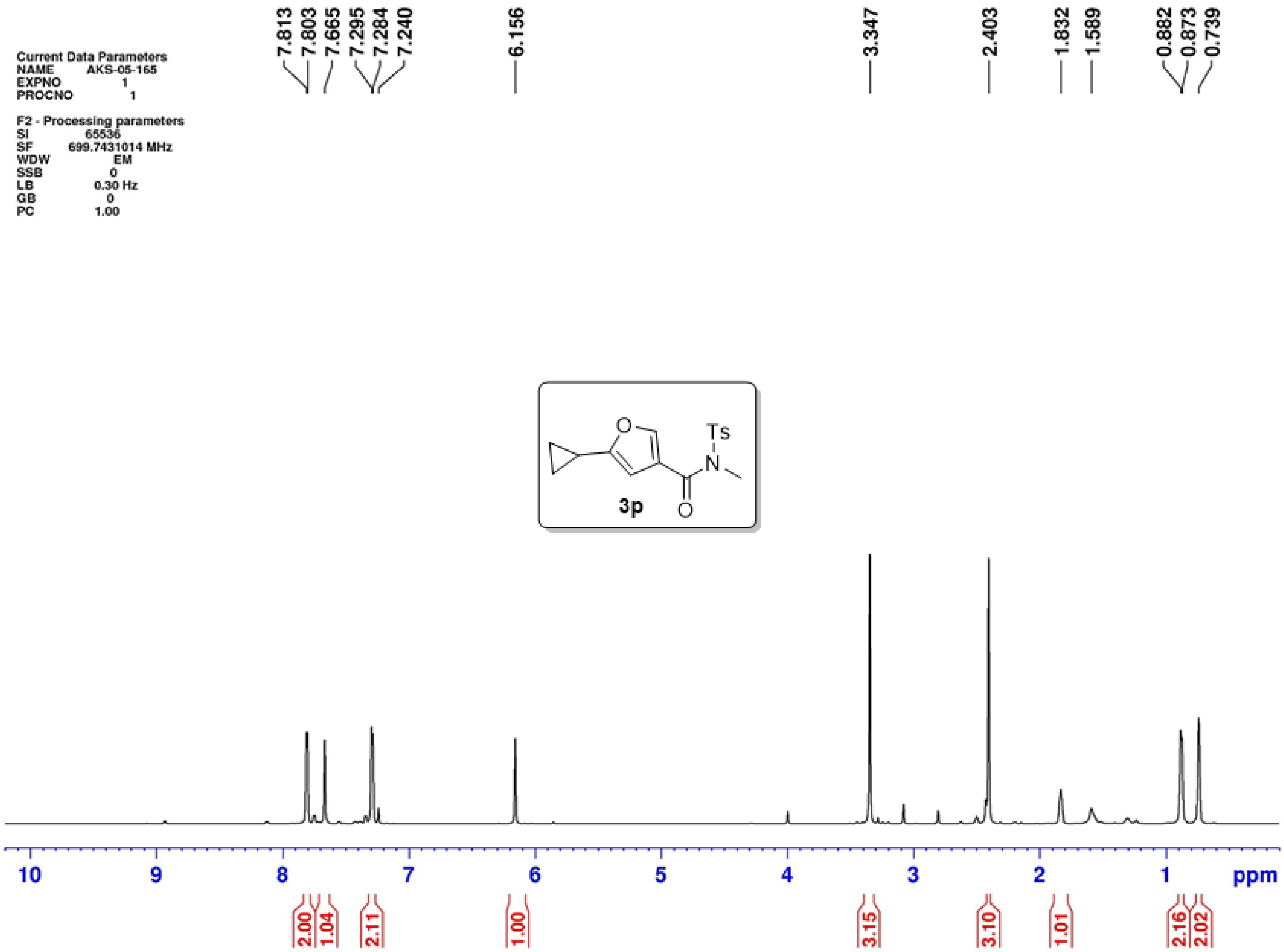

$^{13}C\{1H\}$ and DEPT NMR ($CDCl_3$, 175 MHz)

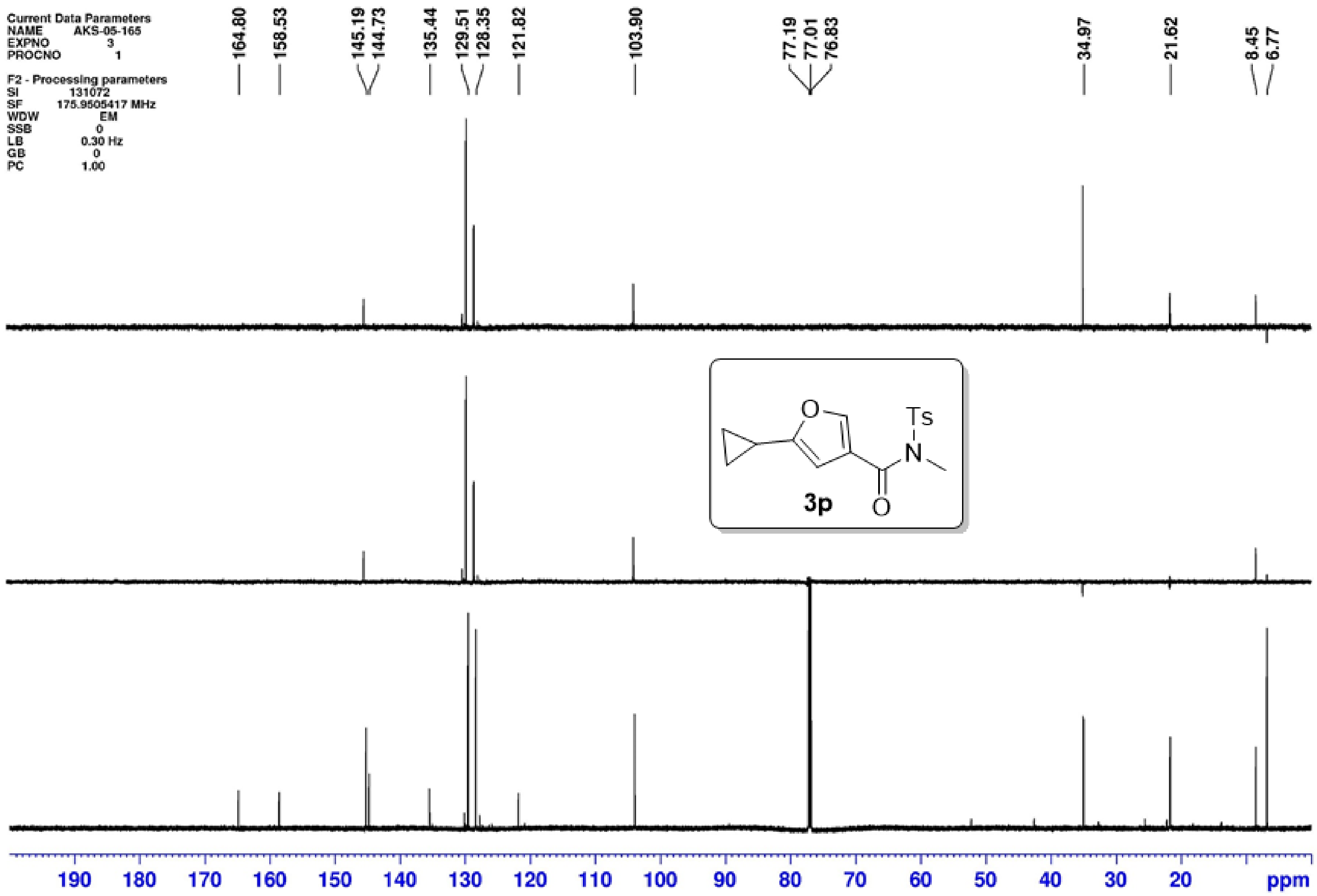

^{1}H NMR ($CDCl_3$, 700 MHz)

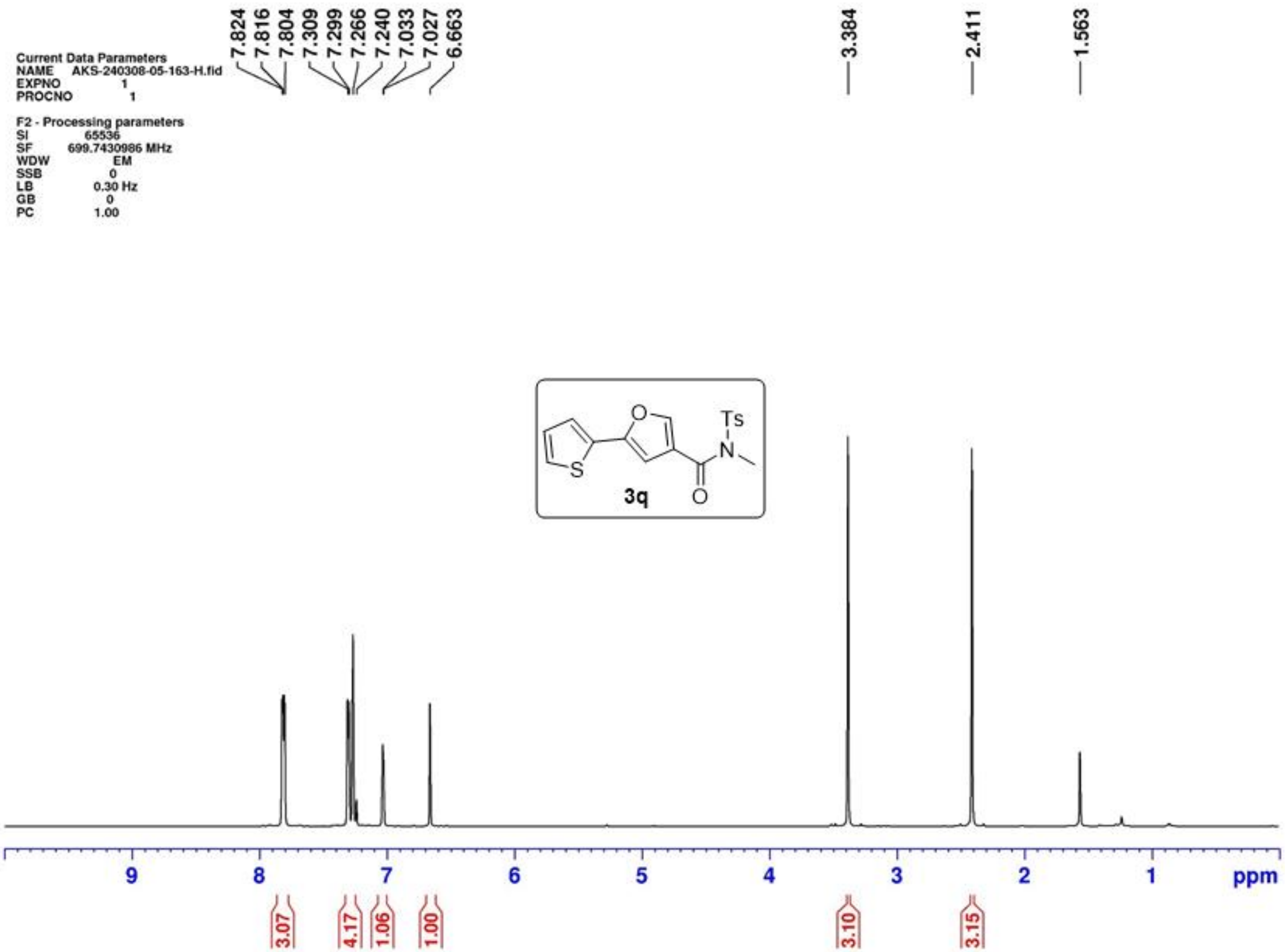

$^{13}C\{1H\}$ and DEPT NMR ($CDCl_3$, 175 MHz)

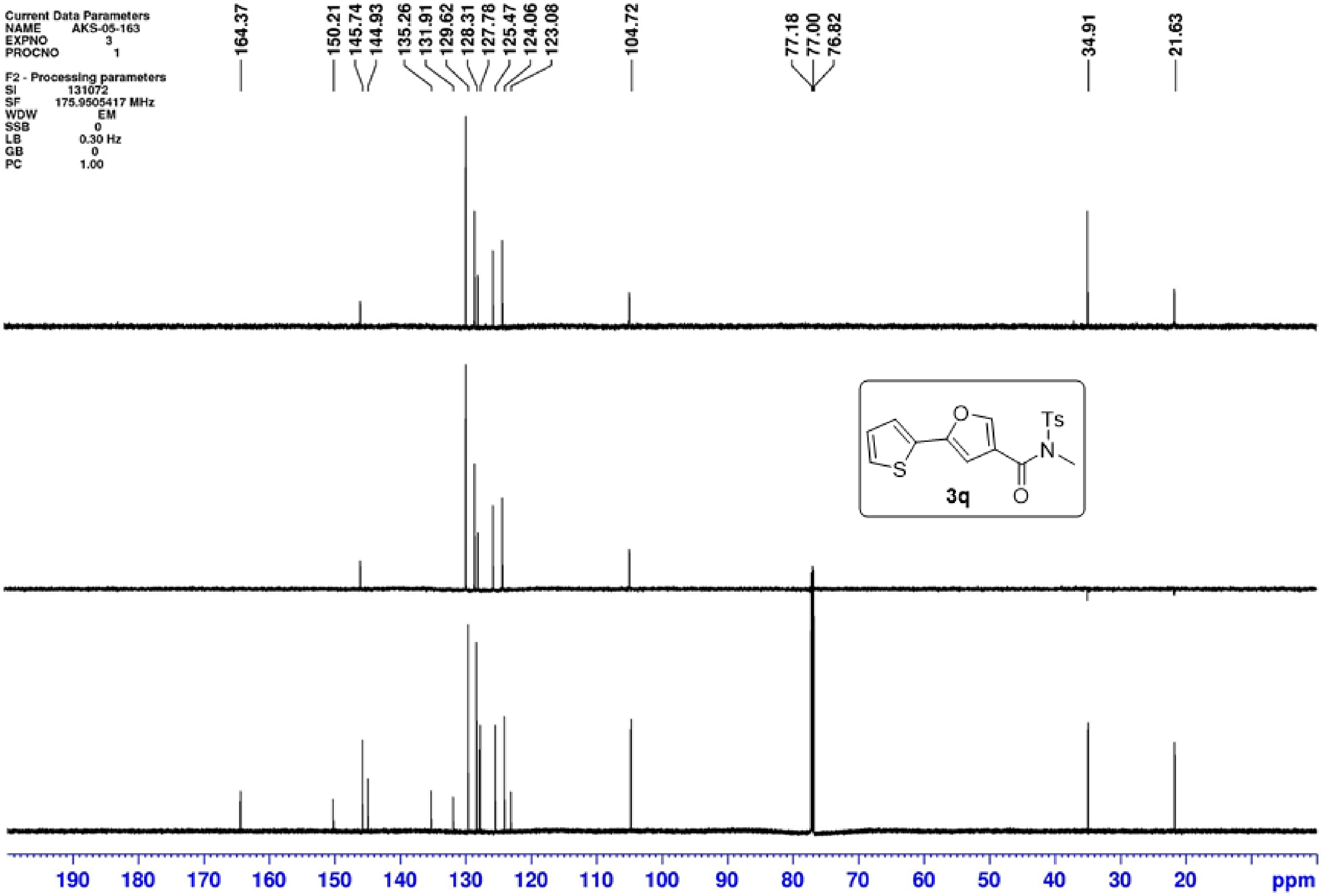

^{1}H NMR ($CDCl_3$, 700 MHz)

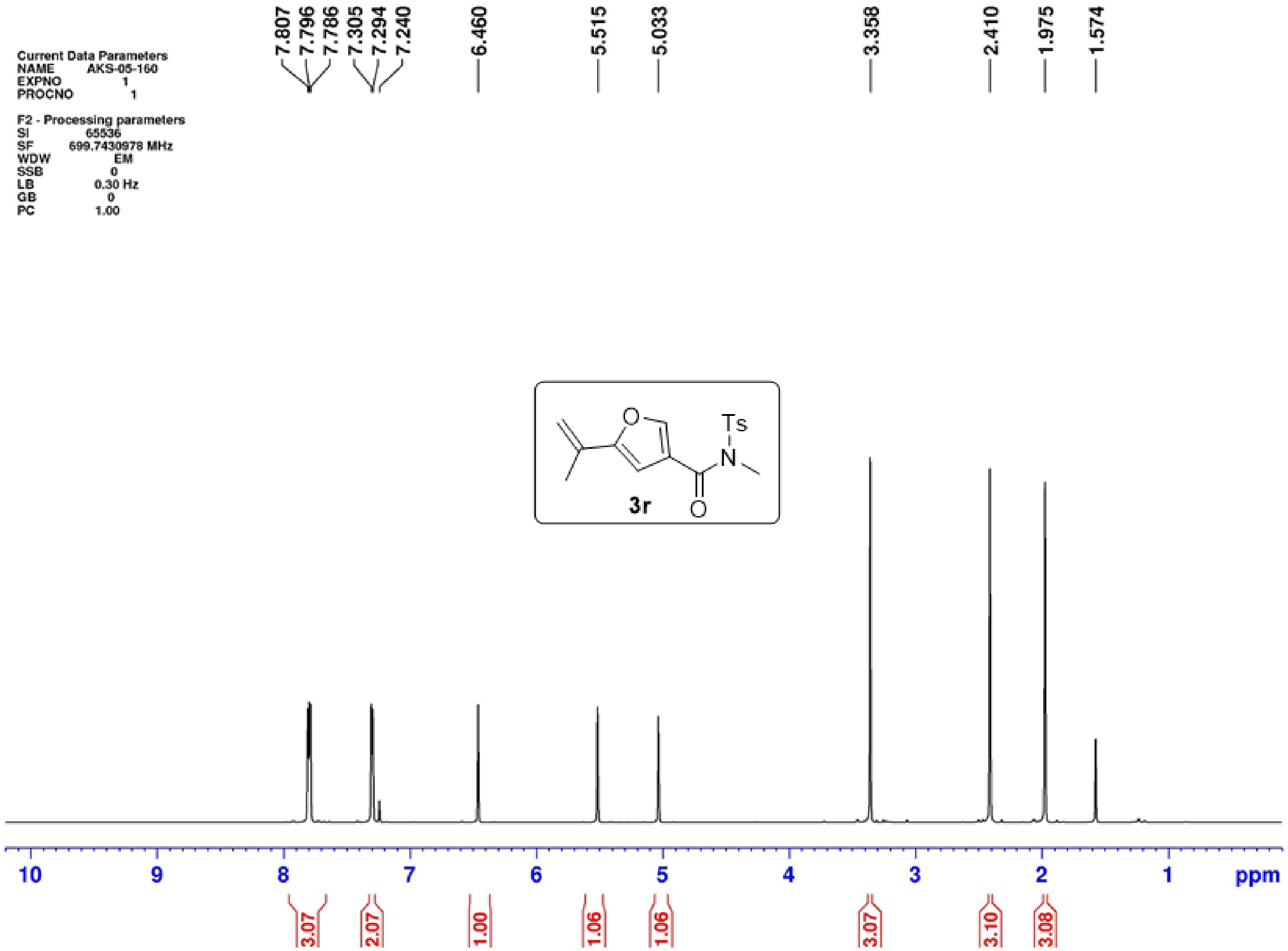

^{13}C{1H} and DEPT NMR ($CDCl_3$, 175 MHz)

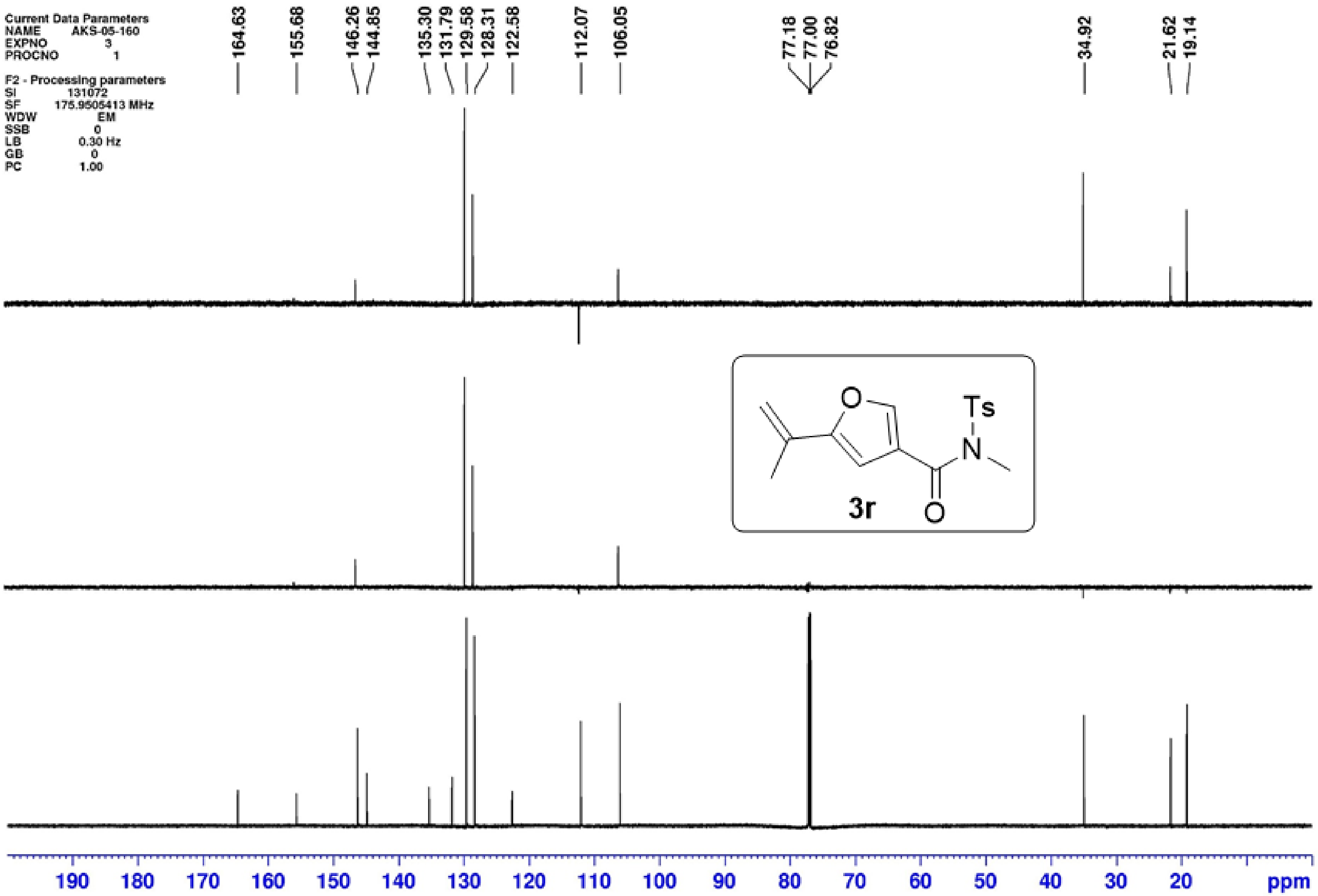

^{1}H NMR ($CDCl_3$, 700 MHz)

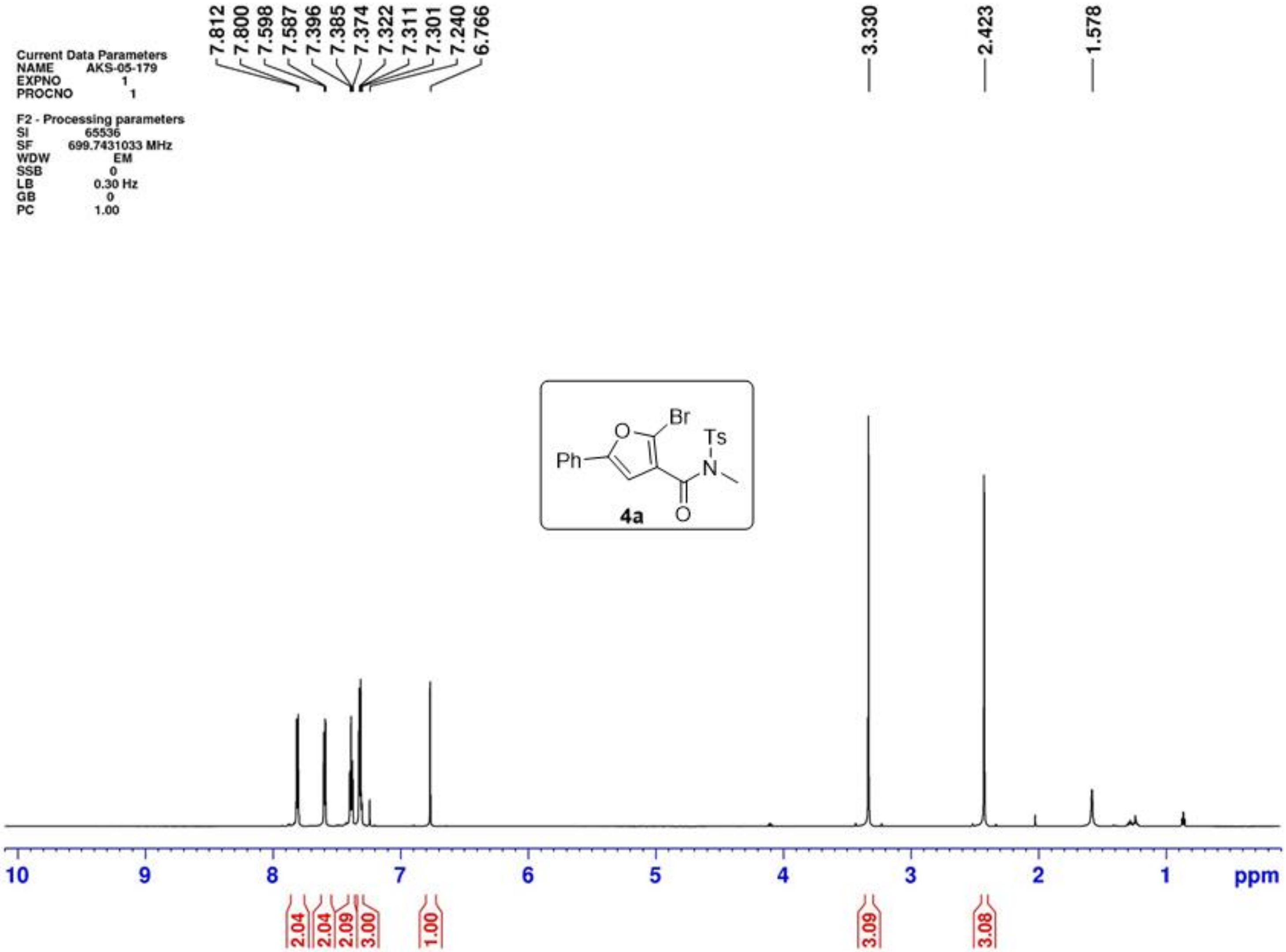

$^{13}C\{1H\}$ and DEPT NMR ($CDCl_3$, 175 MHz)

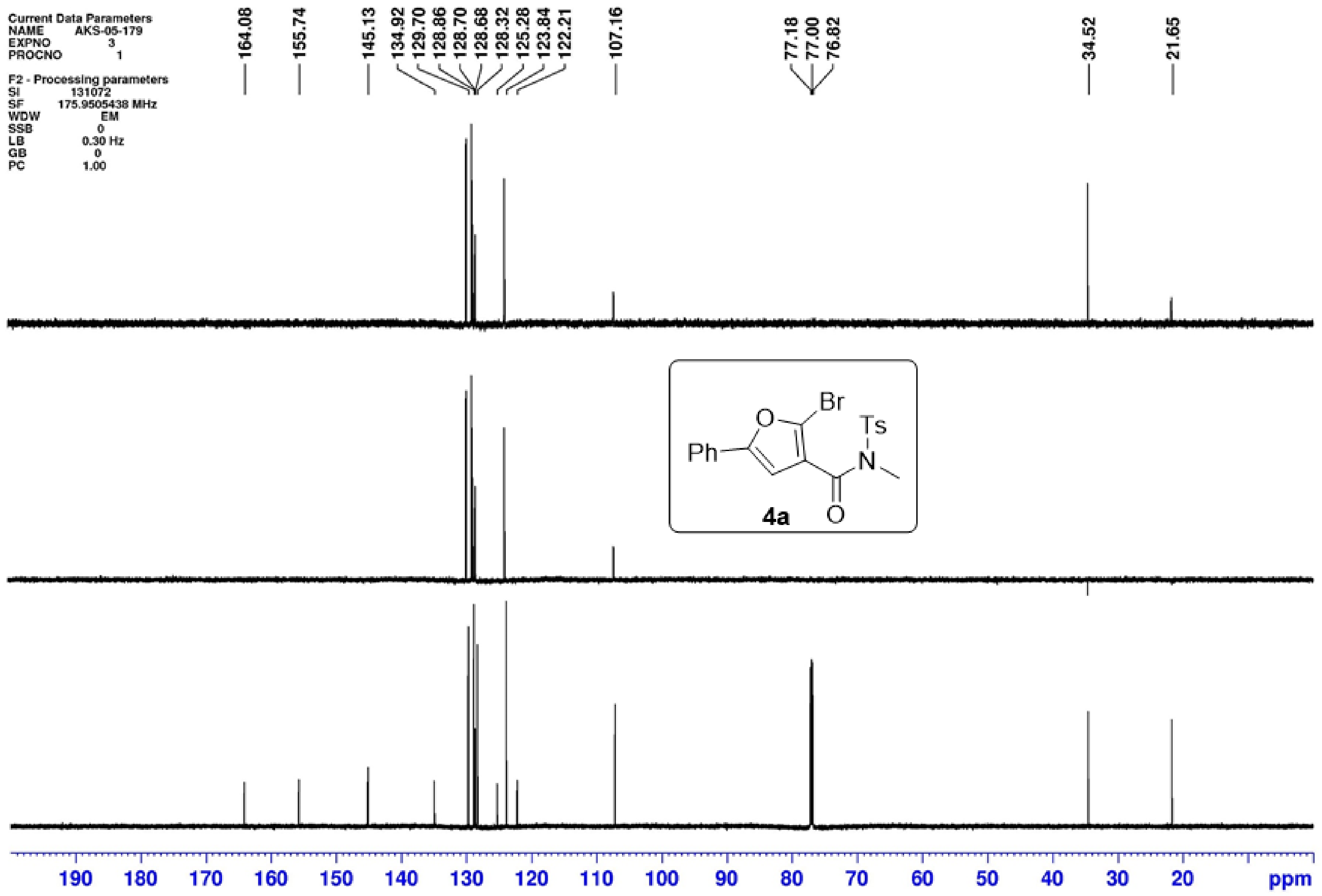

^{1}H NMR ($CDCl_3$, 700 MHz)

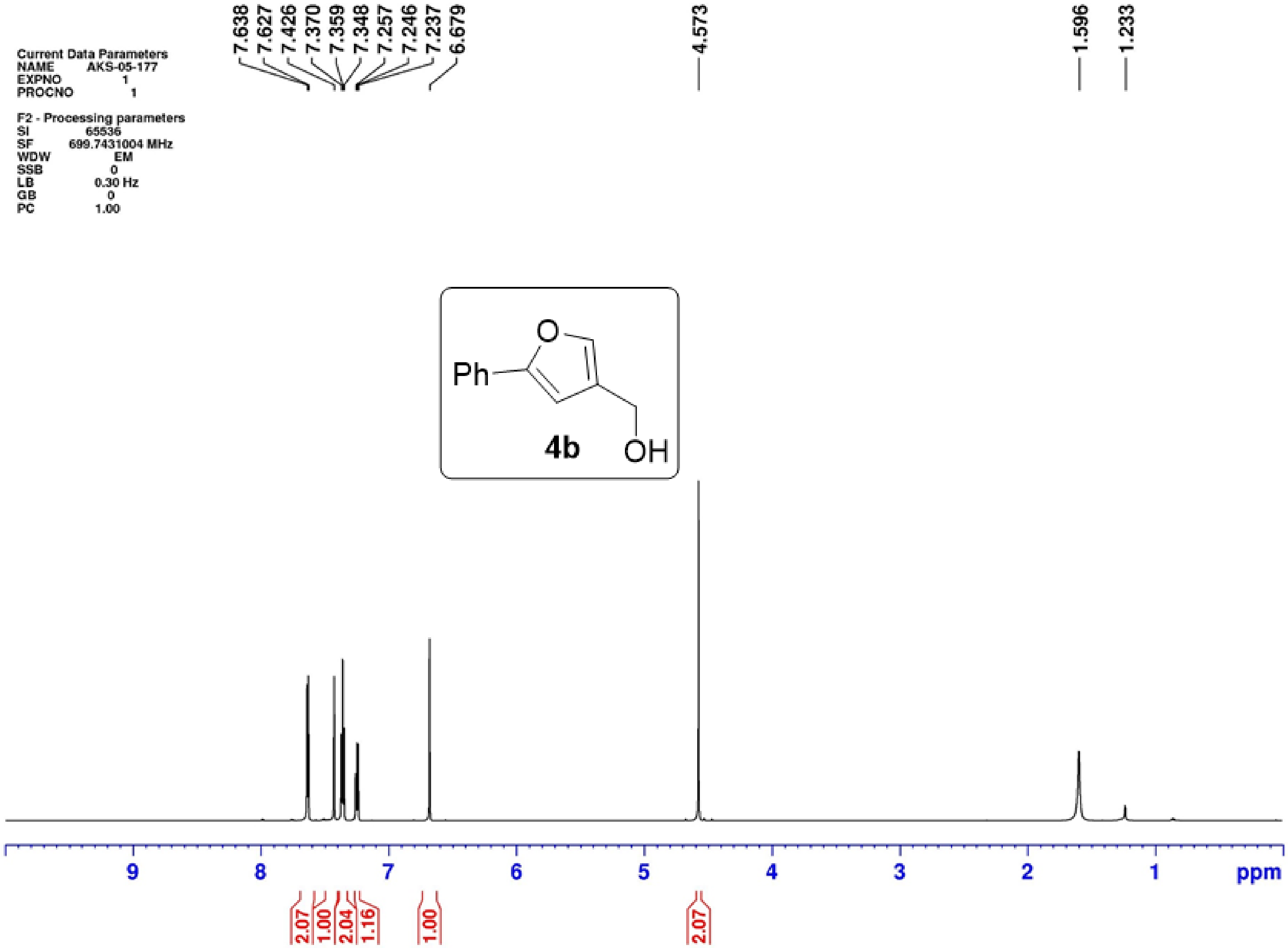

$^{13}C\{1H\}$ and DEPT NMR ($CDCl_3$, 175 MHz)

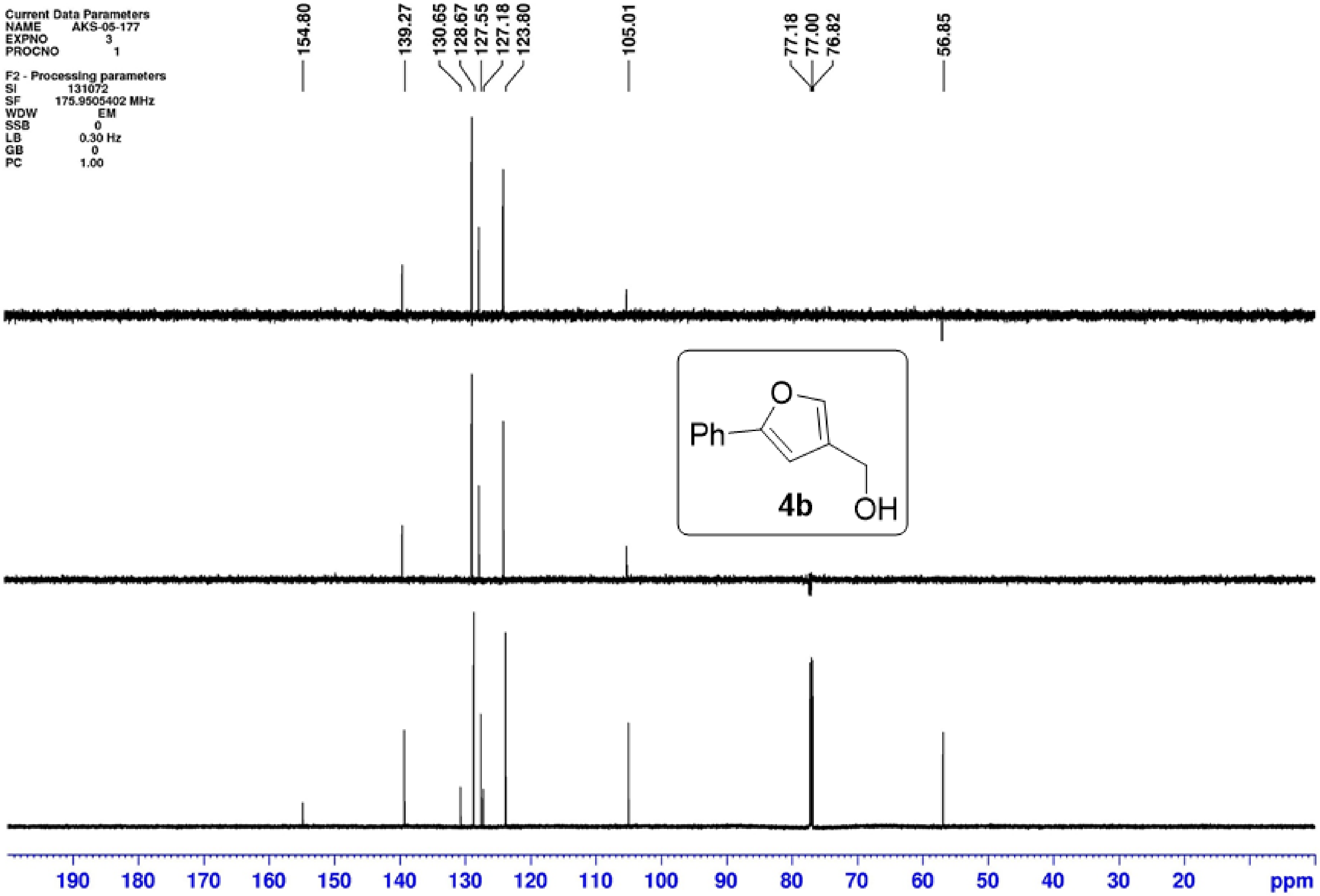

^{1}H NMR ($CDCl_3$, 700 MHz)

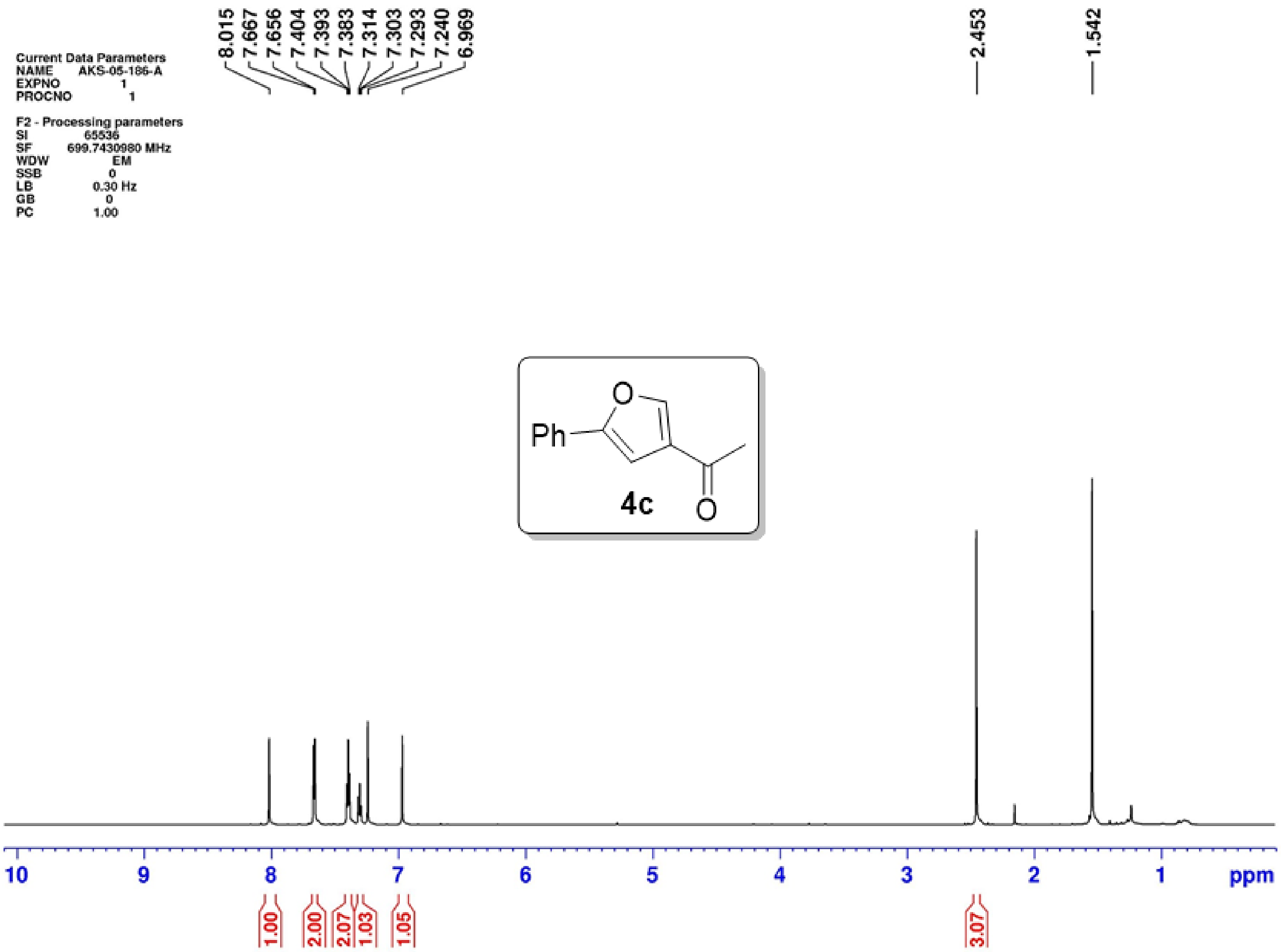

4c

$^{13}C\{1H\}$ and DEPT NMR ($CDCl_3$, 175 MHz)

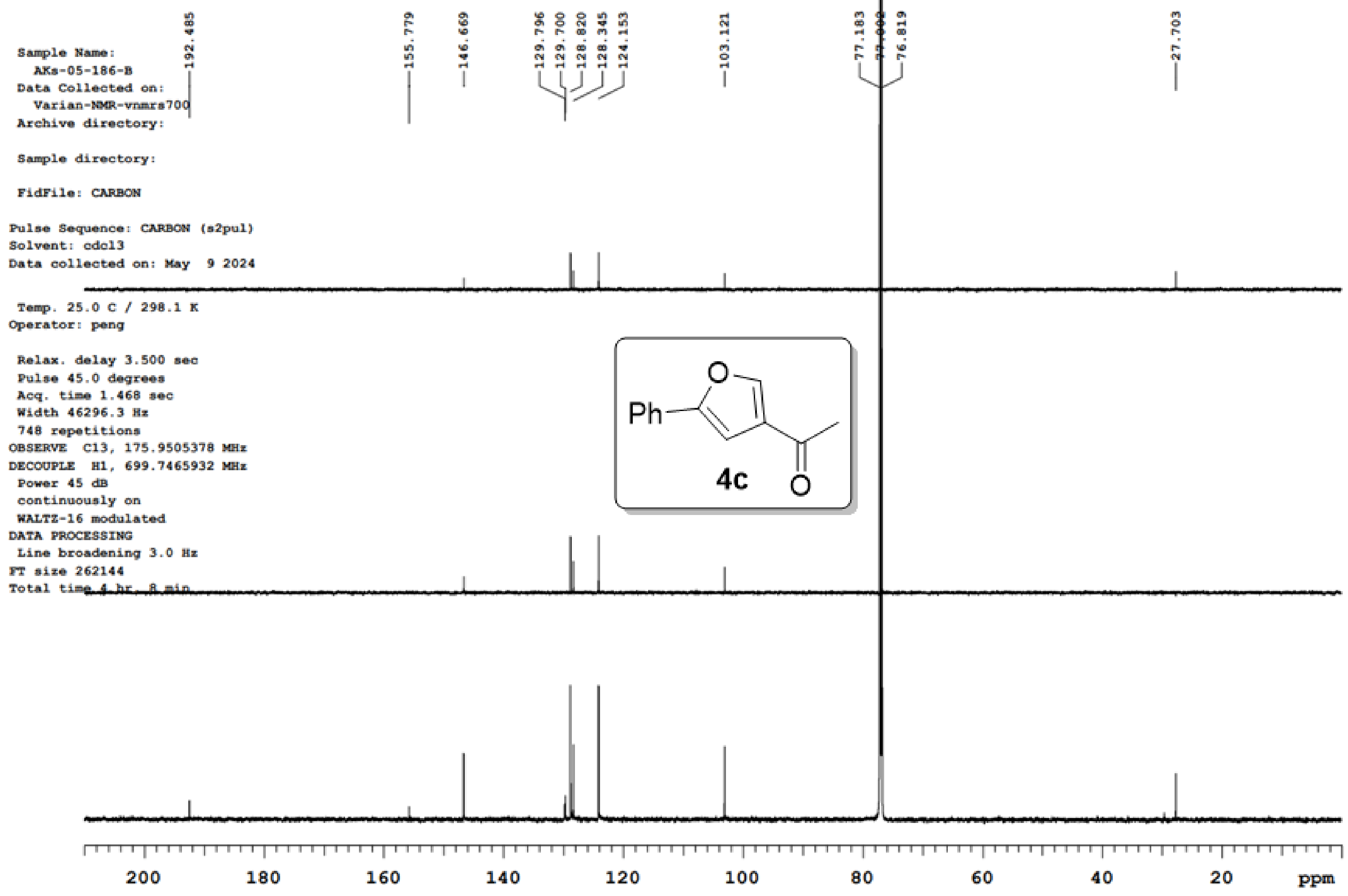

^{1}H NMR ($CDCl_3$, 700 MHz)

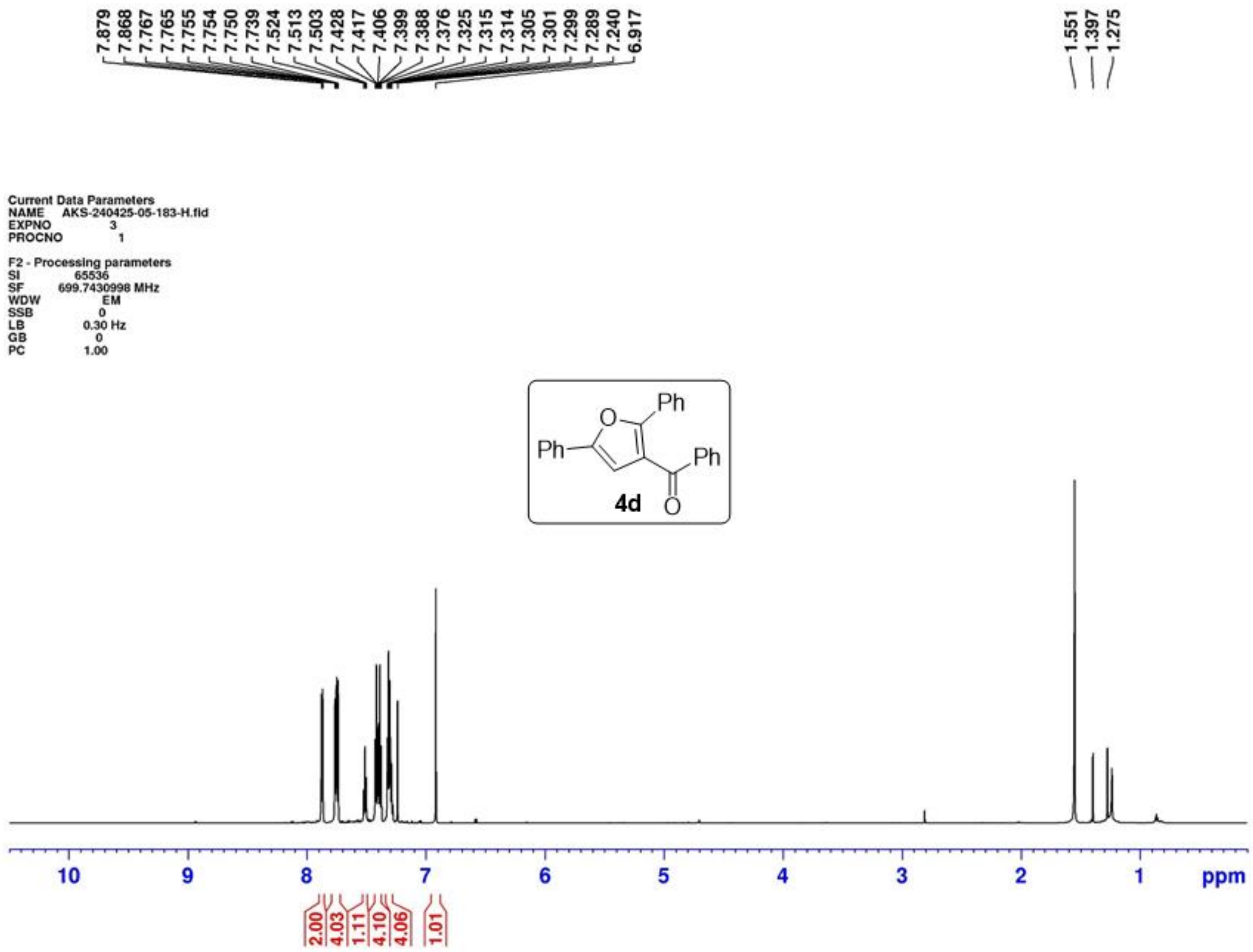

$^{13}C\{1H\}$ and DEPT NMR ($CDCl_3$, 175 MHz)

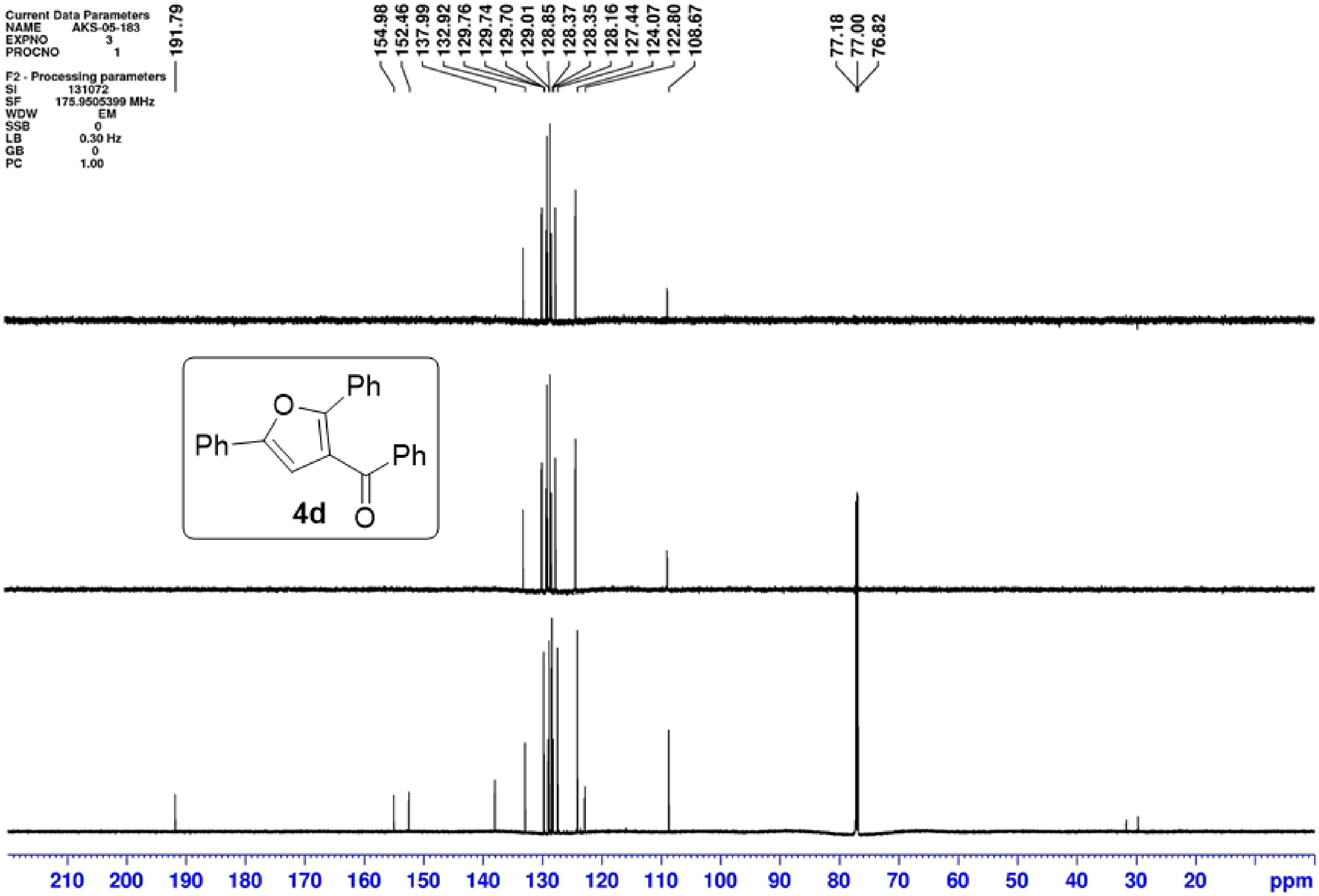

Supplement: Supplementary file 1 — jo4c02096_si_001.pdf [file jo4c02096_si_001.pdf]
